# Niche theory‐based modeling of assembly processes of viral communities in bats

**DOI:** 10.1002/ece3.7482

**Published:** 2021-04-03

**Authors:** Fabiola Nieto‐Rabiela, Oscar Rico‐Chávez, Gerardo Suzán, Christopher R. Stephens

**Affiliations:** ^1^ Facultad de Medicina Veterinaria y Zootecnia UNAM Coyoacan Mexico; ^2^ C3 ‐ Centro de Ciencias de la Complejidad UNAM Coyoacan Mexico; ^3^ Instituto de Ciencias Nucleares UNAM Coyoacan Mexico

**Keywords:** co‐occurrence, disease ecology, functional diversity, Jaccard similarity, Niche Theory, phylogenetic diversity, viral biogeography

## Abstract

Understanding the assembly processes of symbiont communities, including viromes and microbiomes, is important for improving predictions on symbionts’ biogeography and disease ecology. Here, we use phylogenetic, functional, and geographic filters to predict the similarity between symbiont communities, using as a test case the assembly process in viral communities of Mexican bats. We construct generalized linear models to predict viral community similarity, as measured by the Jaccard index, as a function of differences in host phylogeny, host functionality, and spatial co‐occurrence, evaluating the models using the Akaike information criterion. Two model classes are constructed: a “known” model, where virus–host relationships are based only on data reported in Mexico, and a “potential” model, where viral reports of all the Americas are used, but then applied only to bat species that are distributed in Mexico. Although the “known” model shows only weak dependence on any of the filters, the “potential” model highlights the importance of all three filter types—phylogeny, functional traits, and co‐occurrence—in the assemblage of viral communities. The differences between the “known” and “potential” models highlight the utility of modeling at different “scales” so as to compare and contrast known information at one scale to another one, where, for example, virus information associated with bats is much scarcer.

## Graphical abstract

We show how viral communities that are associated with bats respond to different environmental and host filters, such as trophic guild, size, phylogeny, and spatial distribution. We show, for instance, that the degree of similarity between viral communities decreases as a function of the degree of overlap of their geographic distributions as measured by the following statistical diagnostic.

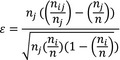

## 1 INTRODUCTION AND PURPOSE

A great deal of effort has been invested in trying to understand the distribution and function of the different biota at multiple scales, with the aim of predicting community assembly rules and the consequences of their complex interactions (Cavender‐Bares et al., 2009). Developing such an understanding has important implications in the case of viral disease dynamics and outbreak occurrence, as it would provide us with useful tools for inferring both the causes and consequences of viral distributions, as well as aiding the decision‐making process in the prevention, monitoring, and control of infectious diseases (Pedersen & Fenton, 2006). However, in the case of communities of symbionts (organisms associated with other organisms, generally parasites) comparatively little is known about their assembly processes. In this case, it is evident that host characteristics could act as strong filters on the assembly of pathogen communities (Dallas & Presley, 2014; Nieto‐Rabiela et al., 2018). For instance, those ecological behaviors and physiological properties that allow symbionts to associate with a particular host in the first place (Luis et al., 2015).

Furthermore, Gay et al., (2014) have suggested that the risk of disease transmission to humans may depend on the pathogen richness found in natural reservoir species. In this case, multiple co‐infections and host species infection dynamics are important features for understanding the mechanisms of emerging diseases (Gay et al., 2014; Mihaljevic, 2010; Wolfe et al., 2005). Hence, studies that adopt a multisymbiont approach are necessary in disease ecology in order to understand patterns and processes at different levels and to identify the role of species diversity in the dynamics of infection (Aivelo & Norberg, 2018; Chan et al., 2015; Dallas & Presley, 2014; Rendón‐Franco et al., 2014). Additionally, multisymbiont interactions, both interspecific (mixed helminth infections) and nonspecific (genetic strains of microparasites), manifest themselves in a complex way (Bordes & Morand, 2011; Graham, 2008). For instance, symbiont interactions may explain variability in infection risk better than other factors, such as age or seasonality, due to the fact that such interactions can facilitate or hinder colonization of the host (Bolling et al., 2012, 2015; Bordes & Morand, 2011; Furuya‐Kanamori et al., 2016; Newman et al., 2011; Seabloom et al., 2015). Thus, a one‐by‐one interaction analysis may lead to results that offer a poor representation of the underlying, more complex, multisymbiont reality, especially given that many host–symbiont interactions are intimately embedded within communities of organisms. In short, it is clearly necessary to understand the full ecological context of infection and transmission (Johnson et al., 2016; Suzán et al., 2015; Woolhouse, 2001). Furthermore, understanding the mechanisms that determine the structure of symbiont communities in their hosts is essential for designing better disease control programs (Johnson et al., 2016; Mihaljevic, 2012; Rynkiewicz et al., 2015).

Bats are an useful model for exploring the processes that determine the co‐occurrence of symbionts, since a wide variety of symbionts have been associated with them, including viruses, fungi, parasites, and bacteria (Allocati et al., 2016; Whitaker Jr. et al., 2009; Wibbelt et al., 2009). In addition, it is well known that these symbionts may co‐occur in bat communities (Banskar et al., 2016; Presley, 2011; Tello et al., 2008). However, most co‐occurrence studies have focused on ectoparasites and bacteria (Wilkinson et al., 2016). Fewer studies have considered the potential interactions of different viruses (Anthony et al., 2017; Nieto‐Rabiela et al., 2018, 2019), where the potential for spillovers, such as the current SARS‐CoV‐2 pandemic, is important with their consequent effect on public health.

The objective of this paper is to determine whether the assemblage of viral communities of bats is linked in a statistically significant way to one or more environmental filters and to determine the relative weight/importance of each filter type in the assemblage of viral communities. We will examine this hypothesis in the context of two different data sets: a “known” data set, where only reports from Mexico were used, and a “potential” data set, which included virus reports from all of the Americas, but restricted to only those bat species that are present in Mexico, but which have not necessarily been identified as hosts of a given virus there.

The three types of filter we will consider are phylogenetic, functional, and spatial. This is not an exhaustive set (Nieto‐Rabiela et al., 2018), and each type can encompass a large number of intrinsic and extrinsic factors. Also, these variables are not completely independent. Nevertheless, each variable type represents a different class of filter:

1) The phylogenetic filter offers a reciprocal understanding between the evolution of the hosts and their symbionts over evolutionary timescales (Córdova‐Tapia & Zambrano, 2015; Krasnov et al., 2014; Streicker et al., 2010). As it provides information about when each pair of hosts was separated in evolutionary time (Graham & Fine, 2008), it allows us to develop a better understanding of the coevolution of bats and their symbiont communities (Cavender‐Bares et al., 2009). For example, if bats with the same common ancestor are found to share a virus, it is suggestive of the fact that the virus was present in the common ancestor. However, other factors are not ruled out, such as the physiological similarity between hosts that could facilitate association with the same viruses.

2) The functional filter can represent the fact that some symbionts are present in hosts that share life histories (Becker et al., 2020; Davies & Pedersen, 2008; Luis et al., 2015). We chose as specific variables associated with this category: trophic guild, body mass, litters per year, and number of pups per year, as these have previously been associated with viral diversity and have a corresponding poor association with phylogeny (Bordes et al., 2008; Córdova‐Tapia & Zambrano, 2015; Kamiya et al., 2014; Rico, 2015) and therefore have less chance of being confounded by phylogenetic characteristics.

3) The spatial filter represents the fact that species must co‐occur in order to pass infections and share symbionts (Krasnov et al., 2010; Webb et al., 2002). The importance and quantification of co‐occurrence has been observed, for example, in the case of Leishmaniasis (Stephens et al., 2016, 2009), where, relative to a null hypothesis, the degree of co‐occurrence may take positive, negative, or random values depending on the type of interaction it proxies (competition, cooperation, etc.) and the dispersion capability between the pair of species considered (Griffith et al., 2016).

With these variables in mind, we generate a model to explain the similarity between the viral communities of different host pairs calculated as a response variable. Our initial hypothesis is that in the assembly of viral communities in Mexican bats, “niche,” as opposed to “neutral,” processes predominate, where, by “niche,” we mean that there exist, either direct or indirect, factors that affect the presence or absence of a given pathogen. Our environmental filters can be thought of as such niche factors. With this in mind, we can hypothesize how these filters may affect viral community differences across different bat species. For instance, we might expect to see greater differences in viral communities between two bat species that are phylogenetically distant as opposed to closely related. Similarly, we may expect to see similar viral communities among those bat species that co‐occur spatially. The motivation for this hypothesis is that species that have high contact rates with hosts will be more susceptible to acquire these new viruses when coexisting with them and therefore allow their adaptation and later their association (Stephens et al., 2016, 2009; Woolhouse et al., 2005). Finally, we may expect to see more similar communities between bat species that share functional traits, such as trophic guild. We will examine these hypotheses in the context of our two data sets, where one would expect to see them to be valid across both “known” and “potential” data sets and their corresponding models.

## 2 METHODS

We constructed a database using all reports of viruses isolated or detected by molecular techniques from the American continent and associated with Mexican chiropters. The information used was that reported by Rico‐Chávez et al., (2015) in their PCR analyses with bat samples obtained from the south of Mexico and information available online at DBatVir (http://www.mgc.ac.cn/DBatVir/). Two models were tested: a “known” model (Appendix 1), where only reports from Mexico were used, and a “potential” model (Appendix 2), which included virus reports from all of the Americas, but restricted to only those bat species that are present in Mexico, but which have not necessarily been identified as hosts of a given virus there. We use both models as each has its own advantages and disadvantages. For instance, the “known” model has the defect that positives may be undersampled, as well as reflecting “local” collection biases. However, it does represent the present state of knowledge in Mexico, while the “potential” model represents a larger sampling effort, but extrapolates data from outside Mexico to Mexico. We use the word “potential” as, in this case, we are assuming that a bat/virus association identified outside Mexico for a bat with geographic distribution in Mexico could also be present there. This could occur, for instance, if the interaction reflects the physiological capability of the species to be a host, or that the niche conditions that favor the interaction outside Mexico are also present in Mexico.

For each model, a virus–host adjacency matrix, cataloged as the presence/absence of the virus in each host species, was constructed. The associated Jaccard index (Cooper et al., 2012; Jaccard, 1922; Real & Vargas, 1996) was calculated using the vegan library in free software R (Oksanen et al., 2016; R Core Team, 2017), with a value range between 0, corresponding to completely different viral communities, and 1, corresponding to identical viral communities for each host pair.

**FIGURE 1:**
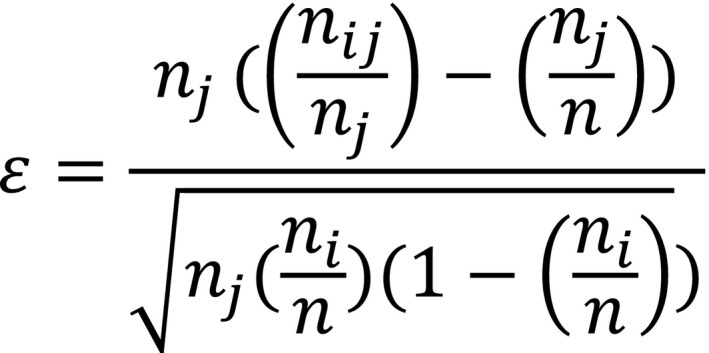
Epsilon formula as a measure of co‐occurrence. nj is the number of cells where the species j is distributed, nij is the number of cells where both species are distributed, and n is the total number of cells

For the filters, the spatial filter was represented using a co‐occurrence measure, ε, a binomial test with the corresponding formula shown in Figure 1 (Stephens et al., 2009). ε measures the statistical significance of the difference between the conditional probability, P(C|X), for two bat species C and X to co‐occur, relative to the null hypothesis, P(C). These probabilities are calculated by laying a uniform grid of N cells on the geographic area of interest and then calculating P(C) = *N*(C)/*N*, P(X) = *N*(X)/*N* and P(C|X) = *N*(CX)/*N*(X), where *N*(C) and *N*(X) are the number of cells with the presence of the bat species C or X, respectively, and *N*(CX) is the number of cells with the presence of both. Presence or no presence of the different hosts was evaluated using point collection data from the C3/CONABIO platform SPECIES (http://species.conabio.gob.mx/) using a uniform grid of 6,473 cells of area 20km^2^. In this database, the collections reported by Mexican research institutes are registered, so there are 128,071 records of chiropterans that have been collected from 1889 to the present along with their sampling site. The denominator in ε is the standard deviation of the binomial distribution. In the case where this distribution may be approximated by a normal distribution, |ε | > 1.96 corresponds to the standard 95% confidence interval that the codistribution of C and X is not consistent with the null hypothesis that they are distributed independently. The importance of using ε as opposed to P(C|X) directly is that the latter can introduce spurious correlations in the case where *N*(X) is small.

As a phylogenetic filter, the phylogenetic distance between hosts was calculated and the results were normalized using the formula: log (x) – log (ẋ), where we calculate the logarithm of the phylogenetic distance for a pair of species and subtract the logarithm of the mean phylogenetic distance. This value was extracted from the mammalian super tree (Bininda‐Emonds *et al*., 2007) using the library picante (Kembel et al., 2010) implemented in R (R Core Team, 2017).

For the functional filter, the characteristics evaluated were trophic guild and body mass (Bordes et al., 2008; Han et al., 2015; Luis et al., 2015; Rico‐Chávez et al., 2015). The information was obtained using PanTHERIA (Jones et al., 2009), Animal Diversity Web (http://animaldiversity.org), and the Encyclopedia of Life (http://eol.org/). The trophic guild was classified as “(1) herbivore (not vertebrate and/or invertebrate), (2) omnivore (vertebrate and/or invertebrate plus any of the other categories), and (3) carnivore (vertebrate and/or invertebrate only)” (Jones et al., 2009). We assigned the value one to a pair of hosts when the trophic guild was the same and zero when it was different. For body mass, we used two metrics, the absolute difference between the pair of bats and the average body mass in grams of the pair. The rationale for this is that a positive correlation between body mass and symbiont biodiversity has been reported such that the greater the body mass, the greater diversity of symbionts (Bordes et al., 2008; Han et al., 2015; Luis et al., 2015). We also considered the number of litters per year and the number of pups per year. However, for bat species within our database, these variables showed no predictive value for viral community similarity and therefore were discarded from further analysis. The absence of an effect here may be due to the fact that they are not especially variable traits among bat species.

After corroborating the normality of our variables, we subsequently computed the relation between the Jaccard similarity index as response variable and centralized values of the explanatory variables: phylogeny (phylogenetic distance), functional traits (body mass difference and trophic guild), and ε as a spatial variable—using a generalized linear model (GLM) (Venables & Ripley, 2002). We used the GLM function (Venables & Ripley, 2002) and an automated selection of the best model using the stepAIC function of the MASS 7.3‐39 library (Venables & Ripley, 2002; Ripley, 2013) which tests for all the potential interactions in the model selection and uses the Akaike information criterion (AIC) to select the best model (Burnham & Andersonl, 1992). Both were implemented in the R software (R Development Core Team, 2011).

Of course, in the case of both models, there are multiple data biases, some of which we have discussed above, that can affect the models. Associated with these, sampling effort can play a role. One way of gauging this is by considering the number of papers in the literature with respect to a given virus‐bat pair. This could then potentially be used as a variable in the GLM to determine its effect. We hope to return to this in a future publication.

## 3 RESULTS

### 3.1 “Known” model

The “known” database, where only reports in Mexico were used, consisted of 19 bat species and 6 associated viral species, where 11 different communities were detected. We tested all the potential interactions between the 5 filters and the following best model was found: similarity ~ trophic guild X body mass, with an AIC = 63.27. Table 1 shows the best three models, along with a “null” model that consists of using only the intercept, with their corresponding AIC and R^2^ values. The differences between one model and another are small.

**TABLE 1:** AIC and *R* ^2^ comparison for the top three GLM models from the “known” model and a “null” model corresponding to the intercept only

MODEL	*R* ^2^	AIC
Similarity ~ Intercept (Null model)		76.15
Similarity ~ Phylogenetic + Epsilon + Trophic guild × Average body mass	0.07	65.5
Similarity ~ Epsilon + Trophic guild × Average body mass	0.02	64.36
Similarity ~ Trophic guild × Average body mass	0.02	63.27

We observed that no individual filter was significant at the 95% confidence level (Table 2). The phylogenetic and ε filters have no significant influence on the similarity between symbiont communities between bat species, with the values 0.36 and 0.19, respectively. However, the functional filter was significant, with a value of 0.0001 (*p* <.05) for the variable trophic guild x body mass which had a regression coefficient of −0.15.

**TABLE 2:** Coefficients of the GLM in the best “known” model

	Estimate	Std. Error	t value	Pr(>|t|)
Intercept	0.2127	0.0376	6.485	1.6 × 10^−9^
Phylogenetic	0.0578	0.0631	0.915	0.3618
Epsilon	0.0036	0.0028	1.289	0.1998
Trophic guild	−0.0445	0.0541	−0.823	0.4120
Average body mass	−0.0461	0.0237	−1.943	0.0541
Trophic guild X Body mass	−0.1501	0.0381	−3.937	0.0001

### 3.2 “Potential” model

The database associated with the potential species consisted of 55 bat species and 12 associated viral species, where 22 different communities were detected. The best model was found to be: similarity ~ phylogenetic + epsilon + trophic guild + average body mass + difference in body mass, with an AIC = 384.74 (Table 3). In contrast to the “known” model, all filters are significant at the 95% confidence level (Table 4). The intercept is 0.50 with *p* < 2 × 10^‐16^. The phylogenetic filter has a regression coefficient of −0.003 (*p* < 2 × 10^‐16^), the negative coefficient indicating that greater phylogenetic distance is correlated with less similarity. The trophic guild variable has a regression coefficient of 0.26 (*p* < 2 × 10^‐16^), the positive relation indicating that bats from the same trophic guild have more similar viral communities. The spatial filter, ε, has a regression coefficient of −0.007 (*p* = 3.34 × 10^–13^), indicating that the more two bat species co‐occur, the less similar are their viral communities. Finally, the regression coefficients for the average body mass and difference in body mass terms were −0.0028 and 0.0032, respectively, with corresponding p values 0.001 and 0.0146. Although with *p* values <.05, their statistical significance relative to the other coefficients is much less. The results show that similarity decreases slightly with increasing average body mass and decreases slightly with increasing difference in body mass.

**TABLE 3:** AIC and *R* ^2^ comparison for the top 2 GLM models from the “potential” model and a “null” model corresponding to the intercept only

Model	*R* ^2^	AIC
Similarity ~ Intercept (Null model)		1,021.6
Similarity ~ Phylogenetic + Epsilon + Trophic guild + Body mass	0.35	440.82
Similarity ~ Phylogenetic + Epsilon+Trophic guild + Average body mass + Difference body mass	0.36	384.74

**TABLE 4:** Coefficients of the GLM in the best “potential” model

	Estimate	Std. Error	t value	Pr(>|t|)
Intercept	0.5002	0.0346	14.443	<2 × 10^−16^
Phylogenetic	−0.0035	0.0002	−13.957	<2 × 10^−16^
Epsilon	−0.0079	0.0009	−8.323	<2 × 10^−16^
Trophic guild	0.2638	0.0159	16.502	<2 × 10^−16^
Average body mass	−0.0028	0.0008	3.835	0.0001
Difference body mass	0.0032	0.0011	−2.443	0.0146

## 4 DISCUSSION

The mere fact that a potential host is physiologically susceptible to a symbiont does not mean that it is, indeed, a host and, even less, that it is an epidemiologically important host. The successful transmission of a virus between individuals depends on the co‐occurrence of several variables, both in space and in time. For instance, although a bat species may be physiologically capable of acquiring the virus, if the bat and the virus do not co‐occur in space and time, then the bat cannot be infected. Of course, although co‐occurrence is a necessary condition for viral infection, it is not sufficient, as a bat species may have a high contact rate but may be very difficult to infect (Allocati et al., 2016; Anthony et al., 2017; May, 2006; Streicker et al., 2010; Webber et al., 2016; Wey et al., 2008). For instance, a bat may be infected but produce insufficient infectious particles to transmit them to another species, or it may produce them in the wrong body compartment for transmission (Viana et al., 2014). Furthermore, a bat's immune system may reject the virus, thereby inhibiting transmission. However, in all these cases a viral mutation may occur that now favors transmission. Thus, the importance of co‐occurrence of susceptible hosts remains as a fundamental factor in analyzing actual and potential transmission risk.

Of course, many underlying factors affect the transmission probability. Some are intrinsic to the species, such as the particularities of its immune system (Ezenwa & Jolles, 2011), where cellular receptors may or may not be recognized by the virus. Others include biochemical mutations, such as those found in the fur or secretions of a potential host, that may reject or facilitate the infection (Bordes & Morand, 2011; Brook & Dobson, 2015; Presley, 2011; Rynkiewicz et al., 2015; Wang & Crowled, 2015).

Other factors that can play an important role in the transmission process are those associated with the behavioral and functional traits of the species, such as its trophic guild, where shared food may harbor infection and act as a fomite favoring interspecific transmission of a virus. Another example is migration, where elevated metabolic rates in some hosts can maintain an infection and therefore favor its transmission to other species.

In short, pathogen transmission, both intra‐ and interspecific, is highly multifactorial, involving many variables, among which are phylogenetic, functional, life history, or other ecological and environmental factors, that can also affect contact rates, by influencing where a host or vector is to be found in space and time and in what concentration. Of course, there are many other potential variables. As a complex adaptive system, the vector–host–pathogen transmission network represents many different interactions that are subject to environmental and host pressures and which lead to effects that can amplify or cancel one another, thus making it difficult to isolate one interaction from another (Bordes & Morand, 2011; Presley, 2011; Rynkiewicz et al., 2015; Wang & Crowled, 2015).

Although our emphasis in this paper is on identifying and quantifying the effect of several representative filters on assembly processes of viral communities in bats, theory, such as niche theory and the corresponding null model—neutral theory (Chave, 2004; Hubbell, 2005)—can also help us to interpret and understand the impact of these filters on the development of community structure. Although, at first sight, these filters may seem antagonistic, they may also alternate, or complement each other, depending on the spatial scale under consideration (Chase & Myers, 2011). The neutral theory, for example, proposes that species distributions are random. Thus, if the distribution of viruses is random, similarities will not be found between the characteristics of those bats that host them. In contrast, in the case of niche theory, it has been recognized that species that have common ancestry are similar and, in particular, have similar ecology (Racey, 2011; Sober & Steel, 2017). Hence, as a result of this shared ancestry, species may exhibit similar abiotic tolerances, that then contribute to the species niche and influence the set of hosts which may be infected by a given community of symbionts (Graham & Fine, 2008; Krasnov et al., 2010). So, if a filter is a predictive variable for community similarity, we would argue that this is most naturally interpreted in the context of niche theory, not neutral theory.

The correlation between the similarity of the viral communities and the phylogenetic, functional, and spatial filters that describe their hosts differs significantly between the known and potential models. Both are consistent with niche theory, in that there is at least one filter that is significantly correlated with viral community diversity (see Tables 2 and 4).

In the “known” model, the only statistically significant niche variable is actually a composite variable—average body mass x trophic guild—and is of the functional type. Average body mass itself is very close to the 95% level, with a p‐value of 0.0541. The corresponding regression coefficient is −0.0461, showing that the greater the average mass of the species pair, the smaller the Jaccard index and therefore the greater the dissimilarity in their viral communities. Body mass has been identified previously as a predictor of the richness and abundance of symbionts (Arneberg et al., 1998; Bordes et al., 2008; Lindenfors et al., 2007). In this case, the intuition is that the larger the animal, the greater the capacity for hosting a greater diversity of pathogens. Of course, this does not mean a priori that two species with large body mass should have viral communities that differ more than two species with small body mass.

For trophic guild, one might expect that the relation with viral similarity is influenced by the host communities’ own assembly, as manifested in the fact that a nested assembly has been observed to increase the complexity of the trophic guild (from herbivores to carnivores) within Phyllostomids in Chiapas, Mexico (de la Peña‐Cuéllar et al., 2012).

Finally, in the “known” model, neither the spatial nor the phylogenetic filters are significantly correlated with viral community similarity. In the case of phylogeny, this could be explained by observing that, in the data of the known model, the Phyllostomidae family predominates, and this can skew the potential influence of phylogeny, as the bat species considered are mostly grouped within this single family, which therefore represents a poor sampling of the underlying phylogenetic diversity in Mexico. Indeed, the known model is mostly characterized by a lack of significant filters. We believe this is largely due to the nature of the data of the known model, which covers a much smaller set of species that are biased both with respect to their phylogeny and their spatial distribution. This is an important reason as to why the potential data set was considered.

In contrast to the known model, in the “potential” model, all three environmental filters are involved: phylogenetic, functional, and spatial, with all considered filters being statistically significant. Phylogeny is now very significant (*p* < 2 × 10^‐16^), which can be attributed to the substantial increase in the number of bat species in the model, thereby increasing the degree of observable phylogenetic variation, which is now more widely distributed among five host families: Antrozoidae, Molossidae, Mormoopidae, Phyllostomidae, and Vespertilionidae. With phylogeny, we observe that its regression coefficient is negative, as we expected, showing that the greater the phylogenetic distance between two bat species, the less similarity in their viral communities.

Interestingly, in terms of the spatial variables implicit in ε, the regression coefficient is also negative. This means that viral communities are more dissimilar, the greater the degree of overlap between species. At first sight, this seems to be counterintuitive and, contrary to our original hypothesis, relative to the intuition that more overlap should lead to more contact. A potential explanation of this result is that species of bats that are very similar (phylogenetically and functionally) tend to compete by the niche exclusion principle, something that is not beneficial for either of the species. In order to avoid competition, they may separate spatially. However, although now spatially separated, the similarity in their viral communities is the signature that they coexisted ancestrally. Of course, this is only a hypothesis that must be further studied and validated using other data. Additionally, it neglects the possibility that niche differentiation may occur in situ, that is, that two species may find ways to occupy sufficiently different niches even though they are present in the same spatial areas. Another hypothesis is that the dissimilarity in viral communities with co‐occurring hosts is a way for the virus communities themselves to avoid competition.

**FIGURE 2:**
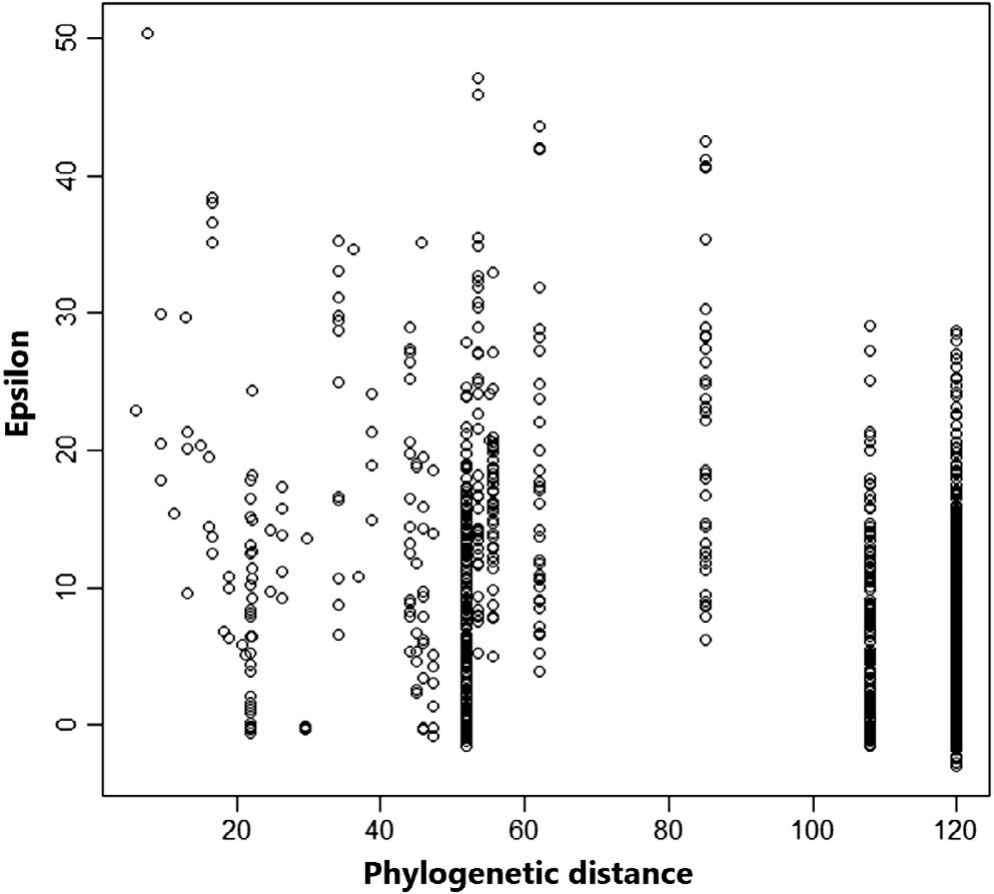
Relationships between phylogenetic distance and values of epsilon

When we analyzed the clustering of viral communities by host genus within the phylogenetic tree (Figure 2), we found that many species within the same genus were associated with smaller values of ε. This is consistent with the fact that the conformation of viral communities represents historical processes, with adaptation taking place over many thousands or millions of years, unlike "mediated" processes, such as in the case of the individual analysis of viruses. However, this is for the moment a hypothesis that must be further tested.

In addition, it is possible that species with higher values of ε, corresponding to higher degrees of co‐occurrence, are habitat generalists, with nonspecific niches and ample geographic distributions such that there are no particularly important, direct biotic interactions between them. However, they will coexist with a larger number of more habitat‐specialized species with more restricted niches and geographic distributions and with which they may share symbionts. Thus, we hypothesize that the lower values of ε associated with the species of a given genus could also be attributable to them being specialists. Of course, this discussion does not negate the importance of co‐occurrence but rather posits that its interpretation has subtleties when taken as a proxy for potential biotic interactions. Therefore, we conclude that the viral communities associated with bats are more similar when the spatial distribution of the hosts does not significantly overlap.

Trophic guild is now significant in its own right, where we see that viral community similarity is great, the more similar the trophic guilds of the bat species. As mentioned, this could be due to the fact that shared food may harbor infection and act as a fomite favoring interspecific transmission of a virus.

Both average body mass and difference in body mass of the bat species pair are statistically significant at the 95% confidence level, but their degree of significance is much less than that of the other filters.

Thus, we conclude that the “potential” model can be used to predict hosts that share, to a certain degree, their viral communities by considering their similarity in trophic guild, co‐occurrence, phylogeny, and body mass, information that can be used for future research and the search for new, unsampled hosts.

Although only six of the twelve considered virus species have been detected in Mexican bats, as mentioned, one cannot be sure that this represents a true absence of the other viral agents. One might then conclude that it is necessary to carry out much more extensive and exhaustive monitoring in order to know the real host range of a given virus. However, we believe that the “potential” model can help in this respect by indicating which bat species are likely to be hosts and to provide a better understanding of the assembly of viral communities.

The results we have presented are based on currently available information, which, however, may contain several biases, such as toward synanthropic species, or to those competent reservoir species for infectious pathogens that are of public and animal health concern. Our study, therefore, highlights the importance of designing models so that true negatives, in the sense of a statistical inference model, can be determined which, in turn, would allow us to analyze such biases.

The use of two data sets, each associated with a different spatial scale, also illustrates the potential pitfalls that stem from biased data sampling when considering emerging or re‐emerging diseases. All else being equal, the potential model should offer a better estimation of the viral assembly process, as it is associated with a larger sample than the known model. However, potentially different data collection biases can affect conclusions at distinct spatial scales. The point collection data for bat species is, we believe, substantially less biased than the data for virus, particularly for the known model.

We believe that comparing and contrasting models derived from different data sets, and different spatial scales, is a useful way for analyzing relative data biases in the different sets. The subsequent analysis of the models and consistency with hypothesis can then be used as a way of estimating degrees of data bias. Thus, the fact that the known model, in contrast with the potential model, did not indicate that viral community diversity depended on our chosen filters is a result, we would argue, of data bias in that model, where the data were skewed toward southern Mexico and the Phyllostomid family of bats, as well as a result of small sample size. This is not to say that there is no bias in the potential model per se but, rather, that criteria can and should be developed to be able to detect data biases.

## 5 CONCLUSION

The environmental filters we have considered here are correlated with the assembly of the viral communities that are associated with Mexican chiropterans. This is consistent with niche theory. In other words, viral communities respond to host filters (functional and phylogenetic), as well as environmental (spatial) filters. However, the significance of each filter is dependent on data biases, sample size, and potentially other variables, as manifested in the differences between the “known” and “potential” models. Further research is clearly required at distinct scales to determine how the different filters change their significance from one scale to another.

The differences between the “known” and “potential” models highlight the utility of modeling using different data sets associated with different spatial scales so as to integrate and leverage known information at one scale to another different one where, for example, virus information may be much scarcer, as is the case with bats here. Such information gaps could be bridged by considering predictive variables that are applicable to arbitrary spatial scales and then using them to predict the degree of similarity between viral communities of different potential hosts.

This approach to understanding the assembly and distribution of viral communities offers a greater understanding of the underlying ecological dynamics when compared to more epidemiological approaches which concentrate more on viral interactions such as interference, superinfection, and coinfection.

Generally, in public and animal health, as well as disease ecology, simple models are usually employed, that may end up yielding biased results, which then translate into inefficient conservation or public health policies. Here, we wish to stress that the whole viral association history behaves in a complex and dynamic way. As such, these simple models should be principally used in order to identify those individual factors that most influence the behavior of the system, which can then be incorporated into more complex models that more accurately reflect the phenomenon being studied (González, 2016). Such results then serve as a basis for further studies and predictive models that can take into account variables, such as trophic guild and co‐occurrence, as relevant factors in virus–host associations.

## CONFLICT OF INTEREST

None declared.

## AUTHOR CONTRIBUTIONS

**Fabiola Nieto‐Rabiela:** Conceptualization (equal); Data curation (lead); Formal analysis (lead); Investigation (equal); Methodology (equal); Project administration (lead); Writing‐original draft (lead). **Christopher Rhodes Stephens:** Conceptualization (equal); Formal analysis (equal); Funding acquisition (equal); Investigation (equal); Methodology (equal); Project administration (equal); Writing‐original draft (supporting). **Gerardo Suzan:** Conceptualization (supporting); Writing‐review & editing (equal). **Oscar Rico:** Methodology (equal); Writing‐review & editing (supporting).

## Supporting information

Appendix S1

Appendix S2

## Data Availability

The authors confirm that the data supporting the findings of this study are available within the article and its supplementary material and also are available in the repository https://doi.org/10.5061/dryad.msbcc2fxq.
