# Supplementary material for: Niche theory‐based modeling of assembly processes of viral communities in bats
Source: Ecol Evol. 2021 Apr 3;11(11):6305–14. doi: 10.1002/ece3.7482 (PMC8207334; doi:10.1002/ece3.7482)
Supplement: Supplementary file 1 — Appendix S1 [file ECE3-11-6305-s001.pdf]

A. Bats' characteristics of Known model

X	Y	Similarity	dist.phylo	spacial	epsilon	score	trophic.guild	body.mass	dif.B.M
Artibeus.jamaicensis	Artibeus.lituratus	0.25	7.6	438	50.4	3.22	0	17.7	5.5
Carollia.brevicauda	Carollia.perspicillata	0.25	11.2	104	42.3	3.86	0	4.4	11.5
Artibeus.lituratus	Desmodus.rotundus	0	62	356	42.02	2.61	0	26.3	10.1
Artibeus.jamaicensis	Desmodus.rotundus	0.5	62	387	41.93	2.64	0	8.6	16.1
Artibeus.lituratus	Pteronotus.parnellii	0.5	85	293	41.2	2.91	0	39.8	5.52
Artibeus.jamaicensis	Pteronotus.parnellii	0.5	85	315	40.66	2.95	0	22.1	3.1
Desmodus.rotundus	Pteronotus.parnellii	0.33	85	298	40.63	2.89	1	13.5	24.7
Pteronotus.davyi	Pteronotus.parnellii	0.5	36.2	152	34.63	2.62	1	10.02	2.3
Artibeus.lituratus	Glossophaga.commissarisi	0	53.4	137	31.92	3.59	0	50.23	6.1
Artibeus.jamaicensis	Glossophaga.commissarisi	0	53.4	144	30.78	3.67	1	32.53	7.7
Artibeus.lituratus	Pteronotus.davyi	1	85	152	30.32	3	0	49.82	17.73
Artibeus.lituratus	Carollia.brevicauda	0.33	44	147	30.13	3.05	1	44.6	13.8
Artibeus.jamaicensis	Pteronotus.davyi	0.25	85	159	28.98	2.97	0	32.12	11.06
Desmodus.rotundus	Pteronotus.davyi	0	85	147	28.19	2.84	1	23.52	7.8
Myotis.velifer	Tadarida.brasiliensis	0.5	108	163	28.13	2.31	1	2.42	23.9
Artibeus.jamaicensis	Carollia.brevicauda	0.4	44	150	27.98	2.94	0	26.9	1.7
Artibeus.jamaicensis	Carollia.perspicillata	0.2	44	127	27.45	3.29	1	22.5	7.9
Carollia.perspicillata	Trachops.cirrhosus	0.33	55.6	30	27.12	4.32	0	17.8	5.6
Artibeus.lituratus	Carollia.perspicillata	0.5	44	113	26.4	3.05	0	40.2	0.1
Artibeus.jamaicensis	Myotis.nigricans	0.25	120	103	26.06	3.67	0	37.36	3.93
Artibeus.lituratus	Sturnira.ludovici	0	34.2	91	25.91	3.56	1	38.4	12.1
Desmodus.rotundus	Myotis.nigricans	0	120	95	25.26	3.4	1	28.76	9.48
Carollia.brevicauda	Trachops.cirrhosus	0.25	55.6	32	25.25	4.25	0	22.2	16.56
Glossophaga.commissarisi	Pteronotus.parnellii	0	85	99	25.1	2.31	0	10.43	32.66
Lasiurus.cinereus	Tadarida.brasiliensis	0.5	108	104	25.09	2.28	1	14.6	11.73
Artibeus.lituratus	Myotis.nigricans	0	120	89	24.22	3.21	0	55.06	3.13
Lonchorhina.aurita	Trachops.cirrhosus	0	55.2	11	24.13	4.28	1	21.6	22.56
Desmodus.rotundus	Glossophaga.commissarisi	0	62	109	23.72	2.75	0	23.93	21.6
Carollia.brevicauda	Myotis.nigricans	0.33	120	51	23.26	2.99	0	10.46	3.22
Carollia.perspicillata	Pteronotus.parnellii	0.33	85	90	23.18	2.23	0	0.4	27.12
Carollia.brevicauda	Pteronotus.parnellii	0.25	85	104	23.15	2.15	0	19.5	16
Carollia.brevicauda	Desmodus.rotundus	0	62	123	22.83	1.97	0	18.3	4.92
Myotis.nigricans	Pteronotus.parnellii	0	120	75	22.59	2.31	1	15.26	9.32

A. Bats' characteristics of Known model

Myotis.nigricans	Sturnira.ludovici	0	120	35	22	3.08	0	16.66	6.1
Artibeus.jamaicensis	Sturnira.ludovici	0	34.2	84	21.49	3.07	0	20.7	6.23
Desmodus.rotundus	Sturnira.ludovici	0	62	79	21.32	2.98	0	12.1	5.9
Glossophaga.commissarisi	Sturnira.ludovici	1	53.4	40	21	2.93	0	11.83	8.76
Glossophaga.commissarisi	Trachops.cirrhosus	0.5	55.6	24	20.94	3.72	0	27.83	0.71
Pteronotus.parnellii	Sturnira.ludovici	0	85	65	20.56	2.81	0	1.4	0.8
Carollia.perspicillata	Lonchorhina.aurita	0.5	55.6	18	20.21	4.12	0	3.8	1.7
Glossophaga.commissarisi	Myotis.nigricans	0	120	40	20.2	2.83	0	4.83	3.52
Carollia.perspicillata	Desmodus.rotundus	0	62	95	20.04	1.92	0	13.9	6.9
Carollia.brevicauda	Pteronotus.davyi	0.33	85	61	19.65	2.35	0	5.22	17.7
Carollia.brevicauda	Sturnira.ludovici	0	44	41	19.04	2.74	1	6.2	17.32
Lasiurus.cinereus	Myotis.velifer	0	52	71	18.94	2.11	1	17.02	0.6
Nyctinomops.macrotis	Tadarida.brasiliensis	0	47.2	44	18.6	2.36	1	8.6	2.5
Glossophaga.commissarisi	Pteronotus.davyi	0	85	51	18.31	2.34	0	0.41	3.8
Lasiurus.cinereus	Nyctinomops.macrotis	0	108	27	18.01	3.1	1	6	27.42
Carollia.perspicillata	Pteronotus.davyi	0.5	85	49	18.01	2.34	0	9.62	11.32
Pteronotus.parnellii	Trachops.cirrhosus	0	85	34	18	3.71	1	17.4	4.2
Carollia.perspicillata	Myotis.nigricans	0	120	35	17.93	2.69	0	14.86	44
Carollia.brevicauda	Glossophaga.commissarisi	0	53.4	48	17.69	2.39	0	5.63	26.3
Carollia.brevicauda	Lonchorhina.aurita	0.33	55.6	18	17.57	3.85	0	0.6	20.8
Artibeus.lituratus	Trachops.cirrhosus	0	55.6	37	17.11	3.84	0	22.4	7.96
Artibeus.jamaicensis	Trachops.cirrhosus	0	55.6	40	17.05	4.21	0	4.7	8.7
Pteronotus.davyi	Sturnira.ludovici	0	85	38	16.99	2.58	0	11.42	27.83
Nyctinomops.laticaudatus	Pteronotus.parnellii	0.5	120	36	16.08	2.27	1	6.5	11.12
Artibeus.jamaicensis	Nyctinomops.laticaudatus	0.25	120	40	14.6	2.99	0	28.6	38.5
Desmodus.rotundus	Nyctinomops.laticaudatus	0	120	37	14.21	2.86	1	20	1.8
Carollia.perspicillata	Glossophaga.commissarisi	0.5	53.4	34	14.07	2.21	1	10.03	6
Glossophaga.commissarisi	Lonchorhina.aurita	0	55.6	13	13.99	3.38	0	6.23	5.54
Carollia.perspicillata	Sturnira.ludovici	0.5	44	27	13.93	2.43	0	1.8	11.02
Pteronotus.davyi	Trachops.cirrhosus	0	85	18	13.27	2.9	1	27.42	3.9
Desmodus.rotundus	Tadarida.brasiliensis	0.33	120	125	13.26	1.3	1	20.8	1.3
Desmodus.rotundus	Myotis.velifer	0	120	106	13.22	1.42	1	23.22	12.2
Myotis.nigricans	Pteronotus.davyi	0	120	31	12.9	2.08	1	5.24	17.8
Myotis.velifer	Nyctinomops.macrotis	0	108	27	12.78	2.5	1	11.02	14.8

A. Bats' characteristics of Known model

Lonchorhina.aurita	Pteronotus.parnellii	0.5	85	20	12.63	2.33	1	4.2	46.3
Desmodus.rotundus	Trachops.cirrhosus	0	62	28	12.01	2.75	1	3.9	5.24
Artibeus.lituratus	Nyctinomops.laticaudatus	1	120	31	11.93	2.48	0	46.3	22.2
Artibeus.lituratus	Lonchorhina.aurita	1	55.6	21	11.89	3.27	0	44	10.03
Myotis.nigricans	Nyctinomops.laticaudatus	0	108	13	11.45	2.71	1	8.76	0.3
Artibeus.jamaicensis	Lonchorhina.aurita	0.25	55.6	22	11.41	3.28	0	26.3	17.4
Sturnira.ludovici	Trachops.cirrhosus	0.5	55.6	11	11.4	2.87	0	16	14.86
Nyctinomops.macrotis	Pteronotus.davyi	0	120	19	10.99	1.85	1	11.32	16.66
Nyctinomops.macrotis	Pteronotus.parnellii	0	120	28	10.92	2.18	1	1.3	6.5
Pteronotus.parnellii	Tadarida.brasiliensis	0.33	120	88	10.51	1.18	1	7.3	22.4
Myotis.nigricans	Trachops.cirrhosus	0	120	10	9.89	2.68	1	32.66	20
Myotis.velifer	Pteronotus.parnellii	0.5	120	72	9.88	1.2	1	9.72	11.42
Carollia.perspicillata	Nyctinomops.laticaudatus	0.5	120	13	9.63	2.41	0	6.1	7.3
Carollia.brevicauda	Nyctinomops.laticaudatus	0.33	120	15	9.63	2.33	0	1.7	2.72
Desmodus.rotundus	Nyctinomops.macrotis	0	120	29	9.5	2	1	12.2	28.6
Nyctinomops.laticaudatus	Pteronotus.davyi	1	120	15	9.21	2.07	1	3.52	4.7
Desmodus.rotundus	Lonchorhina.aurita	0	62	17	8.98	2.59	1	17.7	4.83
Lonchorhina.aurita	Sturnira.ludovici	0	55.6	7	8.96	2.65	0	5.6	11.83
Artibeus.lituratus	Nyctinomops.macrotis	0	120	26	8.59	1.88	0	38.5	6.2
Desmodus.rotundus	Lasiurus.cinereus	0.5	120	53	8.37	1.26	1	6.2	8.6
Lonchorhina.aurita	Pteronotus.davyi	1	85	9	7.91	2.21	1	9.48	32.5
Myotis.nigricans	Myotis.velifer	0	21.8	27	7.88	1.45	1	5.54	5.63
Artibeus.lituratus	Myotis.velifer	1	120	73	7.72	0.98	0	49.52	9.62
Nyctinomops.laticaudatus	Trachops.cirrhosus	0	120	5	7.34	2.64	1	23.9	10.46
Myotis.velifer	Sturnira.ludovici	0	120	24	7.24	1.51	0	11.12	0.41
Artibeus.lituratus	Lasiurus.cinereus	0	120	46	7.07	1.13	0	32.5	6.2
Lasiurus.cinereus	Pteronotus.parnellii	0	120	39	6.95	1.11	1	7.3	5.22
Glossophaga.commissarisi	Nyctinomops.macrotis	1	120	11	6.93	1.97	0	11.73	1.4
Myotis.nigricans	Nyctinomops.macrotis	0	108	9	6.91	2.08	1	16.56	31.82
Artibeus.lituratus	Tadarida.brasiliensis	0.5	120	81	6.7	0.8	0	47.1	17.02
Myotis.velifer	Pteronotus.davyi	1	120	34	6.37	1.12	1	0.3	9.72
Lonchorhina.aurita	Myotis.nigricans	0	120	5	5.99	2.23	1	11.06	49.52
Artibeus.jamaicensis	Nyctinomops.macrotis	0	120	22	5.99	1.44	0	20.8	29.4
Pteronotus.davyi	Tadarida.brasiliensis	0.5	120	39	5.98	0.97	1	2.72	15.26

A. Bats' characteristics of Known model

Artibeus.jamaicensis	Myotis.velifer	0.25	120	71	5.9	0.77	0	31.82	12.1
Nyctinomops.laticaudatus	Nyctinomops.macrotis	0	20.8	5	5.83	2.22	1	7.8	47.1
Lonchorhina.aurita	Nyctinomops.laticaudatus	1	120	3	5.46	2.51	1	2.3	20.7
Nyctinomops.macrotis	Sturnira.ludovici	1	120	7	5.34	1.82	0	0.1	7.3
Sturnira.ludovici	Tadarida.brasiliensis	0	120	23	5.31	1.08	0	8.7	55.06
Glossophaga.commissarisi	Nyctinomops.laticaudatus	0	120	8	5.23	1.77	0	3.93	0.4
Lasiurus.cinereus	Sturnira.ludovici	0	120	13	5.12	1.39	0	5.9	38.4
Nyctinomops.laticaudatus	Tadarida.brasiliensis	0.5	47.2	14	5.11	1.29	1	0.8	13.9
Myotis.nigricans	Tadarida.brasiliensis	0	108	23	4.95	1.02	1	7.96	28.76
Nyctinomops.laticaudatus	Sturnira.ludovici	0	120	6	4.94	1.81	0	7.9	10.43
Myotis.velifer	Nyctinomops.laticaudatus	1	108	11	4.65	1.43	1	3.22	37.36
Artibeus.jamaicensis	Tadarida.brasiliensis	0.5	120	74	4.1	0.52	0	29.4	4.4
Lasiurus.cinereus	Pteronotus.davyi	0	120	17	3.94	0.95	1	17.32	19.5
Lasiurus.cinereus	Myotis.nigricans	0	52	11	3.76	1.13	1	22.56	14.6
Carollia.perspicillata	Nyctinomops.macrotis	0.5	120	6	3.31	1.33	0	1.7	23.22
Lasiurus.cinereus	Nyctinomops.laticaudatus	0	108	5	2.52	1.13	1	13.8	23.93
Artibeus.jamaicensis	Lasiurus.cinereus	0.25	120	31	2.4	0.47	0	14.8	40.2
Carollia.perspicillata	Tadarida.brasiliensis	0.33	120	17	1.64	0.4	0	6.9	18.3
Glossophaga.commissarisi	Myotis.velifer	0	120	14	1.51	0.41	0	0.71	20.8
Carollia.perspicillata	Myotis.velifer	0.5	120	13	1.35	0.38	0	9.32	22.5
Carollia.brevicauda	Nyctinomops.macrotis	0	120	4	1.22	0.62	0	6.1	50.23
Carollia.brevicauda	Lasiurus.cinereus	0	120	9	0.98	0.33	0	12.1	32.53
Nyctinomops.macrotis	Trachops.cirrhosus	0.5	120	1	0.8	0.8	1	16.1	44.6
Carollia.brevicauda	Tadarida.brasiliensis	0.25	120	18	0.8	0.19	0	2.5	23.52
Lonchorhina.aurita	Myotis.velifer	1	120	2	0.35	0.25	1	5.52	26.9
Lasiurus.cinereus	Lonchorhina.aurita	0	120	1	0.14	0.14	1	11.5	49.82
Carollia.brevicauda	Myotis.velifer	0.33	120	12	0.09	0.03	0	4.92	10.02
Lonchorhina.aurita	Tadarida.brasiliensis	0.5	120	2	0	0	1	3.1	32.12
Lasiurus.cinereus	Trachops.cirrhosus	0	120	1	-0.31	-0.31	1	10.1	2.42
Glossophaga.commissarisi	Tadarida.brasiliensis	0	120	11	-0.31	-0.09	0	3.13	39.8
Carollia.perspicillata	Lasiurus.cinereus	0	120	4	-0.47	-0.23	0	7.7	13.5
Lonchorhina.aurita	Nyctinomops.macrotis	0	120	0	-0.54	-4.08	1	5.5	22.1
Glossophaga.commissarisi	Lasiurus.cinereus	0	120	4	-0.56	-0.28	0	17.73	26.3
Tadarida.brasiliensis	Trachops.cirrhosus	0	120	2	-0.65	-0.46	1	24.7	8.6

A. Bats' characteristics of Known model

Myotis.velifer	Trachops.cirrhosus	0	120	11	-0.94	-0.91	1	27.12	17.7

B. Bats' community of Known model

	Bat.coronavirus	Bat.pegivirus	Dengue	Porcine.rubulavirus.PorPV	Rabia.	Hantavirus
Artibeus.jamaicensis	1	0	1	1	1	0
Artibeus.lituratus	1	0	0	0	0	0
Artibeus.phaeotis	1	0	0	0	0	0
Artibeus.watsoni	0	1	0	0	0	0
Carollia.brevicauda	1	0	1	0	0	1
Carollia.perspicillata	1	1	0	0	0	0
Desmodus.rotundus	0	0	0	1	1	0
Glossophaga.commissarisi	0	1	0	0	0	0
Lasiurus.cinereus	0	0	0	0	1	0
Lonchorhina.aurita	1	0	0	0	0	0
Myotis.nigricans	0	0	1	0	0	0
Myotis.velifer	1	0	0	0	0	0
Nyctinomops.laticaudatus	1	0	0	0	0	0
Nyctinomops.macrotis	0	1	0	0	0	0
Pteronotus.davyi	1	0	0	0	0	0
Pteronotus.parnellii	1	0	0	1	0	0
Sturnira.ludovici	0	1	0	0	0	0
Tadarida.brasiliensis	1	0	0	0	1	0
Trachops.cirrhosus	0	1	0	0	0	1
