# Supplementary material for: Niche theory‐based modeling of assembly processes of viral communities in bats
Source: Ecol Evol. 2021 Apr 3;11(11):6305–14. doi: 10.1002/ece3.7482 (PMC8207334; doi:10.1002/ece3.7482)
Supplement: Supplementary file 2 — Appendix S2 [file ECE3-11-6305-s002.pdf]

A. Bats' characteristics of Known model

A	B	Similarity	dist.phylo	spatial	epsilon	score	trophic.guild	body.mass	dif.B.M
Anoura geoffroyi	Antrozous pallidus	0.0000	120	6	-0.31	-0.13	0	18.6	7.2
Anoura geoffroyi	Artibeus jamaicensis	0.0000	53.4	147	24.97	1.98	1	28.3	26.6
Anoura geoffroyi	Artibeus lituratus	0.0000	53.4	152	28.95	2.23	0	37.15	44.3
Anoura geoffroyi	Carollia perspicillata	0.0000	53.4	36	12.42	1.99	1	17.05	4.1
Anoura geoffroyi	Carollia sowelli	0.0000	53.4	5	7.86	3.28	0	14.85	0.3
Anoura geoffroyi	Choeroniscus godmani	0.0000	29.8	16	13.57	3.17	0	11.45	7.1
Anoura geoffroyi	Corynorhinus townsendii	0.0000	120	11	2.66	0.81	0	12.65	4.7
Anoura geoffroyi	Dermanura phaeotis	0.0000	53.4	56	14.25	1.84	1	13.35	3.3
Anoura geoffroyi	Dermanura tolteca	0.0000	53.4	121	34.91	2.96	0	15.235	0.47
Anoura geoffroyi	Dermanura watsoni	0.0000	53.4	8	7.6	2.53	1	13.1	3.8
Anoura geoffroyi	Desmodus rotundus	0.0000	62	149	27.27	2.13	0	24	18
Anoura geoffroyi	Diphylla ecaudata	0.0000	62	23	11.76	2.32	0	21.55	13.1
Anoura geoffroyi	Eptesicus brasiliensis	0.0000	120	14	14.03	3.52	0	12.095	5.81
Anoura geoffroyi	Eptesicus furinalis	0.0000	120	26	12.24	2.28	0	11.335	7.33
Anoura geoffroyi	Eptesicus fuscus	0.0000	120	68	15.65	1.84	0	16.15	2.3
Anoura geoffroyi	Euderma maculatum	0.0000	120	0	-0.47	-3.81	0	15.6	1.2
Anoura geoffroyi	Eumops auripendulus	0.0000	120	2	4.97	3.28	0	21.7	13.4
Anoura geoffroyi	Eumops ferox	0.0000	120	6	4.48	1.78	0	25.45	20.9
Anoura geoffroyi	Eumops nanus	0.0000	120	2	3.24	2.19	0	13.55	2.9
Anoura geoffroyi	Eumops perotis	0.0000	120	3	2.25	1.29	0	32.95	35.9
Anoura geoffroyi	Glossophaga commissarisi	0.0000	38.8	59	21.33	2.61	1	12.035	5.93
Anoura geoffroyi	Glossophaga soricina	0.0000	38.8	138	24.09	1.97	1	12.45	5.1
Anoura geoffroyi	Lasionycteris noctivagans	0.0000	120	0	-0.27	-2.71	0	12.95	4.1
Anoura geoffroyi	Lasiurus blossevillii	0.0000	120	39	13.78	2.11	0	12	6
Anoura geoffroyi	Lasiurus borealis	0.0000	120	11	5.42	1.6	0	13.65	2.7
Anoura geoffroyi	Lasiurus cinereus	0.0000	120	36	11.11	1.8	0	20.9	11.8
Anoura geoffroyi	Lasiurus ega	0.0000	120	16	7.32	1.78	0	13.55	2.9
Anoura geoffroyi	Lasiurus intermedius	0.0000	120	30	13.7	2.37	0	18.95	7.9
Anoura geoffroyi	Lasiurus xanthinus	0.0000	120	13	4.13	1.15	0	15.5	1
Anoura geoffroyi	Lonchorhina aurita	0.0000	55.6	9	7.91	2.49	0	15.15	0.3
Anoura geoffroyi	Molossus molossus	0.0000	120	10	8.06	2.41	0	14.4	1.2
Anoura geoffroyi	Molossus rufus	0.0000	120	39	12.26	1.9	0	23.35	16.7
Anoura geoffroyi	Myotis albescens	0.0000	120	4	5.82	2.72	0	10.325	9.35

A. Bats' characteristics of Known model

Anoura geoffroyi	Myotis californicus	0.0000	120	30	8.49	1.53	0	9.695	10.61
Anoura geoffroyi	Myotis evotis	0.0000	120	0	-0.91	-5.11	0	10.93	8.14
Anoura geoffroyi	Myotis nigricans	0.0000	120	50	21.79	2.88	0	9.62	10.76
Anoura geoffroyi	Myotis occultus	0.0000	120	0	-0.34	-3.12	0	11.385	7.23
Anoura geoffroyi	Myotis velifer	0.0000	120	62	14.16	1.75	0	12.39	5.22
Anoura geoffroyi	Myotis volans	0.0000	120	6	3.56	1.44	0	11.82	6.36
Anoura geoffroyi	Myotis yumanensis	0.0000	120	13	3.48	0.97	0	10.075	9.85
Anoura geoffroyi	Nycticeius humeralis	0.0000	120	1	-0.45	-0.45	0	12.055	5.89
Anoura geoffroyi	Nyctinomops laticaudatus	0.0000	120	10	5.63	1.74	0	14	2
Anoura geoffroyi	Nyctinomops macrotis	0.0000	120	14	7.66	1.97	0	17.9	5.8
Anoura geoffroyi	Parastrellus hesperus	0.0000	120	11	1.41	0.43	0	9.28	11.44
Anoura geoffroyi	Phyllostomus discolor	0.0000	55.6	25	15.79	2.95	1	25.75	21.5
Anoura geoffroyi	Pteronotus davyi	0.0000	85	60	18.36	2.25	0	12.24	5.52
Anoura geoffroyi	Pteronotus parnellii	0.0000	85	111	23.78	2.16	0	17.25	4.5
Anoura geoffroyi	Sturnira hondurensis	0.0000	53.4	99	32.71	3.07	0	17.95	5.9
Anoura geoffroyi	Sturnira lilium	0.0000	53.4	87	27.02	2.71	1	17.6	5.2
Anoura geoffroyi	Tadarida brasiliensis	0.0000	120	51	8.85	1.24	0	13.6	2.8
Anoura geoffroyi	Trachops cirrhosus	0.0000	55.6	17	12.28	2.78	0	25.95	21.9
Anoura geoffroyi	Vampyrodes caraccioli	0.0000	53.4	8	8.46	2.8	0	25.4	20.8
Antrozous pallidus	Artibeus jamaicensis	0.2000	120	13	-1.58	-0.44	0	31.9	19.4
Antrozous pallidus	Artibeus lituratus	0.2000	120	14	-0.74	-0.2	0	40.75	37.1
Antrozous pallidus	Carollia perspicillata	0.0000	120	0	-2.24	-6.91	0	20.65	3.1
Antrozous pallidus	Carollia sowelli	0.0000	120	0	-0.55	-4.09	0	18.45	7.5
Antrozous pallidus	Choeroniscus godmani	0.0000	120	0	-1.01	-5.32	0	15.05	14.3
Antrozous pallidus	Corynorhinus townsendii	1.0000	52	38	17	2.51	1	16.25	11.9
Antrozous pallidus	Dermanura phaeotis	0.0000	120	0	-2.96	-7.47	0	16.95	10.5
Antrozous pallidus	Dermanura tolteca	0.0000	120	12	1.28	0.37	0	18.835	6.73
Antrozous pallidus	Dermanura watsoni	0.0000	120	0	-0.86	-5.01	0	16.7	11
Antrozous pallidus	Desmodus rotundus	0.1250	120	26	1.92	0.38	1	27.6	10.8
Antrozous pallidus	Diphylla ecaudata	0.5000	120	5	1.69	0.76	1	25.15	5.9
Antrozous pallidus	Eptesicus brasiliensis	1.0000	52	0	-0.86	-5.01	1	15.695	13.01
Antrozous pallidus	Eptesicus furinalis	1.0000	52	3	0.11	0.07	1	14.935	14.53
Antrozous pallidus	Eptesicus fuscus	0.1667	52	70	18.83	2.1	1	19.75	4.9
Antrozous pallidus	Euderma maculatum	1.0000	52	4	9.3	4.2	1	19.2	6

A. Bats' characteristics of Known model

Antrozous pallidus	Eumops auripendulus	1.0000	108	0	-0.35	-3.18	1	25.3	6.2
Antrozous pallidus	Eumops ferox	0.5000	108	0	-0.99	-5.29	1	29.05	13.7
Antrozous pallidus	Eumops nanus	1.0000	108	0	0.49	-3.87	1	17.15	10.1
Antrozous pallidus	Eumops perotis	1.0000	108	11	12.24	3.27	1	36.55	28.7
Antrozous pallidus	Glossophaga commissarisi	0.0000	120	0	-2.29	-6.95	0	15.635	13.13
Antrozous pallidus	Glossophaga soricina	0.2000	120	17	-0.43	-0.11	0	16.05	12.3
Antrozous pallidus	Lasionycteris noctivagans	1.0000	52	1	3.97	3.51	1	16.55	11.3
Antrozous pallidus	Lasiurus blossevillii	1.0000	52	32	12.61	2.08	1	15.6	13.2
Antrozous pallidus	Lasiurus borealis	1.0000	52	11	6.38	1.83	1	17.25	9.9
Antrozous pallidus	Lasiurus cinereus	1.0000	52	39	14.22	2.12	1	24.5	4.6
Antrozous pallidus	Lasiurus ega	1.0000	52	11	5.36	1.56	1	17.15	10.1
Antrozous pallidus	Lasiurus intermedius	1.0000	52	9	3.46	1.14	1	22.55	0.7
Antrozous pallidus	Lasiurus xanthinus	1.0000	52	31	14.73	2.42	1	19.1	6.2
Antrozous pallidus	Lonchorhina aurita	0.0000	120	0	-0.93	-5.16	1	18.75	6.9
Antrozous pallidus	Molossus molossus	0.3333	108	0	-1.01	-5.32	1	18	8.4
Antrozous pallidus	Molossus rufus	0.5000	108	2	-1.56	-1.06	1	26.95	9.5
Antrozous pallidus	Myotis albescens	1.0000	52	0	-0.57	-4.19	1	13.925	16.55
Antrozous pallidus	Myotis californicus	1.0000	52	58	21.71	2.59	1	13.295	17.81
Antrozous pallidus	Myotis evotis	1.0000	52	10	11.88	3.33	1	14.53	15.34
Antrozous pallidus	Myotis nigricans	0.3333	52	6	1.26	0.52	1	13.22	17.96
Antrozous pallidus	Myotis occultus	0.3333	52	1	3.14	2.82	1	14.985	14.43
Antrozous pallidus	Myotis velifer	0.3333	52	48	12.07	1.67	1	15.99	12.42
Antrozous pallidus	Myotis volans	0.0000	52	19	15.91	3.24	1	15.42	13.56
Antrozous pallidus	Myotis yumanensis	0.5000	52	31	13.31	2.22	1	13.675	17.05
Antrozous pallidus	Nycticeius humeralis	1.0000	52	12	9.76	2.56	1	15.655	13.09
Antrozous pallidus	Nyctinomops laticaudatus	0.5000	108	2	0.27	0.2	1	17.6	9.2
Antrozous pallidus	Nyctinomops macrotis	0.5000	108	19	12.52	2.6	1	21.5	1.4
Antrozous pallidus	Parastrellus hesperus	1.0000	52	63	24.01	2.73	1	12.88	18.64
Antrozous pallidus	Phyllostomus discolor	0.0000	120	0	-1.34	-5.88	0	29.35	14.3
Antrozous pallidus	Pteronotus davyi	0.0000	120	7	0.15	0.06	1	15.84	12.72
Antrozous pallidus	Pteronotus parnellii	0.0000	120	14	0.23	0.06	1	20.85	2.7
Antrozous pallidus	Sturnira hondurensis	0.0000	120	10	1.42	0.46	0	21.55	1.3
Antrozous pallidus	Sturnira lilium	0.0000	120	5	-0.77	-0.34	0	21.2	2
Antrozous pallidus	Tadarida brasiliensis	0.1429	108	88	21.16	2.11	1	17.2	10

A. Bats' characteristics of Known model

Antrozous pallidus	Trachops cirrhosus	0.0000	120	0	-1.16	-5.6	1	29.55	14.7
Antrozous pallidus	Vampyrodes caraccioli	0.0000	120	0	-0.79	-4.83	0	29	13.6
Artibeus jamaicensis	Artibeus lituratus	0.4286	7.6	438	50.4	3.22	0	50.45	17.7
Artibeus jamaicensis	Carollia perspicillata	0.0909	44	127	27.45	3.29	1	30.35	22.5
Artibeus jamaicensis	Carollia sowelli	0.0000	44	10	9.18	9.73	0	28.15	26.9
Artibeus jamaicensis	Choeroniscus godmani	0.0000	53.4	32	15.81	4.9	0	24.75	33.7
Artibeus jamaicensis	Corynorhinus townsendii	0.2000	120	14	-0.26	-0.07	0	25.95	31.3
Artibeus jamaicensis	Dermanura phaeotis	0.0000	16.6	231	38	3.46	1	26.65	29.9
Artibeus jamaicensis	Dermanura tolteca	0.0000	16.6	214	35.09	3.19	0	28.535	26.13
Artibeus jamaicensis	Dermanura watsoni	0.0000	16.6	22	12.56	4.12	1	26.4	30.4
Artibeus jamaicensis	Desmodus rotundus	0.1818	62	387	41.93	2.64	0	37.3	8.6
Artibeus jamaicensis	Diphylla ecaudata	0.1667	62	57	17.17	2.92	0	34.85	13.5
Artibeus jamaicensis	Eptesicus brasiliensis	0.2000	120	19	10.61	3.28	0	25.395	32.41
Artibeus jamaicensis	Eptesicus furinalis	0.2000	120	73	20.67	3.24	0	24.635	33.93
Artibeus jamaicensis	Eptesicus fuscus	0.1000	120	89	8.81	1.03	0	29.45	24.3
Artibeus jamaicensis	Euderma maculatum	0.2000	120	1	0.48	0.53	0	28.9	25.4
Artibeus jamaicensis	Eumops auripendulus	0.2000	120	3	4.18	3.23	0	35	13.2
Artibeus jamaicensis	Eumops ferox	0.1667	120	29	14.41	4.11	0	38.75	5.7
Artibeus jamaicensis	Eumops nanus	0.2000	120	7	7.06	4.07	0	26.85	29.5
Artibeus jamaicensis	Eumops perotis	0.2000	120	5	1.52	0.75	0	46.25	9.3
Artibeus jamaicensis	Glossophaga commissarisi	0.0000	53.4	144	30.78	3.67	1	25.335	32.53
Artibeus jamaicensis	Glossophaga soricina	0.2500	53.4	439	47.17	2.87	1	25.75	31.7
Artibeus jamaicensis	Lasionycteris noctivagans	0.2000	120	1	1.81	2.13	0	26.25	30.7
Artibeus jamaicensis	Lasiurus blossevillii	0.2000	120	69	13.01	1.8	0	25.3	32.6
Artibeus jamaicensis	Lasiurus borealis	0.2000	120	21	5.27	1.28	0	26.95	29.3
Artibeus jamaicensis	Lasiurus cinereus	0.2000	120	31	2.4	0.47	0	34.2	14.8
Artibeus jamaicensis	Lasiurus ega	0.2000	120	50	14.07	2.41	0	26.85	29.5
Artibeus jamaicensis	Lasiurus intermedius	0.2000	120	65	17.06	2.62	0	32.25	18.7
Artibeus jamaicensis	Lasiurus xanthinus	0.2000	120	40	7.89	1.4	0	28.8	25.6
Artibeus jamaicensis	Lonchorhina aurita	0.0000	55.6	22	11.41	3.28	0	28.45	26.3
Artibeus jamaicensis	Molossus molossus	0.1429	120	27	13.02	3.48	0	27.7	27.8
Artibeus jamaicensis	Molossus rufus	0.1667	120	143	28.44	3.14	0	36.65	9.9
Artibeus jamaicensis	Myotis albescens	0.2000	120	8	6.69	3.11	0	23.625	35.95
Artibeus jamaicensis	Myotis californicus	0.2000	120	36	3.26	0.59	0	22.995	37.21

A. Bats' characteristics of Known model

Artibeus jamaicensis	Myotis evotis	0.2000	120	0	-1.62	-6.26	0	24.23	34.74
Artibeus jamaicensis	Myotis nigricans	0.3333	120	103	26.06	3.67	0	22.92	37.36
Artibeus jamaicensis	Myotis occultus	0.1429	120	0	-0.6	-4.27	0	24.685	33.83
Artibeus jamaicensis	Myotis velifer	0.1429	120	71	5.9	0.77	0	25.69	31.82
Artibeus jamaicensis	Myotis volans	0.0000	120	4	-0.33	-0.17	0	25.12	32.96
Artibeus jamaicensis	Myotis yumanensis	0.1667	120	26	2.9	0.62	0	23.375	36.45
Artibeus jamaicensis	Nycticeius humeralis	0.2000	120	3	-0.77	0.46	0	25.355	32.49
Artibeus jamaicensis	Nyctinomops laticaudatus	0.1667	120	40	14.6	2.99	0	27.3	28.6
Artibeus jamaicensis	Nyctinomops macrotis	0.1667	120	22	5.99	1.44	0	31.2	20.8
Artibeus jamaicensis	Parastrellus hesperus	0.2000	120	20	-0.31	-0.07	0	22.58	38.04
Artibeus jamaicensis	Phyllostomus discolor	0.0000	55.6	55	20.38	4.53	1	39.05	5.1
Artibeus jamaicensis	Pteronotus davyi	0.0000	85	159	28.98	2.97	0	25.54	32.12
Artibeus jamaicensis	Pteronotus parnellii	0.1000	85	315	40.66	2.95	0	30.55	22.1
Artibeus jamaicensis	Sturnira hondurensis	0.0000	34.2	138	24.94	2.63	0	31.25	20.7
Artibeus jamaicensis	Sturnira lilium	0.0000	34.2	184	33.13	3.3	1	30.9	21.4
Artibeus jamaicensis	Tadarida brasiliensis	0.0909	120	74	4.1	0.52	0	26.9	29.4
Artibeus jamaicensis	Trachops cirrhosus	0.0000	55.6	40	17.05	4.21	0	39.25	4.7
Artibeus jamaicensis	Vampyrodes caraccioli	0.0000	26.2	18	11.17	3.92	0	38.7	5.8
Artibeus lituratus	Carollia perspicillata	0.0909	44	113	26.4	3.05	0	39.2	40.2
Artibeus lituratus	Carollia sowelli	0.0000	44	9	8.92	4.5	1	37	44.6
Artibeus lituratus	Choeroniscus godmani	0.0000	53.4	27	14.3	3.66	1	33.6	51.4
Artibeus lituratus	Corynorhinus townsendii	0.2000	120	20	2.12	0.51	0	34.8	49
Artibeus lituratus	Dermanura phaeotis	0.0000	16.6	215	38.46	3.33	0	35.5	47.6
Artibeus lituratus	Dermanura tolteca	0.0000	16.6	204	36.54	3.19	1	37.385	43.83
Artibeus lituratus	Dermanura watsoni	0.0000	16.6	22	13.76	4.3	0	35.25	48.1
Artibeus lituratus	Desmodus rotundus	0.0833	62	356	42.02	2.61	0	46.15	26.3
Artibeus lituratus	Diphylla ecaudata	0.1667	62	56	18.55	3.04	0	43.7	31.2
Artibeus lituratus	Eptesicus brasiliensis	0.2000	120	21	13.06	3.96	0	34.245	50.11
Artibeus lituratus	Eptesicus furinalis	0.2000	120	58	17.42	2.7	0	33.485	51.63
Artibeus lituratus	Eptesicus fuscus	0.1000	120	91	10.86	1.24	0	38.3	42
Artibeus lituratus	Euderma maculatum	0.2000	120	2	2.07	1.62	0	37.75	43.1
Artibeus lituratus	Eumops auripendulus	0.2000	120	2	2.85	2.31	0	43.85	30.9
Artibeus lituratus	Eumops ferox	0.1667	120	22	11.53	3	0	47.6	23.4
Artibeus lituratus	Eumops nanus	0.2000	120	4	4.04	2.31	0	35.7	47.2

A. Bats' characteristics of Known model

Artibeus lituratus	Eumops perotis	0.2000	120	6	2.6	1.16	0	55.1	8.4
Artibeus lituratus	Glossophaga commissarisi	0.0000	53.4	137	31.92	3.59	0	34.185	50.23
Artibeus lituratus	Glossophaga soricina	0.2500	53.4	394	45.86	2.74	0	34.6	49.4
Artibeus lituratus	Lasionycteris noctivagans	0.2000	120	1	2.02	2.31	0	35.1	48.4
Artibeus lituratus	Lasiurus blossevillii	0.2000	120	74	16.02	2.1	0	34.15	50.3
Artibeus lituratus	Lasiurus borealis	0.2000	120	27	8.61	1.84	0	35.8	47
Artibeus lituratus	Lasiurus cinereus	0.2000	120	46	7.07	1.13	0	43.05	32.5
Artibeus lituratus	Lasiurus ega	0.2000	120	44	13.39	2.31	0	35.7	47.2
Artibeus lituratus	Lasiurus intermedius	0.2000	120	70	20.57	3	0	41.1	36.4
Artibeus lituratus	Lasiurus xanthinus	0.2000	120	38	8.44	1.5	0	37.65	43.3
Artibeus lituratus	Lonchorhina aurita	0.0000	55.6	21	11.89	3.27	0	37.3	44
Artibeus lituratus	Molossus molossus	0.1429	120	22	11.31	2.91	0	36.55	45.5
Artibeus lituratus	Molossus rufus	0.1667	120	130	28.04	3	0	45.5	27.6
Artibeus lituratus	Myotis albescens	0.2000	120	8	7.36	3.29	0	32.475	53.65
Artibeus lituratus	Myotis californicus	0.2000	120	40	5.25	0.9	0	31.845	54.91
Artibeus lituratus	Myotis evotis	0.2000	120	0	-1.48	-6.08	0	33.08	52.44
Artibeus lituratus	Myotis nigricans	0.3333	120	89	24.22	3.21	0	31.77	55.06
Artibeus lituratus	Myotis occultus	0.1429	120	1	1.47	1.62	0	33.535	51.53
Artibeus lituratus	Myotis velifer	0.1429	120	73	7.72	0.98	0	34.54	49.52
Artibeus lituratus	Myotis volans	0.0000	120	3	-0.52	-0.31	0	33.97	50.66
Artibeus lituratus	Myotis yumanensis	0.1667	120	22	2.6	0.59	0	32.225	54.15
Artibeus lituratus	Nycticeius humeralis	0.2000	120	4	0.06	0.03	0	34.205	50.19
Artibeus lituratus	Nyctinomops laticaudatus	0.1667	120	31	11.93	2.48	0	36.15	46.3
Artibeus lituratus	Nyctinomops macrotis	0.1667	120	26	8.59	1.88	0	40.05	38.5
Artibeus lituratus	Parastrellus hesperus	0.2000	120	21	0.69	0.16	0	31.43	55.74
Artibeus lituratus	Phyllostomus discolor	0.0000	55.6	47	18.7	3.59	0	47.9	22.8
Artibeus lituratus	Pteronotus davyi	0.0000	85	152	30.32	3	0	34.39	49.82
Artibeus lituratus	Pteronotus parnellii	0.0000	85	293	41.2	2.91	0	39.4	39.8
Artibeus lituratus	Sturnira hondurensis	0.0000	34.2	153	31.09	3.1	1	40.1	38.4
Artibeus lituratus	Sturnira lilium	0.0000	34.2	179	35.29	3.37	0	39.75	39.1
Artibeus lituratus	Tadarida brasiliensis	0.0909	120	81	6.7	0.8	0	35.75	47.1
Artibeus lituratus	Trachops cirrhosus	0.0000	55.6	37	17.11	3.84	0	48.1	22.4
Artibeus lituratus	Vampyrodes caraccioli	0.0000	26.2	14	9.2	3	1	47.55	23.5
Carollia perspicillata	Carollia sowelli	0.1250	11.2	8	15.44	5.02	0	16.9	4.4

A. Bats' characteristics of Known model

Carollia perspicillata	Choeroniscus godmani	0.1429	53.4	14	14.2	3.27	0	13.5	11.2
Carollia perspicillata	Corynorhinus townsendii	0.0000	120	1	-1.4	-1.31	0	14.7	8.8
Carollia perspicillata	Dermanura phaeotis	0.0000	44	86	28.96	2.76	1	15.4	7.4
Carollia perspicillata	Dermanura tolteca	0.1429	44	52	16.57	2.12	0	17.285	3.63
Carollia perspicillata	Dermanura watsoni	0.1429	44	17	20.63	4.38	1	15.15	7.9
Carollia perspicillata	Desmodus rotundus	0.1538	62	95	20.04	1.92	0	26.05	13.9
Carollia perspicillata	Diphylla ecaudata	0.0000	62	34	22.06	3.27	0	23.6	9
Carollia perspicillata	Eptesicus brasiliensis	0.0000	120	6	6.76	2.48	0	14.145	9.91
Carollia perspicillata	Eptesicus furinalis	0.0000	120	26	15.05	2.63	0	13.385	11.43
Carollia perspicillata	Eptesicus fuscus	0.0000	120	17	2.61	0.64	0	18.2	1.8
Carollia perspicillata	Euderma maculatum	0.0000	120	0	-0.4	-3.46	0	17.65	2.9
Carollia perspicillata	Eumops auripendulus	0.0000	120	2	5.98	3.63	0	23.75	9.3
Carollia perspicillata	Eumops ferox	0.0000	120	6	5.65	2.13	0	27.5	16.8
Carollia perspicillata	Eumops nanus	0.0000	120	1	1.77	1.69	0	15.6	7
Carollia perspicillata	Eumops perotis	0.0000	120	0	-0.81	-4.89	0	35	31.8
Carollia perspicillata	Glossophaga commissarisi	0.1429	53.4	34	14.06	2.21	1	14.085	10.03
Carollia perspicillata	Glossophaga soricina	0.2000	53.4	114	24.08	2.09	1	14.5	9.2
Carollia perspicillata	Lasionycteris noctivagans	0.0000	120	0	-0.23	-2.36	0	15	8.2
Carollia perspicillata	Lasiurus blossevillii	0.0000	120	9	2.33	0.78	0	14.05	10.1
Carollia perspicillata	Lasiurus borealis	0.0000	120	2	0.15	0.11	0	15.7	6.8
Carollia perspicillata	Lasiurus cinereus	0.0000	120	4	-0.47	-0.23	0	22.95	7.7
Carollia perspicillata	Lasiurus ega	0.0000	120	17	9.9	2.2	0	15.6	7
Carollia perspicillata	Lasiurus intermedius	0.0000	120	12	5.72	1.58	0	21	3.8
Carollia perspicillata	Lasiurus xanthinus	0.0000	120	6	1.61	0.66	0	17.55	3.1
Carollia perspicillata	Lonchorhina aurita	0.0000	55.6	18	20.21	4.12	0	17.2	3.8
Carollia perspicillata	Molossus molossus	0.0000	120	5	4.46	1.87	0	16.45	5.3
Carollia perspicillata	Molossus rufus	0.0000	120	49	19.86	2.54	0	25.4	12.6
Carollia perspicillata	Myotis albescens	0.0000	120	4	7.07	3.07	0	12.375	13.45
Carollia perspicillata	Myotis californicus	0.0000	120	5	-0.12	-0.05	0	11.745	14.71
Carollia perspicillata	Myotis evotis	0.0000	120	0	-0.76	-4.76	0	12.98	12.24
Carollia perspicillata	Myotis nigricans	0.1111	120	35	17.93	2.69	0	11.67	14.86
Carollia perspicillata	Myotis occultus	0.0000	120	0	-0.28	-2.77	0	13.435	11.33
Carollia perspicillata	Myotis velifer	0.0000	120	13	1.35	0.38	0	14.44	9.32
Carollia perspicillata	Myotis volans	0.0000	120	2	0.82	0.59	0	13.87	10.46

A. Bats' characteristics of Known model

Carollia perspicillata	Myotis yumanensis	0.0000	120	1	-1.43	-1.33	0	12.125	13.95
Carollia perspicillata	Nycticeius humeralis	0.0000	120	1	-0.11	-0.1	0	14.105	9.99
Carollia perspicillata	Nyctinomops laticaudatus	0.0000	120	13	9.63	2.41	0	16.05	6.1
Carollia perspicillata	Nyctinomops macrotis	0.1250	120	6	3.34	1.33	0	19.95	1.7
Carollia perspicillata	Parastrellus hesperus	0.0000	120	0	-2.31	-6.97	0	11.33	15.54
Carollia perspicillata	Phyllostomus discolor	0.0000	55.6	26	19.91	3.36	1	27.8	17.4
Carollia perspicillata	Pteronotus davyi	0.0000	85	49	18.01	2.34	0	14.29	9.62
Carollia perspicillata	Pteronotus parnellii	0.0833	85	90	23.18	2.23	0	19.3	0.4
Carollia perspicillata	Sturnira hondurensis	0.1429	44	37	13.24	2.02	0	20	1.8
Carollia perspicillata	Sturnira lilium	0.0909	44	73	27.13	2.8	1	19.65	1.1
Carollia perspicillata	Tadarida brasiliensis	0.0000	120	17	1.64	0.4	0	15.65	6.9
Carollia perspicillata	Trachops cirrhosus	0.1250	55.6	30	27.12	4.32	0	28	17.8
Carollia perspicillata	Vampyrodes caraccioli	0.0000	44	11	14.4	3.73	0	27.45	16.7
Carollia sowelli	Choeroniscus godmani	0.0000	53.4	5	21.6	4.71	1	11.3	6.8
Carollia sowelli	Corynorhinus townsendii	0.0000	120	0	-0.47	-3.78	0	12.5	4.4
Carollia sowelli	Dermanura phaeotis	0.0000	44	6	8.27	2.61	0	13.2	3
Carollia sowelli	Dermanura tolteca	0.0000	44	6	8.33	2.62	1	15.085	0.77
Carollia sowelli	Dermanura watsoni	0.0000	44	5	25.27	5.08	0	12.95	3.5
Carollia sowelli	Desmodus rotundus	0.0000	62	8	7.21	2.14	0	23.85	18.3
Carollia sowelli	Diphylla ecaudata	0.3333	62	2	5.23	2.77	0	21.4	13.4
Carollia sowelli	Eptesicus brasiliensis	0.0000	120	2	9.99	4.03	0	11.945	5.51
Carollia sowelli	Eptesicus furinalis	0.0000	120	1	2.2	1.91	0	11.185	7.03
Carollia sowelli	Eptesicus fuscus	0.0000	120	2	1.96	1.3	0	16	2.6
Carollia sowelli	Euderma maculatum	0.0000	120	0	-0.1	-0.62	0	15.45	1.5
Carollia sowelli	Eumops auripendulus	0.0000	120	2	25.38	6.47	0	21.55	13.7
Carollia sowelli	Eumops ferox	0.0000	120	3	13.07	4.17	0	25.3	21.2
Carollia sowelli	Eumops nanus	0.0000	120	1	8.89	4.53	0	13.4	2.6
Carollia sowelli	Eumops perotis	0.0000	120	0	-0.2	-2.05	0	32.8	36.2
Carollia sowelli	Glossophaga commissarisi	0.0000	53.4	3	5.25	2.42	0	11.885	5.63
Carollia sowelli	Glossophaga soricina	0.0000	53.4	9	7.99	2.21	0	12.3	4.8
Carollia sowelli	Lasionycteris noctivagans	0.0000	120	0	-0.06	0.48	0	12.8	3.8
Carollia sowelli	Lasiurus blossevillii	0.0000	120	0	-0.51	-3.93	0	11.85	5.7
Carollia sowelli	Lasiurus borealis	0.0000	120	0	-0.33	-3.08	0	13.5	2.4
Carollia sowelli	Lasiurus cinereus	0.0000	120	0	-0.55	-4.1	0	20.75	12.1

A. Bats' characteristics of Known model

Carollia sowelli	Lasiurus ega	0.0000	120	2	5.06	2.71	0	13.4	2.6
Carollia sowelli	Lasiurus intermedius	0.0000	120	0	-0.4	-3.48	0	18.8	8.2
Carollia sowelli	Lasiurus xanthinus	0.0000	120	0	-0.44	-3.64	0	15.35	1.3
Carollia sowelli	Lonchorhina aurita	0.0000	55.6	4	18.7	4.64	0	15	0.6
Carollia sowelli	Molossus molossus	0.0000	120	1	4.14	2.98	0	14.25	0.9
Carollia sowelli	Molossus rufus	0.0000	120	4	6.74	2.61	0	23.2	17
Carollia sowelli	Myotis albescens	0.0000	120	0	-0.13	-1.23	0	10.175	9.05
Carollia sowelli	Myotis californicus	0.0000	120	0	-0.56	-4.15	0	9.545	10.31
Carollia sowelli	Myotis evotis	0.0000	120	0	-0.18	-1.92	0	10.78	7.84
Carollia sowelli	Myotis nigricans	0.0000	120	4	8.67	3.06	0	9.47	10.46
Carollia sowelli	Myotis occultus	0.0000	120	0	-0.07	0.07	0	11.235	6.93
Carollia sowelli	Myotis velifer	0.0000	120	1	0.63	0.62	0	12.24	4.92
Carollia sowelli	Myotis volans	0.0000	120	1	3.58	2.71	0	11.67	6.06
Carollia sowelli	Myotis yumanensis	0.0000	120	0	-0.47	-3.8	0	9.925	9.55
Carollia sowelli	Nycticeius humeralis	0.0000	120	0	-0.26	-2.59	0	11.905	5.59
Carollia sowelli	Nyctinomops laticaudatus	0.0000	120	0	-0.3	-2.87	0	13.85	1.7
Carollia sowelli	Nyctinomops macrotis	0.0000	120	1	2.81	2.3	0	17.75	6.1
Carollia sowelli	Parastrellus hesperus	0.0000	120	0	-0.56	-4.13	0	9.13	11.14
Carollia sowelli	Phyllostomus discolor	0.0000	55.6	4	12.84	3.83	0	25.6	21.8
Carollia sowelli	Pteronotus davyi	0.0000	85	6	9.52	2.86	0	12.09	5.22
Carollia sowelli	Pteronotus parnellii	0.0000	85	8	8.72	2.45	0	17.1	4.8
Carollia sowelli	Sturnira hondurensis	0.0000	44	5	7.96	2.7	1	17.8	6.2
Carollia sowelli	Sturnira lilium	0.0000	44	8	12.51	3.1	0	17.45	5.5
Carollia sowelli	Tadarida brasiliensis	0.0000	120	1	0.37	0.38	0	13.45	2.5
Carollia sowelli	Trachops cirrhosus	0.3333	55.6	4	14.92	4.14	0	25.8	22.2
Carollia sowelli	Vampyrodes caraccioli	0.0000	44	1	5.38	3.48	1	25.25	21.1
Choeroniscus godmani	Corynorhinus townsendii	0.0000	120	0	-0.86	-5	0	9.1	2.4
Choeroniscus godmani	Dermanura phaeotis	0.0000	53.4	23	17.38	2.78	0	9.8	3.8
Choeroniscus godmani	Dermanura tolteca	1.0000	53.4	18	13.44	2.54	1	11.685	7.57
Choeroniscus godmani	Dermanura watsoni	1.0000	53.4	5	13.47	3.86	0	9.55	3.3
Choeroniscus godmani	Desmodus rotundus	0.1250	62	23	10.98	1.99	0	20.45	25.1
Choeroniscus godmani	Diphylla ecaudata	0.0000	62	3	3.89	1.96	0	18	20.2
Choeroniscus godmani	Eptesicus brasiliensis	0.0000	120	1	2.4	2.07	0	8.545	1.29
Choeroniscus godmani	Eptesicus furinalis	0.0000	120	5	6.31	2.33	0	7.785	0.23

A. Bats' characteristics of Known model

Choeroniscus godmani	Eptesicus fuscus	0.0000	120	5	2.3	0.99	0	12.6	9.4
Choeroniscus godmani	Euderma maculatum	0.0000	120	0	-0.18	-1.85	0	12.05	8.3
Choeroniscus godmani	Eumops auripendulus	0.0000	120	2	13.69	5.24	0	18.15	20.5
Choeroniscus godmani	Eumops ferox	0.0000	120	2	4.4	2.5	0	21.9	28
Choeroniscus godmani	Eumops nanus	0.0000	120	1	4.69	3.3	0	10	4.2
Choeroniscus godmani	Eumops perotis	0.0000	120	0	-0.36	-3.27	0	29.4	43
Choeroniscus godmani	Glossophaga commissarisi	1.0000	38.8	19	18.91	3.14	0	8.485	1.17
Choeroniscus godmani	Glossophaga soricina	0.0000	38.8	31	14.98	2.25	0	8.9	2
Choeroniscus godmani	Lasionycteris noctivagans	0.0000	120	0	-0.1	-0.75	0	9.4	3
Choeroniscus godmani	Lasiurus blossevillii	0.0000	120	4	3.37	1.55	0	8.45	1.1
Choeroniscus godmani	Lasiurus borealis	0.0000	120	0	-0.61	-4.3	0	10.1	4.4
Choeroniscus godmani	Lasiurus cinereus	0.0000	120	1	-0.02	-0.02	0	17.35	18.9
Choeroniscus godmani	Lasiurus ega	0.0000	120	10	14.07	3.19	0	10	4.2
Choeroniscus godmani	Lasiurus intermedius	0.0000	120	6	7.36	2.44	0	15.4	15
Choeroniscus godmani	Lasiurus xanthinus	0.0000	120	5	5.43	2.08	0	11.95	8.1
Choeroniscus godmani	Lonchorhina aurita	0.0000	55.6	4	9.88	3.41	0	11.6	7.4
Choeroniscus godmani	Molossus molossus	0.0000	120	4	9.07	3.23	0	10.85	5.9
Choeroniscus godmani	Molossus rufus	0.0000	120	14	12.85	2.68	0	19.8	23.8
Choeroniscus godmani	Myotis albescens	0.0000	120	0	-0.24	-2.45	0	6.775	2.25
Choeroniscus godmani	Myotis californicus	0.0000	120	0	-1.04	-5.37	0	6.145	3.51
Choeroniscus godmani	Myotis evotis	0.0000	120	0	-0.34	-3.15	0	7.38	1.04
Choeroniscus godmani	Myotis nigricans	0.0000	120	10	11.56	2.8	0	6.07	3.66
Choeroniscus godmani	Myotis occultus	0.0000	120	0	-0.13	-1.16	0	7.835	0.13
Choeroniscus godmani	Myotis velifer	0.0000	120	3	0.86	0.5	0	8.84	1.88
Choeroniscus godmani	Myotis volans	0.0000	120	1	1.6	1.49	0	8.27	0.74
Choeroniscus godmani	Myotis yumanensis	0.0000	120	0	-0.87	-5.02	0	6.525	2.75
Choeroniscus godmani	Nycticeius humeralis	0.0000	120	0	-0.48	-3.82	0	8.505	1.21
Choeroniscus godmani	Nyctinomops laticaudatus	0.0000	120	1	1.28	1.22	0	10.45	5.1
Choeroniscus godmani	Nyctinomops macrotis	0.5000	120	1	1.11	1.07	0	14.35	12.9
Choeroniscus godmani	Parastrellus hesperus	0.0000	120	0	-1.03	-5.36	0	5.73	4.34
Choeroniscus godmani	Phyllostomus discolor	0.0000	55.6	14	24.44	4.05	0	22.2	28.6
Choeroniscus godmani	Pteronotus davyi	0.0000	85	11	8.98	2.26	0	8.69	1.58
Choeroniscus godmani	Pteronotus parnellii	0.0000	85	25	14.68	2.4	0	13.7	11.6
Choeroniscus godmani	Sturnira hondurensis	1.0000	53.4	15	12.84	2.62	1	14.4	13

A. Bats' characteristics of Known model

Choeroniscus godmani	Sturnira lilium	0.2000	53.4	20	16.69	2.84	0	14.05	12.3
Choeroniscus godmani	Tadarida brasiliensis	0.0000	120	2	-0.22	-0.16	0	10.05	4.3
Choeroniscus godmani	Trachops cirrhosus	0.5000	55.6	9	18.07	3.86	0	22.4	29
Choeroniscus godmani	Vampyrodes caraccioli	0.0000	53.4	4	11.74	3.8	1	21.85	27.9
Corynorhinus townsendii	Dermanura phaeotis	0.0000	120	2	-1.75	-1.17	0	11	1.4
Corynorhinus townsendii	Dermanura tolteca	0.0000	120	13	2.72	0.75	0	12.885	5.17
Corynorhinus townsendii	Dermanura watsoni	0.0000	120	0	-0.75	-4.71	0	10.75	0.9
Corynorhinus townsendii	Desmodus rotundus	0.1250	120	33	5.37	0.93	1	21.65	22.7
Corynorhinus townsendii	Diphylla ecaudata	0.5000	120	4	1.65	0.82	1	19.2	17.8
Corynorhinus townsendii	Eptesicus brasiliensis	1.0000	52	1	0.62	0.63	1	9.745	1.11
Corynorhinus townsendii	Eptesicus furinalis	1.0000	52	0	-1.47	-6.07	1	8.985	2.63
Corynorhinus townsendii	Eptesicus fuscus	0.1667	52	49	14.98	1.97	1	13.8	7
Corynorhinus townsendii	Euderma maculatum	1.0000	37	4	10.82	4.5	1	13.25	5.9
Corynorhinus townsendii	Eumops auripendulus	1.0000	108	0	-0.3	-2.88	1	19.35	18.1
Corynorhinus townsendii	Eumops ferox	0.5000	108	0	-0.86	-4.99	1	23.1	25.6
Corynorhinus townsendii	Eumops nanus	1.0000	108	0	-0.42	-3.57	1	11.2	1.8
Corynorhinus townsendii	Eumops perotis	1.0000	108	5	6.1	2.42	1	30.6	40.6
Corynorhinus townsendii	Glossophaga commissarisi	0.0000	120	1	-1.46	-1.35	0	9.685	1.23
Corynorhinus townsendii	Glossophaga soricina	0.2000	120	21	1.85	0.41	0	10.1	0.4
Corynorhinus townsendii	Lasionycteris noctivagans	1.0000	52	1	4.63	3.8	1	10.6	0.6
Corynorhinus townsendii	Lasiurus blossevillii	1.0000	52	21	9.28	1.88	1	9.65	1.3
Corynorhinus townsendii	Lasiurus borealis	1.0000	52	9	6.12	1.89	1	11.3	2
Corynorhinus townsendii	Lasiurus cinereus	1.0000	52	39	17.05	2.42	1	18.55	16.5
Corynorhinus townsendii	Lasiurus ega	1.0000	52	8	4.44	1.5	1	11.2	1.8
Corynorhinus townsendii	Lasiurus intermedius	1.0000	52	13	7.16	1.85	1	16.6	12.6
Corynorhinus townsendii	Lasiurus xanthinus	1.0000	52	23	12.55	2.34	1	13.15	5.7
Corynorhinus townsendii	Lonchorhina aurita	0.0000	120	0	-0.8	-4.86	1	12.8	5
Corynorhinus townsendii	Molossus molossus	0.3333	108	1	0.3	0.31	1	12.05	3.5
Corynorhinus townsendii	Molossus rufus	0.5000	108	6	0.86	0.36	1	21	21.4
Corynorhinus townsendii	Myotis albescens	1.0000	52	1	1.57	1.51	1	7.975	4.65
Corynorhinus townsendii	Myotis californicus	1.0000	52	42	18.01	2.45	1	7.345	5.91
Corynorhinus townsendii	Myotis evotis	1.0000	52	4	5.14	2.3	1	8.58	3.44
Corynorhinus townsendii	Myotis nigricans	0.3333	52	5	1.4	0.63	1	7.27	6.06
Corynorhinus townsendii	Myotis occultus	0.3333	52	0	-0.26	-2.59	1	9.035	2.53

A. Bats' characteristics of Known model

Corynorhinus townsendii	Myotis velifer	0.3333	52	52	16.28	2.06	1	10.04	0.52
Corynorhinus townsendii	Myotis volans	0.0000	52	7	6.24	2.14	1	9.47	1.66
Corynorhinus townsendii	Myotis yumanensis	0.5000	52	20	9.63	1.98	1	7.725	5.15
Corynorhinus townsendii	Nycticeius humeralis	1.0000	52	4	3.2	1.53	1	9.705	1.19
Corynorhinus townsendii	Nyctinomops laticaudatus	0.5000	108	1	-0.22	-0.22	1	11.65	2.7
Corynorhinus townsendii	Nyctinomops macrotis	0.5000	108	16	12.28	2.67	1	15.55	10.5
Corynorhinus townsendii	Parastrellus hesperus	1.0000	52	36	15.28	2.28	1	6.93	6.74
Corynorhinus townsendii	Phyllostomus discolor	0.0000	120	0	-1.16	-5.59	0	23.4	26.2
Corynorhinus townsendii	Pteronotus davyi	0.0000	120	9	1.83	0.61	1	9.89	0.82
Corynorhinus townsendii	Pteronotus parnellii	0.0000	120	14	1.32	0.36	1	14.9	9.2
Corynorhinus townsendii	Sturnira hondurensis	0.0000	120	13	3.75	1.03	0	15.6	10.6
Corynorhinus townsendii	Sturnira lilium	0.0000	120	7	0.77	0.3	0	15.25	9.9
Corynorhinus townsendii	Tadarida brasiliensis	0.1429	108	64	17.61	2.02	1	11.25	1.9
Corynorhinus townsendii	Trachops cirrhosus	0.0000	120	1	0.02	0.03	1	23.6	26.6
Corynorhinus townsendii	Vampyrodes caraccioli	0.0000	120	0	-0.68	-4.54	0	23.05	25.5
Dermanura phaeotis	Dermanura tolteca	0.0000	13.2	88	21.3	2.23	0	13.585	3.77
Dermanura phaeotis	Dermanura watsoni	0.0000	13.2	22	20.11	5.04	1	11.45	0.5
Dermanura phaeotis	Desmodus rotundus	0.0000	62	177	28.87	2.14	0	22.35	21.3
Dermanura phaeotis	Diphylla ecaudata	0.0000	62	21	9.13	1.97	0	19.9	16.4
Dermanura phaeotis	Eptesicus brasiliensis	0.0000	120	6	4.7	1.9	0	10.445	2.51
Dermanura phaeotis	Eptesicus furinalis	0.0000	120	39	16.94	2.66	0	9.685	4.03
Dermanura phaeotis	Eptesicus fuscus	0.0000	120	20	1.01	0.23	0	14.5	5.6
Dermanura phaeotis	Euderma maculatum	0.0000	120	0	-0.53	-4.04	0	13.95	4.5
Dermanura phaeotis	Eumops auripendulus	0.0000	120	2	4.38	3.05	0	20.05	16.7
Dermanura phaeotis	Eumops ferox	0.0000	120	12	8.82	2.49	0	23.8	24.2
Dermanura phaeotis	Eumops nanus	0.0000	120	2	2.79	1.96	0	11.9	0.4
Dermanura phaeotis	Eumops perotis	0.0000	120	0	-1.09	-5.46	0	31.3	39.2
Dermanura phaeotis	Glossophaga commissarisi	0.0000	53.4	77	25.17	2.81	1	10.385	2.63
Dermanura phaeotis	Glossophaga soricina	0.0000	53.4	217	35.5	2.36	1	10.8	1.8
Dermanura phaeotis	Lasionycteris noctivagans	0.0000	120	0	-0.31	-2.94	0	11.3	0.8
Dermanura phaeotis	Lasiurus blossevillii	0.0000	120	30	8.46	1.55	0	10.35	2.7
Dermanura phaeotis	Lasiurus borealis	0.0000	120	9	3.36	1.14	0	12	0.6
Dermanura phaeotis	Lasiurus cinereus	0.0000	120	9	0.07	0.02	0	19.25	15.1
Dermanura phaeotis	Lasiurus ega	0.0000	120	32	14.4	2.49	0	11.9	0.4

A. Bats' characteristics of Known model

Dermanura phaeotis	Lasiurus intermedius	0.0000	120	33	13.29	2.27	0	17.3	11.2
Dermanura phaeotis	Lasiurus xanthinus	0.0000	120	25	8.45	1.69	0	13.85	4.3
Dermanura phaeotis	Lonchorhina aurita	0.0000	55.6	18	14.93	3.54	0	13.5	3.6
Dermanura phaeotis	Molossus molossus	0.0000	120	13	9.47	2.57	0	12.75	2.1
Dermanura phaeotis	Molossus rufus	0.0000	120	76	23.19	2.6	0	21.7	20
Dermanura phaeotis	Myotis albescens	0.0000	120	4	5.09	2.49	0	8.675	6.05
Dermanura phaeotis	Myotis californicus	0.0000	120	8	-0.41	-0.15	0	8.045	7.31
Dermanura phaeotis	Myotis evotis	0.0000	120	0	-1.02	-5.34	0	9.28	4.84
Dermanura phaeotis	Myotis nigricans	0.0000	120	45	16.96	2.48	0	7.97	7.46
Dermanura phaeotis	Myotis occultus	0.0000	120	0	-0.38	-3.35	0	9.735	3.93
Dermanura phaeotis	Myotis velifer	0.0000	120	15	-0.19	-0.05	0	10.74	1.92
Dermanura phaeotis	Myotis volans	0.0000	120	0	-1.44	-6.03	0	10.17	3.06
Dermanura phaeotis	Myotis yumanensis	0.0000	120	2	-1.81	-1.21	0	8.425	6.55
Dermanura phaeotis	Nycticeius humeralis	0.0000	120	0	-1.43	-6.01	0	10.405	2.59
Dermanura phaeotis	Nyctinomops laticaudatus	0.0000	120	21	11.76	2.51	0	12.35	1.3
Dermanura phaeotis	Nyctinomops macrotis	0.0000	120	10	4.17	1.33	0	16.25	9.1
Dermanura phaeotis	Parastrellus hesperus	0.0000	120	1	-2.74	-2.24	0	7.63	8.14
Dermanura phaeotis	Phyllostomus discolor	0.0000	55.6	33	18.84	3.25	1	24.1	24.8
Dermanura phaeotis	Pteronotus davyi	0.0000	85	88	24.8	2.59	0	10.59	2.22
Dermanura phaeotis	Pteronotus parnellii	0.0000	85	177	35.39	2.6	0	15.6	7.8
Dermanura phaeotis	Sturnira hondurensis	0.0000	34.2	43	10.67	1.63	0	16.3	9.2
Dermanura phaeotis	Sturnira lilium	0.0000	34.2	107	29.84	2.83	1	15.95	8.5
Dermanura phaeotis	Tadarida brasiliensis	0.0000	120	21	0.2	0.05	0	11.95	0.5
Dermanura phaeotis	Trachops cirrhosus	0.0000	55.6	30	20.09	3.75	0	24.3	25.2
Dermanura phaeotis	Vampyrodes caraccioli	0.0000	26.2	16	15.83	4.21	0	23.75	24.1
Dermanura tolteca	Dermanura watsoni	1.0000	13.2	11	9.59	2.83	0	13.335	4.27
Dermanura tolteca	Desmodus rotundus	0.1250	62	191	31.86	2.26	0	24.235	17.53
Dermanura tolteca	Diphylla ecaudata	0.0000	62	34	16.13	2.7	0	21.785	12.63
Dermanura tolteca	Eptesicus brasiliensis	0.0000	120	16	14.44	3.64	0	12.33	6.28
Dermanura tolteca	Eptesicus furinalis	0.0000	120	32	13.63	2.36	0	11.57	7.8
Dermanura tolteca	Eptesicus fuscus	0.0000	120	63	12.12	1.53	0	16.385	1.83
Dermanura tolteca	Euderma maculatum	0.0000	120	0	-0.53	-4.02	0	15.835	0.73
Dermanura tolteca	Eumops auripendulus	0.0000	120	2	4.42	3.07	0	21.935	12.93
Dermanura tolteca	Eumops ferox	0.0000	120	8	5.51	1.93	0	25.685	20.43

A. Bats' characteristics of Known model

Dermanura tolteca	Eumops nanus	0.0000	120	2	2.82	1.97	0	13.785	3.37
Dermanura tolteca	Eumops perotis	0.0000	120	5	3.77	1.68	0	33.185	35.43
Dermanura tolteca	Glossophaga commissarisi	1.0000	53.4	82	27.21	2.94	0	12.27	6.4
Dermanura tolteca	Glossophaga soricina	0.0000	53.4	189	30.48	2.18	0	12.685	5.57
Dermanura tolteca	Lasionycteris noctivagans	0.0000	120	1	3.12	3.07	0	13.185	4.57
Dermanura tolteca	Lasiurus blossevillii	0.0000	120	51	16.48	2.26	0	12.235	6.47
Dermanura tolteca	Lasiurus borealis	0.0000	120	20	9.79	2.15	0	13.885	3.17
Dermanura tolteca	Lasiurus cinereus	0.0000	120	30	7.41	1.36	0	21.135	11.33
Dermanura tolteca	Lasiurus ega	0.0000	120	27	11.93	2.25	0	13.785	3.37
Dermanura tolteca	Lasiurus intermedius	0.0000	120	36	14.83	2.42	0	19.185	7.43
Dermanura tolteca	Lasiurus xanthinus	0.0000	120	18	5.48	1.3	0	15.735	0.53
Dermanura tolteca	Lonchorhina aurita	0.0000	55.6	11	8.74	2.57	0	15.385	0.17
Dermanura tolteca	Molossus molossus	0.0000	120	14	10.39	2.71	0	14.635	1.67
Dermanura tolteca	Molossus rufus	0.0000	120	59	17.48	2.23	0	23.585	16.23
Dermanura tolteca	Myotis albescens	0.0000	120	5	6.6	2.88	0	10.56	9.82
Dermanura tolteca	Myotis californicus	0.0000	120	25	5.41	1.1	0	9.93	11.08
Dermanura tolteca	Myotis evotis	0.0000	120	0	-1.01	-5.32	0	11.165	8.61
Dermanura tolteca	Myotis nigricans	0.0000	120	68	27.09	3.24	0	9.855	11.23
Dermanura tolteca	Myotis occultus	0.0000	120	0	-0.37	-3.33	0	11.62	7.7
Dermanura tolteca	Myotis velifer	0.0000	120	56	10.51	1.41	0	12.625	5.69
Dermanura tolteca	Myotis volans	0.0000	120	2	0.03	0.02	0	12.055	6.83
Dermanura tolteca	Myotis yumanensis	0.0000	120	18	4.69	1.12	0	10.31	10.32
Dermanura tolteca	Nycticeius humeralis	0.0000	120	6	3.02	1.25	0	12.29	6.36
Dermanura tolteca	Nyctinomops laticaudatus	0.0000	120	15	8.01	2.04	0	14.235	2.47
Dermanura tolteca	Nyctinomops macrotis	0.5000	120	14	6.6	1.75	0	18.135	5.33
Dermanura tolteca	Parastrellus hesperus	0.0000	120	22	4.47	0.97	0	9.515	11.91
Dermanura tolteca	Phyllostomus discolor	0.0000	55.6	32	18.36	3.2	0	25.985	21.03
Dermanura tolteca	Pteronotus davyi	0.0000	85	81	22.76	2.47	0	12.475	5.99
Dermanura tolteca	Pteronotus parnellii	0.0000	85	145	28.41	2.31	0	17.485	4.03
Dermanura tolteca	Sturnira hondurensis	1.0000	34.2	98	28.68	2.83	1	18.185	5.43
Dermanura tolteca	Sturnira lilium	0.2000	34.2	105	29.45	2.81	0	17.835	4.73
Dermanura tolteca	Tadarida brasiliensis	0.0000	120	60	9.22	1.21	0	13.835	3.27
Dermanura tolteca	Trachops cirrhosus	0.5000	55.6	21	13.74	2.93	0	26.185	21.43
Dermanura tolteca	Vampyrodes caraccioli	0.0000	26.2	14	13.83	3.76	1	25.635	20.33

A. Bats' characteristics of Known model

Dermanura watsoni	Desmodus rotundus	0.1250	62	19	10.76	2.1	0	22.1	21.8
Dermanura watsoni	Diphylla ecaudata	0.0000	62	6	10.05	3	0	19.65	16.9
Dermanura watsoni	Eptesicus brasiliensis	0.0000	120	3	9.36	3.56	0	10.195	2.01
Dermanura watsoni	Eptesicus furinalis	0.0000	120	3	4.3	2.11	0	9.435	3.53
Dermanura watsoni	Eptesicus fuscus	0.0000	120	3	1.39	0.79	0	14.25	6.1
Dermanura watsoni	Euderma maculatum	0.0000	120	0	-0.15	-1.54	0	13.7	5
Dermanura watsoni	Eumops auripendulus	0.0000	120	1	7.94	4.46	0	19.8	17.2
Dermanura watsoni	Eumops ferox	0.0000	120	2	5.26	2.81	0	23.55	24.7
Dermanura watsoni	Eumops nanus	0.0000	120	0	-0.18	-1.83	0	11.65	0.9
Dermanura watsoni	Eumops perotis	0.0000	120	0	-0.31	-2.97	0	31.05	39.7
Dermanura watsoni	Glossophaga commissarisi	1.0000	53.4	12	13.8	2.94	1	10.135	2.13
Dermanura watsoni	Glossophaga soricina	0.0000	53.4	21	11.7	2.15	1	10.55	1.3
Dermanura watsoni	Lasionycteris noctivagans	0.0000	120	0	-0.09	-0.44	0	11.05	0.3
Dermanura watsoni	Lasiurus blossevillii	0.0000	120	2	1.71	1.15	0	10.1	2.2
Dermanura watsoni	Lasiurus borealis	0.0000	120	1	1.41	1.32	0	11.75	1.1
Dermanura watsoni	Lasiurus cinereus	0.0000	120	0	-0.87	-5.02	0	19	15.6
Dermanura watsoni	Lasiurus ega	0.0000	120	2	2.85	1.79	0	11.65	0.9
Dermanura watsoni	Lasiurus intermedius	0.0000	120	1	0.94	0.91	0	17.05	11.7
Dermanura watsoni	Lasiurus xanthinus	0.0000	120	0	-0.69	-4.56	0	13.6	4.8
Dermanura watsoni	Lonchorhina aurita	0.0000	55.6	7	20.62	4.41	0	13.25	4.1
Dermanura watsoni	Molossus molossus	0.0000	120	1	2.4	2.06	0	12.5	2.6
Dermanura watsoni	Molossus rufus	0.0000	120	8	8.37	2.4	0	21.45	20.5
Dermanura watsoni	Myotis albescens	0.0000	120	2	9.52	4.05	0	8.425	5.55
Dermanura watsoni	Myotis californicus	0.0000	120	1	0.24	0.24	0	7.795	6.81
Dermanura watsoni	Myotis evotis	0.0000	120	0	-0.29	-2.84	0	9.03	4.34
Dermanura watsoni	Myotis nigricans	0.0000	120	8	10.84	2.87	0	7.72	6.96
Dermanura watsoni	Myotis occultus	0.0000	120	0	-0.11	-0.85	0	9.485	3.43
Dermanura watsoni	Myotis velifer	0.0000	120	0	-1.16	-5.6	0	10.49	1.42
Dermanura watsoni	Myotis volans	0.0000	120	0	-0.41	-3.53	0	9.92	2.56
Dermanura watsoni	Myotis yumanensis	0.0000	120	0	-0.75	-4.72	0	8.175	6.05
Dermanura watsoni	Nycticeius humeralis	0.0000	120	0	-0.41	-3.51	0	10.155	2.09
Dermanura watsoni	Nyctinomops laticaudatus	0.0000	120	0	-0.47	-3.79	0	12.1	1.8
Dermanura watsoni	Nyctinomops macrotis	0.5000	120	1	1.48	1.38	0	16	9.6
Dermanura watsoni	Parastrellus hesperus	0.0000	120	0	-0.88	-5.05	0	7.38	7.64

A. Bats' characteristics of Known model

Dermanura watsoni	Phyllostomus discolor	0.0000	55.6	8	16.17	3.68	1	23.85	25.3
Dermanura watsoni	Pteronotus davyi	0.0000	85	9	8.67	2.36	0	10.34	1.72
Dermanura watsoni	Pteronotus parnellii	0.0000	85	18	12.29	2.37	0	15.35	8.3
Dermanura watsoni	Sturnira hondurensis	1.0000	34.2	7	6.65	2.13	0	16.05	9.7
Dermanura watsoni	Sturnira lilium	0.2000	34.2	17	16.69	2.97	1	15.7	9
Dermanura watsoni	Tadarida brasiliensis	0.0000	120	1	-0.55	-0.54	0	11.7	1
Dermanura watsoni	Trachops cirrhosus	0.5000	55.6	8	18.81	4.02	0	24.05	25.7
Dermanura watsoni	Vampyrodes caraccioli	0.0000	26.2	5	17.31	4.39	0	23.5	24.6
Desmodus rotundus	Diphylla ecaudata	0.1111	51.8	61	19.81	3.27	1	30.55	4.9
Desmodus rotundus	Eptesicus brasiliensis	0.1250	120	17	9.94	3	1	21.095	23.81
Desmodus rotundus	Eptesicus furinalis	0.1250	120	63	18.55	2.86	1	20.335	25.33
Desmodus rotundus	Eptesicus fuscus	0.0769	120	111	13.87	1.46	1	25.15	15.7
Desmodus rotundus	Euderma maculatum	0.1250	120	2	1.98	1.55	1	24.6	16.8
Desmodus rotundus	Eumops auripendulus	0.1250	120	2	2.75	2.25	1	30.7	4.6
Desmodus rotundus	Eumops ferox	0.1111	120	24	12.34	3.23	1	34.45	2.9
Desmodus rotundus	Eumops nanus	0.1250	120	6	6.29	3.34	1	22.55	20.9
Desmodus rotundus	Eumops perotis	0.1250	120	7	3.13	1.3	1	41.95	17.9
Desmodus rotundus	Glossophaga commissarisi	0.1250	62	109	23.72	2.75	0	21.035	23.93
Desmodus rotundus	Glossophaga soricina	0.1818	62	389	43.64	2.65	0	21.45	23.1
Desmodus rotundus	Lasionycteris noctivagans	0.1250	120	1	1.94	2.25	1	21.95	22.1
Desmodus rotundus	Lasiurus blossevillii	0.1250	120	82	17.53	2.23	1	21	24
Desmodus rotundus	Lasiurus borealis	0.1250	120	31	9.88	2.02	1	22.65	20.7
Desmodus rotundus	Lasiurus cinereus	0.1250	120	53	8.37	1.26	1	29.9	6.2
Desmodus rotundus	Lasiurus ega	0.1250	120	50	15.07	2.52	1	22.55	20.9
Desmodus rotundus	Lasiurus intermedius	0.1250	120	74	21.22	3.12	1	27.95	10.1
Desmodus rotundus	Lasiurus xanthinus	0.1250	120	40	8.66	1.52	1	24.5	17
Desmodus rotundus	Lonchorhina aurita	0.0000	62	17	8.98	2.59	1	24.15	17.7
Desmodus rotundus	Molossus molossus	0.1000	120	24	12.1	3.12	1	23.4	19.2
Desmodus rotundus	Molossus rufus	0.1111	120	128	26.63	2.89	1	32.35	1.3
Desmodus rotundus	Myotis albescens	0.1250	120	6	5.07	2.43	1	19.325	27.35
Desmodus rotundus	Myotis californicus	0.1250	120	57	8.93	1.3	1	18.695	28.61
Desmodus rotundus	Myotis evotis	0.1250	120	0	-1.53	-6.14	1	19.93	26.14
Desmodus rotundus	Myotis nigricans	0.1000	120	95	25.26	3.4	1	18.62	28.76
Desmodus rotundus	Myotis occultus	0.1000	120	2	3.36	2.94	1	20.385	25.23

A. Bats' characteristics of Known model

Desmodus rotundus	Myotis velifer	0.1000	120	106	13.22	1.42	1	21.39	23.22
Desmodus rotundus	Myotis volans	0.0000	120	5	0.41	0.19	1	20.82	24.36
Desmodus rotundus	Myotis yumanensis	0.1111	120	43	8.28	1.39	1	19.075	27.85
Desmodus rotundus	Nycticeius humeralis	0.1250	120	13	4.61	1.41	1	21.055	23.89
Desmodus rotundus	Nyctinomops laticaudatus	0.1111	120	37	13.72	2.72	1	23	20
Desmodus rotundus	Nyctinomops macrotis	0.2500	120	29	9.5	2	1	26.9	12.2
Desmodus rotundus	Parastrellus hesperus	0.1250	120	38	4.5	0.79	1	18.28	29.44
Desmodus rotundus	Phyllostomus discolor	0.0000	62	37	13.72	2.72	0	34.75	3.5
Desmodus rotundus	Pteronotus davyi	0.0000	85	147	28.19	2.84	1	21.24	23.52
Desmodus rotundus	Pteronotus parnellii	0.1667	85	298	40.63	2.89	1	26.25	13.5
Desmodus rotundus	Sturnira hondurensis	0.1250	62	130	24.82	2.59	0	26.95	12.1
Desmodus rotundus	Sturnira lilium	0.0833	62	152	28.25	2.78	0	26.6	12.8
Desmodus rotundus	Tadarida brasiliensis	0.0714	120	125	13.26	1.3	1	22.6	20.8
Desmodus rotundus	Trachops cirrhosus	0.1111	62	28	12.01	2.75	1	34.95	3.9
Desmodus rotundus	Vampyrodes caraccioli	0.0000	62	11	6.67	2.34	0	34.4	2.8
Diphylla ecaudata	Eptesicus brasiliensis	0.5000	120	4	6.54	2.69	1	18.645	18.91
Diphylla ecaudata	Eptesicus furinalis	0.5000	120	16	13.32	2.72	1	17.885	20.43
Diphylla ecaudata	Eptesicus fuscus	0.1429	120	17	5.86	1.35	1	22.7	10.8
Diphylla ecaudata	Euderma maculatum	0.5000	120	0	-0.28	-2.75	1	22.15	11.9
Diphylla ecaudata	Eumops auripendulus	0.5000	120	1	4.22	3.25	1	28.25	0.3
Diphylla ecaudata	Eumops ferox	0.3333	120	5	7.08	2.62	1	32	7.8
Diphylla ecaudata	Eumops nanus	0.5000	120	3	9.11	3.83	1	20.1	16
Diphylla ecaudata	Eumops perotis	0.5000	120	2	2.99	1.9	1	39.5	22.8
Diphylla ecaudata	Glossophaga commissarisi	0.0000	62	12	6.55	1.74	0	18.585	19.03
Diphylla ecaudata	Glossophaga soricina	0.1667	62	58	17.33	2.02	0	19	18.2
Diphylla ecaudata	Lasionycteris noctivagans	0.5000	120	1	6.12	4.34	1	19.5	17.2
Diphylla ecaudata	Lasiurus blossevillii	0.5000	120	10	5.46	1.6	1	18.55	19.1
Diphylla ecaudata	Lasiurus borealis	0.5000	120	4	3.3	1.54	1	20.2	15.8
Diphylla ecaudata	Lasiurus cinereus	0.5000	120	6	2.23	0.89	1	27.45	1.3
Diphylla ecaudata	Lasiurus ega	0.5000	120	12	10.3	2.5	1	20.1	16
Diphylla ecaudata	Lasiurus intermedius	0.5000	120	10	7.51	2.09	1	25.5	5.2
Diphylla ecaudata	Lasiurus xanthinus	0.5000	120	6	3.54	1.37	1	22.05	12.1
Diphylla ecaudata	Lonchorhina aurita	0.0000	62	9	14.24	3.55	1	21.7	12.8
Diphylla ecaudata	Molossus molossus	0.2500	120	4	5.43	2.33	1	20.95	14.3

A. Bats' characteristics of Known model

Diphylla ecaudata	Molossus rufus	0.3333	120	29	16.87	2.6	1	29.9	3.6
Diphylla ecaudata	Myotis albescens	0.5000	120	3	7.66	3.36	1	16.875	22.45
Diphylla ecaudata	Myotis californicus	0.5000	120	4	0.86	0.43	1	16.245	23.71
Diphylla ecaudata	Myotis evotis	0.5000	120	0	-0.53	-4.05	1	17.48	21.24
Diphylla ecaudata	Myotis nigricans	0.2500	120	16	11.45	2.46	1	16.17	23.86
Diphylla ecaudata	Myotis occultus	0.2500	120	0	-0.2	-2.06	1	17.935	20.33
Diphylla ecaudata	Myotis velifer	0.2500	120	17	5.96	1.37	1	18.94	18.32
Diphylla ecaudata	Myotis volans	0.0000	120	0	-0.76	-4.74	1	18.37	19.46
Diphylla ecaudata	Myotis yumanensis	0.3333	120	3	0.85	0.5	1	16.625	22.95
Diphylla ecaudata	Nycticeius humeralis	0.5000	120	1	0.61	0.61	1	18.605	18.99
Diphylla ecaudata	Nyctinomops laticaudatus	0.3333	120	12	13.27	3.02	1	20.55	15.1
Diphylla ecaudata	Nyctinomops macrotis	0.3333	120	1	0.17	0.17	1	24.45	7.3
Diphylla ecaudata	Parastrellus hesperus	0.5000	120	2	-0.36	-0.25	1	15.83	24.54
Diphylla ecaudata	Phyllostomus discolor	0.0000	62	10	10.59	2.73	0	32.3	8.4
Diphylla ecaudata	Pteronotus davyi	0.0000	85	23	11.82	2.16	1	18.79	18.62
Diphylla ecaudata	Pteronotus parnellii	0.0000	85	46	16.76	2.16	1	23.8	8.6
Diphylla ecaudata	Sturnira hondurensis	0.0000	62	30	16.2	2.49	0	24.5	7.2
Diphylla ecaudata	Sturnira lilium	0.0000	62	34	17.7	2.54	0	24.15	7.9
Diphylla ecaudata	Tadarida brasiliensis	0.1250	120	20	6.01	1.29	1	20.15	15.9
Diphylla ecaudata	Trachops cirrhosus	0.3333	62	7	8.51	2.65	1	32.5	8.8
Diphylla ecaudata	Vampyrodes caraccioli	0.0000	62	3	5.3	2.55	0	31.95	7.7
Eptesicus brasiliensis	Eptesicus furinalis	1.0000	19	13	9.93	2.28	1	8.43	1.52
Eptesicus brasiliensis	Eptesicus fuscus	0.1667	19	7	10.85	3	1	13.245	8.11
Eptesicus brasiliensis	Euderma maculatum	1.0000	52	0	-0.15	-1.54	1	12.695	7.01
Eptesicus brasiliensis	Eumops auripendulus	1.0000	108	0	-0.12	-1.13	1	18.795	19.21
Eptesicus brasiliensis	Eumops ferox	0.5000	108	3	8.06	3.25	1	22.545	26.71
Eptesicus brasiliensis	Eumops nanus	1.0000	108	1	5.52	3.61	1	10.645	2.91
Eptesicus brasiliensis	Eumops perotis	1.0000	108	0	-0.31	-2.97	1	30.045	41.71
Eptesicus brasiliensis	Glossophaga commissarisi	0.0000	120	6	6.49	2.21	0	9.13	0.12
Eptesicus brasiliensis	Glossophaga soricina	0.2000	120	18	9.81	2	0	9.545	0.71
Eptesicus brasiliensis	Lasionycteris noctivagans	1.0000	52	0	-0.09	-0.44	1	10.045	1.71
Eptesicus brasiliensis	Lasiurus blossevillii	1.0000	52	5	5.48	2.09	1	9.095	0.19
Eptesicus brasiliensis	Lasiurus borealis	1.0000	52	3	5.26	2.45	1	10.745	3.11
Eptesicus brasiliensis	Lasiurus cinereus	1.0000	52	2	1.44	0.99	1	17.995	17.61

A. Bats' characteristics of Known model

Eptesicus brasiliensis	Lasiurus ega	1.0000	52	3	4.57	2.21	1	10.645	2.91
Eptesicus brasiliensis	Lasiurus intermedius	1.0000	52	4	5.66	2.32	1	16.045	13.71
Eptesicus brasiliensis	Lasiurus xanthinus	1.0000	52	1	0.76	0.75	1	12.595	6.81
Eptesicus brasiliensis	Lonchorhina aurita	0.0000	120	3	8.65	3.39	1	12.245	6.11
Eptesicus brasiliensis	Molossus molossus	0.3333	108	2	5.17	2.78	1	11.495	4.61
Eptesicus brasiliensis	Molossus rufus	0.5000	108	7	7.21	2.26	1	20.445	22.51
Eptesicus brasiliensis	Myotis albescens	1.0000	52	3	14.38	4.57	1	7.42	3.54
Eptesicus brasiliensis	Myotis californicus	1.0000	52	2	1.37	0.94	1	6.79	4.8
Eptesicus brasiliensis	Myotis evotis	1.0000	52	0	-0.29	-2.84	1	8.025	2.33
Eptesicus brasiliensis	Myotis nigricans	0.3333	52	9	12.28	3	1	6.715	4.95
Eptesicus brasiliensis	Myotis occultus	0.3333	52	0	-0.11	-0.85	1	8.48	1.42
Eptesicus brasiliensis	Myotis velifer	0.3333	52	7	4.88	1.66	1	9.485	0.59
Eptesicus brasiliensis	Myotis volans	0.0000	52	0	-0.41	-3.53	1	8.915	0.55
Eptesicus brasiliensis	Myotis yumanensis	0.5000	52	0	-0.75	-4.72	1	7.17	4.04
Eptesicus brasiliensis	Nycticeius humeralis	1.0000	52	0	-0.41	-3.51	1	9.15	0.08
Eptesicus brasiliensis	Nyctinomops laticaudatus	0.5000	108	2	3.8	2.24	1	11.095	3.81
Eptesicus brasiliensis	Nyctinomops macrotis	0.5000	108	2	3.46	2.09	1	14.995	11.61
Eptesicus brasiliensis	Parastrellus hesperus	1.0000	52	0	-0.88	-5.05	1	6.375	5.63
Eptesicus brasiliensis	Phyllostomus discolor	0.0000	120	4	7.84	2.91	0	22.845	27.31
Eptesicus brasiliensis	Pteronotus davyi	0.0000	120	8	7.6	2.24	1	9.335	0.29
Eptesicus brasiliensis	Pteronotus parnellii	0.0000	120	11	7	1.86	1	14.345	10.31
Eptesicus brasiliensis	Sturnira hondurensis	0.0000	120	11	10.97	2.6	0	15.045	11.71
Eptesicus brasiliensis	Sturnira lilium	0.0000	120	13	12.53	2.69	0	14.695	11.01
Eptesicus brasiliensis	Tadarida brasiliensis	0.1429	108	5	2.5	1.07	1	10.695	3.01
Eptesicus brasiliensis	Trachops cirrhosus	0.0000	120	2	4.39	2.49	1	23.045	27.71
Eptesicus brasiliensis	Vampyrodes caraccioli	0.0000	120	1	3.23	2.56	0	22.495	26.61
Eptesicus furinalis	Eptesicus fuscus	0.1667	19	20	6.4	1.36	1	12.485	9.63
Eptesicus furinalis	Euderma maculatum	1.0000	52	0	-0.3	-2.91	1	11.935	8.53
Eptesicus furinalis	Eumops auripendulus	1.0000	108	0	-0.25	-2.5	1	18.035	20.73
Eptesicus furinalis	Eumops ferox	0.5000	108	6	7.89	2.68	1	21.785	28.23
Eptesicus furinalis	Eumops nanus	1.0000	108	3	8.38	3.68	1	9.885	4.43
Eptesicus furinalis	Eumops perotis	1.0000	108	0	-0.62	-4.33	1	29.285	43.23
Eptesicus furinalis	Glossophaga commissarisi	0.0000	120	17	8.95	1.96	0	8.37	1.4
Eptesicus furinalis	Glossophaga soricina	0.2000	120	72	20.12	2.1	0	8.785	2.23

A. Bats' characteristics of Known model

Eptesicus furinalis	Lasionycteris noctivagans	1.0000	52	0	-0.17	-1.81	1	9.285	3.23
Eptesicus furinalis	Lasiurus blossevillii	1.0000	52	16	8.67	1.95	1	8.335	1.33
Eptesicus furinalis	Lasiurus borealis	1.0000	52	5	3.89	1.62	1	9.985	4.63
Eptesicus furinalis	Lasiurus cinereus	1.0000	52	6	1.81	0.74	1	17.235	19.13
Eptesicus furinalis	Lasiurus ega	1.0000	52	21	17.27	3.03	1	9.885	4.43
Eptesicus furinalis	Lasiurus intermedius	1.0000	52	16	11.59	2.47	1	15.285	15.23
Eptesicus furinalis	Lasiurus xanthinus	1.0000	52	10	6.05	1.76	1	11.835	8.33
Eptesicus furinalis	Lonchorhina aurita	0.0000	120	3	6.98	2.62	1	11.485	7.63
Eptesicus furinalis	Molossus molossus	0.3333	108	6	7.75	2.65	1	10.735	6.13
Eptesicus furinalis	Molossus rufus	0.5000	108	38	20.68	2.77	1	19.685	24.03
Eptesicus furinalis	Myotis albescens	1.0000	52	2	4.55	2.68	1	6.66	2.02
Eptesicus furinalis	Myotis californicus	1.0000	52	7	2.27	0.85	1	6.03	3.28
Eptesicus furinalis	Myotis evotis	1.0000	52	0	-0.58	-4.2	1	7.265	0.81
Eptesicus furinalis	Myotis nigricans	0.3333	52	23	15.55	2.7	1	5.955	3.43
Eptesicus furinalis	Myotis occultus	0.3333	52	0	-0.21	-2.21	1	7.72	0.1
Eptesicus furinalis	Myotis velifer	0.3333	52	12	2.98	0.85	1	8.725	2.11
Eptesicus furinalis	Myotis volans	0.0000	52	1	0.42	0.43	1	8.155	0.97
Eptesicus furinalis	Myotis yumanensis	0.5000	52	1	-0.79	-0.77	1	6.41	2.52
Eptesicus furinalis	Nycticeius humeralis	1.0000	52	4	4.21	1.91	1	8.39	1.44
Eptesicus furinalis	Nyctinomops laticaudatus	0.5000	108	9	8.88	2.51	1	10.335	5.33
Eptesicus furinalis	Nyctinomops macrotis	0.5000	108	2	1.02	0.72	1	14.235	13.13
Eptesicus furinalis	Parastrellus hesperus	1.0000	52	1	-1.17	-1.11	1	5.615	4.11
Eptesicus furinalis	Phyllostomus discolor	0.0000	120	13	12.86	2.9	0	22.085	28.83
Eptesicus furinalis	Pteronotus davyi	0.0000	120	27	12.86	2.18	1	8.575	1.81
Eptesicus furinalis	Pteronotus parnellii	0.0000	120	45	14.76	1.98	1	13.585	11.83
Eptesicus furinalis	Sturnira hondurensis	0.0000	120	24	11.42	2.08	0	14.285	13.23
Eptesicus furinalis	Sturnira lilium	0.0000	120	32	15.05	2.31	0	13.935	12.53
Eptesicus furinalis	Tadarida brasiliensis	0.1429	108	5	-0.66	-0.29	1	9.935	4.53
Eptesicus furinalis	Trachops cirrhosus	0.0000	120	7	7.76	2.49	1	22.285	29.23
Eptesicus furinalis	Vampyrodes caraccioli	0.0000	120	1	1.23	1.19	0	21.735	28.13
Eptesicus fuscus	Euderma maculatum	0.1667	52	5	8.36	4.45	1	16.75	1.1
Eptesicus fuscus	Eumops auripendulus	0.1667	108	0	-0.48	-3.84	1	22.85	11.1
Eptesicus fuscus	Eumops ferox	0.1429	108	3	0.9	0.54	1	26.6	18.6
Eptesicus fuscus	Eumops nanus	0.1667	108	0	-0.68	-4.53	1	14.7	5.2

A. Bats' characteristics of Known model

Eptesicus fuscus	Eumops perotis	0.1667	108	15	11.95	3.25	1	34.1	33.6
Eptesicus fuscus	Glossophaga commissarisi	0.0000	120	15	1.78	0.48	0	13.185	8.23
Eptesicus fuscus	Glossophaga soricina	0.1000	120	88	9.01	0.99	0	13.6	7.4
Eptesicus fuscus	Lasionycteris noctivagans	0.1667	52	1	2.76	2.84	1	14.1	6.4
Eptesicus fuscus	Lasiurus blossevillii	0.1667	52	65	19.1	2.41	1	13.15	8.3
Eptesicus fuscus	Lasiurus borealis	0.1667	52	28	12.66	2.44	1	14.8	5
Eptesicus fuscus	Lasiurus cinereus	0.1667	52	89	24.59	2.67	1	22.05	9.5
Eptesicus fuscus	Lasiurus ega	0.1667	52	22	8.02	1.75	1	14.7	5.2
Eptesicus fuscus	Lasiurus intermedius	0.1667	52	44	16.36	2.52	1	20.1	5.6
Eptesicus fuscus	Lasiurus xanthinus	0.1667	52	45	15.12	2.29	1	16.65	1.3
Eptesicus fuscus	Lonchorhina aurita	0.0000	120	2	0.33	0.24	1	16.3	2
Eptesicus fuscus	Molossus molossus	0.1250	108	7	3.86	1.49	1	15.55	3.5
Eptesicus fuscus	Molossus rufus	0.1429	108	25	4.48	0.93	1	24.5	14.4
Eptesicus fuscus	Myotis albescens	0.1667	52	5	5.81	2.66	1	11.475	11.65
Eptesicus fuscus	Myotis californicus	0.1667	52	102	27.88	2.84	1	10.845	12.91
Eptesicus fuscus	Myotis evotis	0.1667	52	12	10.09	3.03	1	12.08	10.44
Eptesicus fuscus	Myotis nigricans	0.1250	52	23	6.33	1.35	1	10.77	13.06
Eptesicus fuscus	Myotis occultus	0.1250	52	2	4.65	3.53	1	12.535	9.53
Eptesicus fuscus	Myotis velifer	0.1250	52	121	23.9	2.21	1	13.54	7.52
Eptesicus fuscus	Myotis volans	0.0000	52	31	18.9	3.71	1	12.97	8.66
Eptesicus fuscus	Myotis yumanensis	0.1429	52	44	13.19	2.02	1	11.225	12.15
Eptesicus fuscus	Nycticeius humeralis	0.1667	52	13	7.11	2.01	1	13.205	8.19
Eptesicus fuscus	Nyctinomops laticaudatus	0.1429	108	10	3.99	1.3	1	15.15	4.3
Eptesicus fuscus	Nyctinomops macrotis	0.1429	108	27	12.62	2.48	1	19.05	3.5
Eptesicus fuscus	Parastrellus hesperus	0.1667	52	77	20.4	2.37	1	10.43	13.74
Eptesicus fuscus	Phyllostomus discolor	0.0000	120	9	3.23	1.11	0	26.9	19.2
Eptesicus fuscus	Pteronotus davyi	0.0000	120	38	7.4	1.23	1	13.39	7.82
Eptesicus fuscus	Pteronotus parnellii	0.0000	120	76	10.51	1.24	1	18.4	2.2
Eptesicus fuscus	Sturnira hondurensis	0.0000	120	69	16.72	2.05	0	19.1	3.6
Eptesicus fuscus	Sturnira lilium	0.0000	120	40	7.56	1.23	0	18.75	2.9
Eptesicus fuscus	Tadarida brasiliensis	0.0833	108	156	27.31	2.22	1	14.75	5.1
Eptesicus fuscus	Trachops cirrhosus	0.0000	120	7	2.96	1.15	1	27.1	19.6
Eptesicus fuscus	Vampyrodes caraccioli	0.0000	120	1	-0.15	-0.15	0	26.55	18.5
Euderma maculatum	Eumops auripendulus	1.0000	108	0	-0.06	0.3	1	22.3	12.2

A. Bats' characteristics of Known model

Euderma maculatum	Eumops ferox	0.5000	108	0	-0.17	-1.81	1	26.05	19.7
Euderma maculatum	Eumops nanus	1.0000	108	0	-0.09	-0.4	1	14.15	4.1
Euderma maculatum	Eumops perotis	1.0000	108	0	-0.15	-1.54	1	33.55	34.7
Euderma maculatum	Glossophaga commissarisi	0.0000	120	0	-0.4	-3.48	0	12.635	7.13
Euderma maculatum	Glossophaga soricina	0.2000	120	2	1.8	1.2	0	13.05	6.3
Euderma maculatum	Lasionycteris noctivagans	1.0000	52	0	-0.04	0.99	1	13.55	5.3
Euderma maculatum	Lasiurus blossevillii	1.0000	52	1	2.17	1.89	1	12.6	7.2
Euderma maculatum	Lasiurus borealis	1.0000	52	0	-0.25	-2.57	1	14.25	3.9
Euderma maculatum	Lasiurus cinereus	1.0000	52	5	11.34	3.34	1	21.5	10.6
Euderma maculatum	Lasiurus ega	1.0000	52	0	-0.29	-2.79	1	14.15	4.1
Euderma maculatum	Lasiurus intermedius	1.0000	52	2	6.1	3.04	1	19.55	6.7
Euderma maculatum	Lasiurus xanthinus	1.0000	52	0	-0.34	-3.13	1	16.1	0.2
Euderma maculatum	Lonchorhina aurita	0.0000	120	0	-0.16	-1.68	1	15.75	0.9
Euderma maculatum	Molossus molossus	0.3333	108	0	-0.18	-1.84	1	15	2.4
Euderma maculatum	Molossus rufus	0.5000	108	0	-0.43	-3.59	1	23.95	15.5
Euderma maculatum	Myotis albescens	1.0000	52	0	-0.1	-0.72	1	10.925	10.55
Euderma maculatum	Myotis californicus	1.0000	52	4	8.77	3.07	1	10.295	11.81
Euderma maculatum	Myotis evotis	1.0000	52	0	-0.14	-1.41	1	11.53	9.34
Euderma maculatum	Myotis nigricans	0.3333	52	0	-0.34	-3.15	1	10.22	11.96
Euderma maculatum	Myotis occultus	0.3333	52	0	-0.05	0.58	1	11.985	8.43
Euderma maculatum	Myotis velifer	0.3333	52	2	2.95	1.83	1	12.99	6.42
Euderma maculatum	Myotis volans	0.0000	52	2	9.71	3.94	1	12.42	7.56
Euderma maculatum	Myotis yumanensis	0.5000	52	1	2.37	2.02	1	10.675	11.05
Euderma maculatum	Nycticeius humeralis	1.0000	52	0	-0.2	-2.08	1	12.655	7.09
Euderma maculatum	Nyctinomops laticaudatus	0.5000	108	0	-0.23	-2.36	1	14.6	3.2
Euderma maculatum	Nyctinomops macrotis	0.5000	108	4	15.93	4.24	1	18.5	4.6
Euderma maculatum	Parastrellus hesperus	1.0000	52	3	6.52	2.79	1	9.88	12.64
Euderma maculatum	Phyllostomus discolor	0.0000	120	0	-0.24	-2.41	0	26.35	20.3
Euderma maculatum	Pteronotus davyi	0.0000	120	0	-0.46	-3.75	1	12.84	6.72
Euderma maculatum	Pteronotus parnellii	0.0000	120	1	0.89	0.87	1	17.85	3.3
Euderma maculatum	Sturnira hondurensis	0.0000	120	1	1.75	1.59	0	18.55	4.7
Euderma maculatum	Sturnira lilium	0.0000	120	0	-0.47	-3.8	0	18.2	4
Euderma maculatum	Tadarida brasiliensis	0.1429	108	5	7.14	2.5	1	14.2	4
Euderma maculatum	Trachops cirrhosus	0.0000	120	0	-0.2	-2.12	1	26.55	20.7

A. Bats' characteristics of Known model

Euderma maculatum	Vampyrodes caraccioli	0.0000	120	0	-0.14	-1.36	0	26	19.6
Eumops auripendulus	Eumops ferox	0.5000	18.2	1	6.86	3.93	1	32.15	7.5
Eumops auripendulus	Eumops nanus	1.0000	24.6	1	14.16	5.44	1	20.25	16.3
Eumops auripendulus	Eumops perotis	1.0000	29.6	0	-0.12	-1.13	1	39.65	22.5
Eumops auripendulus	Glossophaga commissarisi	0.0000	120	1	2.71	2.23	0	18.735	19.33
Eumops auripendulus	Glossophaga soricina	0.2000	120	2	2.53	1.61	0	19.15	18.5
Eumops auripendulus	Lasionycteris noctivagans	1.0000	108	0	-0.04	1.39	1	19.65	17.5
Eumops auripendulus	Lasiurus blossevillii	1.0000	108	0	-0.32	-3.02	1	18.7	19.4
Eumops auripendulus	Lasiurus borealis	1.0000	108	0	-0.21	-2.16	1	20.35	16.1
Eumops auripendulus	Lasiurus cinereus	1.0000	108	0	-0.35	-3.19	1	27.6	1.6
Eumops auripendulus	Lasiurus ega	1.0000	108	1	4.06	2.93	1	20.25	16.3
Eumops auripendulus	Lasiurus intermedius	1.0000	108	0	-0.25	-2.57	1	25.65	5.5
Eumops auripendulus	Lasiurus xanthinus	1.0000	108	0	-0.28	-2.72	1	22.2	12.4
Eumops auripendulus	Lonchorhina aurita	0.0000	120	2	14.81	4.79	1	21.85	13.1
Eumops auripendulus	Molossus molossus	0.3333	45	1	6.76	3.89	1	21.1	14.6
Eumops auripendulus	Molossus rufus	0.5000	45	2	5.42	2.82	1	30.05	3.3
Eumops auripendulus	Myotis albescens	1.0000	108	0	-0.08	-0.31	1	17.025	22.75
Eumops auripendulus	Myotis californicus	1.0000	108	0	-0.36	-3.23	1	16.395	24.01
Eumops auripendulus	Myotis evotis	1.0000	108	0	-0.12	-1	1	17.63	21.54
Eumops auripendulus	Myotis nigricans	0.3333	108	1	3.32	2.57	1	16.32	24.16
Eumops auripendulus	Myotis occultus	0.3333	108	0	-0.04	0.99	1	18.085	20.63
Eumops auripendulus	Myotis velifer	0.3333	108	0	-0.46	-3.77	1	19.09	18.62
Eumops auripendulus	Myotis volans	0.0000	108	0	-0.16	-1.7	1	18.52	19.76
Eumops auripendulus	Myotis yumanensis	0.5000	108	0	-0.3	-2.88	1	16.775	23.25
Eumops auripendulus	Nycticeius humeralis	1.0000	108	0	-0.16	-1.67	1	18.755	19.29
Eumops auripendulus	Nyctinomops laticaudatus	0.5000	45.8	0	-0.19	-1.96	1	20.7	15.4
Eumops auripendulus	Nyctinomops macrotis	0.5000	45.8	0	-0.2	-2.1	1	24.6	7.6
Eumops auripendulus	Parastrellus hesperus	1.0000	108	0	-0.35	-3.22	1	15.98	24.84
Eumops auripendulus	Phyllostomus discolor	0.0000	120	3	15.39	4.44	0	32.45	8.1
Eumops auripendulus	Pteronotus davyi	0.0000	120	2	4.95	2.66	1	18.94	18.92
Eumops auripendulus	Pteronotus parnellii	0.0000	120	2	3.25	1.97	1	23.95	8.9
Eumops auripendulus	Sturnira hondurensis	0.0000	120	2	5.03	2.69	0	24.65	7.5
Eumops auripendulus	Sturnira lilium	0.0000	120	2	4.8	2.61	0	24.3	8.2
Eumops auripendulus	Tadarida brasiliensis	0.1429	47.2	1	1.38	1.29	1	20.3	16.2

A. Bats' characteristics of Known model

Eumops auripendulus	Trachops cirrhosus	0.0000	120	2	11.83	4.32	1	32.65	8.5
Eumops auripendulus	Vampyrodes caraccioli	0.0000	120	0	-0.11	-0.96	0	32.1	7.4
Eumops ferox	Eumops nanus	0.5000	24.6	2	9.73	4.18	1	24	23.8
Eumops ferox	Eumops perotis	0.5000	29.6	0	-0.36	-3.24	1	43.4	15
Eumops ferox	Glossophaga commissarisi	0.0000	120	13	12.85	2.75	0	22.485	26.83
Eumops ferox	Glossophaga soricina	0.1667	120	29	14.16	2.21	0	22.9	26
Eumops ferox	Lasionycteris noctivagans	0.5000	108	0	-0.1	-0.72	1	23.4	25
Eumops ferox	Lasiurus blossevillii	0.5000	108	8	7.83	2.3	1	22.45	26.9
Eumops ferox	Lasiurus borealis	0.5000	108	0	-0.6	-4.27	1	24.1	23.6
Eumops ferox	Lasiurus cinereus	0.5000	108	2	1.01	0.71	1	31.35	9.1
Eumops ferox	Lasiurus ega	0.5000	108	8	11.3	2.97	1	24	23.8
Eumops ferox	Lasiurus intermedius	0.5000	108	10	12.97	3.02	1	29.4	13
Eumops ferox	Lasiurus xanthinus	0.5000	108	3	3	1.59	1	25.95	19.9
Eumops ferox	Lonchorhina aurita	0.0000	120	4	10.04	3.44	1	25.6	20.6
Eumops ferox	Molossus molossus	0.2500	45	8	18.85	4.1	1	24.85	22.1
Eumops ferox	Molossus rufus	0.3333	45	20	19.11	3.1	1	33.8	4.2
Eumops ferox	Myotis albescens	0.5000	108	0	-0.24	-2.42	1	20.775	30.25
Eumops ferox	Myotis californicus	0.5000	108	2	0.94	0.66	1	20.145	31.51
Eumops ferox	Myotis evotis	0.5000	108	0	-0.34	-3.12	1	21.38	29.04
Eumops ferox	Myotis nigricans	0.2500	108	9	10.5	2.72	1	20.07	31.66
Eumops ferox	Myotis occultus	0.2500	108	0	-0.12	-1.13	1	21.835	28.13
Eumops ferox	Myotis velifer	0.2500	108	0	-1.34	-5.88	1	22.84	26.12
Eumops ferox	Myotis volans	0.0000	108	0	-0.47	-3.81	1	22.27	27.26
Eumops ferox	Myotis yumanensis	0.3333	108	0	-0.86	-4.99	1	20.525	30.75
Eumops ferox	Nycticeius humeralis	0.5000	108	0	-0.47	-3.79	1	22.505	26.79
Eumops ferox	Nyctinomops laticaudatus	0.3333	45.8	8	14.34	3.46	1	24.45	22.9
Eumops ferox	Nyctinomops macrotis	0.3333	45.8	6	9.79	2.97	1	28.35	15.1
Eumops ferox	Parastrellus hesperus	0.5000	108	0	-1.01	-5.33	1	19.73	32.34
Eumops ferox	Phyllostomus discolor	0.0000	120	5	8.51	2.88	0	36.2	0.6
Eumops ferox	Pteronotus davyi	0.0000	120	12	10.08	2.38	1	22.69	26.42
Eumops ferox	Pteronotus parnellii	0.0000	120	18	10.34	2.09	1	27.7	16.4
Eumops ferox	Sturnira hondurensis	0.0000	120	8	6.47	1.99	0	28.4	15
Eumops ferox	Sturnira lilium	0.0000	120	16	13.36	2.63	0	28.05	15.7
Eumops ferox	Tadarida brasiliensis	0.1250	47.2	2	-0.18	-0.13	1	24.05	23.7

A. Bats' characteristics of Known model

Eumops ferox	Trachops cirrhosus	0.0000	120	7	14.17	3.58	1	36.4	1
Eumops ferox	Vampyrodes caraccioli	0.0000	120	2	5.8	3.02	0	35.85	0.1
Eumops nanus	Eumops perotis	1.0000	29.6	0	-0.18	-1.82	1	31.5	38.8
Eumops nanus	Glossophaga commissarisi	0.0000	120	2	3.84	2.24	0	10.585	3.03
Eumops nanus	Glossophaga soricina	0.2000	120	7	6.92	2.18	0	11	2.2
Eumops nanus	Lasionycteris noctivagans	1.0000	108	0	-0.05	0.7	1	11.5	1.2
Eumops nanus	Lasiurus blossevillii	1.0000	108	1	1.76	1.6	1	10.55	3.1
Eumops nanus	Lasiurus borealis	1.0000	108	0	-0.29	-2.85	1	12.2	0.2
Eumops nanus	Lasiurus cinereus	1.0000	108	0	-0.49	-3.88	1	19.45	14.7
Eumops nanus	Lasiurus ega	1.0000	108	3	8.77	3.35	1	12.1	0
Eumops nanus	Lasiurus intermedius	1.0000	108	3	7.97	3.17	1	17.5	10.8
Eumops nanus	Lasiurus xanthinus	1.0000	108	2	4.74	2.59	1	14.05	3.9
Eumops nanus	Lonchorhina aurita	0.0000	120	1	5.1	3.37	1	13.7	3.2
Eumops nanus	Molossus molossus	0.3333	45	1	4.68	3.2	1	12.95	1.7
Eumops nanus	Molossus rufus	0.5000	45	6	11.74	3.24	1	21.9	19.6
Eumops nanus	Myotis albescens	1.0000	108	1	8.47	4.4	1	8.875	6.45
Eumops nanus	Myotis californicus	1.0000	108	0	-0.5	-3.92	1	8.245	7.71
Eumops nanus	Myotis evotis	1.0000	108	0	-0.16	-1.7	1	9.48	5.24
Eumops nanus	Myotis nigricans	0.3333	108	1	2.15	1.88	1	8.17	7.86
Eumops nanus	Myotis occultus	0.3333	108	0	-0.06	0.29	1	9.935	4.33
Eumops nanus	Myotis velifer	0.3333	108	0	-0.66	-4.46	1	10.94	2.32
Eumops nanus	Myotis volans	0.0000	108	0	-0.23	-2.39	1	10.37	3.46
Eumops nanus	Myotis yumanensis	0.5000	108	0	-0.42	-3.57	1	8.625	6.95
Eumops nanus	Nycticeius humeralis	1.0000	108	0	-0.23	-2.37	1	10.605	2.99
Eumops nanus	Nyctinomops laticaudatus	0.5000	45.8	1	3.5	2.67	1	12.55	0.9
Eumops nanus	Nyctinomops macrotis	0.5000	45.8	0	-0.29	-2.79	1	16.45	8.7
Eumops nanus	Parastrellus hesperus	1.0000	108	0	-0.5	-3.91	1	7.83	8.54
Eumops nanus	Phyllostomus discolor	0.0000	120	2	7.08	3.33	0	24.3	24.4
Eumops nanus	Pteronotus davyi	0.0000	120	3	5.12	2.38	1	10.79	2.62
Eumops nanus	Pteronotus parnellii	0.0000	120	4	4.59	1.97	1	15.8	7.4
Eumops nanus	Sturnira hondurensis	0.0000	120	1	1.39	1.3	0	16.5	8.8
Eumops nanus	Sturnira lilium	0.0000	120	3	4.95	2.32	0	16.15	8.1
Eumops nanus	Tadarida brasiliensis	0.1429	47.2	0	-0.74	-4.71	1	12.15	0.1
Eumops nanus	Trachops cirrhosus	0.0000	120	1	4.01	2.91	1	24.5	24.8

A. Bats' characteristics of Known model

Eumops nanus	Vampyrodes caraccioli	0.0000	120	0	-0.16	-1.65	0	23.95	23.7
Eumops perotis	Glossophaga commissarisi	0.0000	120	1	0.4	0.4	0	29.985	41.83
Eumops perotis	Glossophaga soricina	0.2000	120	6	2.21	0.88	0	30.4	41
Eumops perotis	Lasionycteris noctivagans	1.0000	108	1	11.31	5.55	1	30.9	40
Eumops perotis	Lasiurus blossevillii	1.0000	108	6	6.73	2.28	1	29.95	41.9
Eumops perotis	Lasiurus borealis	1.0000	108	3	5.26	2.45	1	31.6	38.6
Eumops perotis	Lasiurus cinereus	1.0000	108	7	7.21	2.26	1	38.85	24.1
Eumops perotis	Lasiurus ega	1.0000	108	1	1.13	1.09	1	31.5	38.8
Eumops perotis	Lasiurus intermedius	1.0000	108	2	2.51	1.61	1	36.9	28
Eumops perotis	Lasiurus xanthinus	1.0000	108	4	5.12	2.16	1	33.45	34.9
Eumops perotis	Lonchorhina aurita	0.0000	120	0	-0.34	-3.11	1	33.1	35.6
Eumops perotis	Molossus molossus	0.3333	45	1	2.4	2.06	1	32.35	37.1
Eumops perotis	Molossus rufus	0.5000	45	3	2.59	1.4	1	41.3	19.2
Eumops perotis	Myotis albescens	1.0000	108	0	-0.21	-2.14	1	28.275	45.25
Eumops perotis	Myotis californicus	1.0000	108	8	8.14	2.35	1	27.645	46.51
Eumops perotis	Myotis evotis	1.0000	108	1	3.15	2.57	1	28.88	44.04
Eumops perotis	Myotis nigricans	0.3333	108	2	2.19	1.44	1	27.57	46.66
Eumops perotis	Myotis occultus	0.3333	108	0	-0.11	-0.85	1	29.335	43.13
Eumops perotis	Myotis velifer	0.3333	108	7	4.88	1.66	1	30.34	41.12
Eumops perotis	Myotis volans	0.0000	108	1	2.02	1.8	1	29.77	42.26
Eumops perotis	Myotis yumanensis	0.5000	108	6	7.31	2.42	1	28.025	45.75
Eumops perotis	Nycticeius humeralis	1.0000	108	2	4.51	2.53	1	30.005	41.79
Eumops perotis	Nyctinomops laticaudatus	0.5000	45.8	3	5.94	2.66	1	31.95	37.9
Eumops perotis	Nyctinomops macrotis	0.5000	45.8	5	9.42	3.05	1	35.85	30.1
Eumops perotis	Parastrellus hesperus	1.0000	108	8	8.21	2.37	1	27.23	47.34
Eumops perotis	Phyllostomus discolor	0.0000	120	0	-0.48	-3.84	0	43.7	14.4
Eumops perotis	Pteronotus davyi	0.0000	120	1	0.13	0.13	1	30.19	41.42
Eumops perotis	Pteronotus parnellii	0.0000	120	7	3.97	1.4	1	35.2	31.4
Eumops perotis	Sturnira hondurensis	0.0000	120	6	5.56	1.97	0	35.9	30
Eumops perotis	Sturnira lilium	0.0000	120	2	1.11	0.77	0	35.55	30.7
Eumops perotis	Tadarida brasiliensis	0.1429	47.2	20	13.95	2.49	1	31.55	38.7
Eumops perotis	Trachops cirrhosus	0.0000	120	0	-0.42	-3.55	1	43.9	14
Eumops perotis	Vampyrodes caraccioli	0.0000	120	0	-0.29	-2.79	0	43.35	15.1
Glossophaga commissarisi	Glossophaga soricina	0.0000	13	140	29.64	2.29	1	9.485	0.83

A. Bats' characteristics of Known model

Glossophaga commissarisi	Lasionycteris noctivagans	0.0000	120	0	-0.24	-2.41	0	9.985	1.83
Glossophaga commissarisi	Lasiurus blossevillii	0.0000	120	25	9.86	1.86	0	9.035	0.07
Glossophaga commissarisi	Lasiurus borealis	0.0000	120	6	3.03	1.22	0	10.685	3.23
Glossophaga commissarisi	Lasiurus cinereus	0.0000	120	4	-0.56	-0.28	0	17.935	17.73
Glossophaga commissarisi	Lasiurus ega	0.0000	120	18	10.27	2.23	0	10.585	3.03
Glossophaga commissarisi	Lasiurus intermedius	0.0000	120	26	13.94	2.47	0	15.985	13.83
Glossophaga commissarisi	Lasiurus xanthinus	0.0000	120	12	4.82	1.36	0	12.535	6.93
Glossophaga commissarisi	Lonchorhina aurita	0.0000	55.6	13	13.99	3.38	0	12.185	6.23
Glossophaga commissarisi	Molossus molossus	0.0000	120	15	14.89	3.35	0	11.435	4.73
Glossophaga commissarisi	Molossus rufus	0.0000	120	47	18.42	2.44	0	20.385	22.63
Glossophaga commissarisi	Myotis albescens	0.0000	120	5	8.74	3.4	0	7.36	3.42
Glossophaga commissarisi	Myotis californicus	0.0000	120	3	-1.09	-0.62	0	6.73	4.68
Glossophaga commissarisi	Myotis evotis	0.0000	120	0	-0.78	-4.81	0	7.965	2.21
Glossophaga commissarisi	Myotis nigricans	0.0000	120	40	20.2	2.83	0	6.655	4.83
Glossophaga commissarisi	Myotis occultus	0.0000	120	0	-0.29	-2.82	0	8.42	1.3
Glossophaga commissarisi	Myotis velifer	0.0000	120	14	1.51	0.41	0	9.425	0.71
Glossophaga commissarisi	Myotis volans	0.0000	120	0	-1.11	-5.5	0	8.855	0.43
Glossophaga commissarisi	Myotis yumanensis	0.0000	120	4	0.05	0.03	0	7.11	3.92
Glossophaga commissarisi	Nycticeius humeralis	0.0000	120	0	-1.09	-5.48	0	9.09	0.04
Glossophaga commissarisi	Nyctinomops laticaudatus	0.0000	120	8	5.27	1.77	0	11.035	3.93
Glossophaga commissarisi	Nyctinomops macrotis	0.5000	120	11	6.99	1.97	0	14.935	11.73
Glossophaga commissarisi	Parastrellus hesperus	0.0000	120	6	0.25	0.1	0	6.315	5.51
Glossophaga commissarisi	Phyllostomus discolor	0.0000	55.6	43	32.94	4.51	1	22.785	27.43
Glossophaga commissarisi	Pteronotus davyi	0.0000	85	51	18.31	2.34	0	9.275	0.41
Glossophaga commissarisi	Pteronotus parnellii	0.0000	85	99	25.1	2.31	0	14.285	10.43
Glossophaga commissarisi	Sturnira hondurensis	1.0000	53.4	50	18.21	2.35	0	14.985	11.83
Glossophaga commissarisi	Sturnira lilium	0.2000	53.4	70	25.21	2.69	1	14.635	11.13
Glossophaga commissarisi	Tadarida brasiliensis	0.0000	120	11	-0.31	-0.09	0	10.635	3.13
Glossophaga commissarisi	Trachops cirrhosus	0.5000	55.6	24	20.94	3.72	0	22.985	27.83
Glossophaga commissarisi	Vampyrodes caraccioli	0.0000	53.4	13	16.73	4.07	0	22.435	26.73
Glossophaga soricina	Lasionycteris noctivagans	0.2000	120	1	1.88	2.19	0	10.4	1
Glossophaga soricina	Lasiurus blossevillii	0.2000	120	77	15.67	2.06	0	9.45	0.9
Glossophaga soricina	Lasiurus borealis	0.2000	120	25	7.16	1.61	0	11.1	2.4
Glossophaga soricina	Lasiurus cinereus	0.2000	120	38	4.4	0.78	0	18.35	16.9

A. Bats' characteristics of Known model

Glossophaga soricina	Lasiurus ega	0.2000	120	52	15.32	2.56	0	11	2.2
Glossophaga soricina	Lasiurus intermedius	0.2000	120	67	18.35	2.76	0	16.4	13
Glossophaga soricina	Lasiurus xanthinus	0.2000	120	45	9.81	1.64	0	12.95	6.1
Glossophaga soricina	Lonchorhina aurita	0.0000	55.6	24	13.04	3.76	0	12.6	5.4
Glossophaga soricina	Molossus molossus	0.3333	120	23	11.19	2.93	0	11.85	3.9
Glossophaga soricina	Molossus rufus	0.1667	120	140	28.72	3.13	0	20.8	21.8
Glossophaga soricina	Myotis albescens	0.2000	120	8	6.92	3.17	0	7.775	4.25
Glossophaga soricina	Myotis californicus	0.2000	120	39	4.32	0.75	0	7.145	5.51
Glossophaga soricina	Myotis evotis	0.2000	120	0	-1.57	-6.2	0	8.38	3.04
Glossophaga soricina	Myotis nigricans	0.3333	120	94	24.26	3.3	0	7.07	5.66
Glossophaga soricina	Myotis occultus	0.1429	120	1	1.34	1.5	0	8.835	2.13
Glossophaga soricina	Myotis velifer	0.1429	120	80	8.02	0.98	0	9.84	0.12
Glossophaga soricina	Myotis volans	0.0000	120	4	-0.21	-0.11	0	9.27	1.26
Glossophaga soricina	Myotis yumanensis	0.1667	120	32	4.87	0.94	0	7.525	4.75
Glossophaga soricina	Nycticeius humeralis	0.2000	120	6	0.86	0.38	0	9.505	0.79
Glossophaga soricina	Nyctinomops laticaudatus	0.1667	120	40	15.12	3.05	0	11.45	3.1
Glossophaga soricina	Nyctinomops macrotis	0.1667	120	33	10.81	2.19	0	15.35	10.9
Glossophaga soricina	Parastrellus hesperus	0.2000	120	33	3.02	0.57	0	6.73	6.34
Glossophaga soricina	Phyllostomus discolor	0.0000	55.6	48	18.05	3.58	1	23.2	26.6
Glossophaga soricina	Pteronotus davyi	0.0000	85	147	27.37	2.79	0	9.69	0.42
Glossophaga soricina	Pteronotus parnellii	0.1000	85	318	42.58	3.04	0	14.7	9.6
Glossophaga soricina	Sturnira hondurensis	0.0000	53.4	124	22.74	2.43	0	15.4	11
Glossophaga soricina	Sturnira lilium	0.0000	53.4	175	32.35	3.17	1	15.05	10.3
Glossophaga soricina	Tadarida brasiliensis	0.0909	120	94	7.77	0.87	0	11.05	2.3
Glossophaga soricina	Trachops cirrhosus	0.0000	55.6	34	14.64	3.32	0	23.4	27
Glossophaga soricina	Vampyrodes caraccioli	0.0000	53.4	15	9.37	3.11	0	22.85	25.9
Lasionycteris noctivagans	Lasiurus blossevillii	1.0000	52	1	4.2	2.98	1	9.95	1.9
Lasionycteris noctivagans	Lasiurus borealis	1.0000	52	1	6.65	3.85	1	11.6	1.4
Lasionycteris noctivagans	Lasiurus cinereus	1.0000	52	1	3.83	2.81	1	18.85	15.9
Lasionycteris noctivagans	Lasiurus ega	1.0000	52	2	11.97	4.32	1	11.5	1.2
Lasionycteris noctivagans	Lasiurus intermedius	1.0000	52	1	5.37	3.44	1	16.9	12
Lasionycteris noctivagans	Lasiurus xanthinus	1.0000	52	0	-0.19	-2.03	1	13.45	5.1
Lasionycteris noctivagans	Lonchorhina aurita	0.0000	120	0	-0.09	-0.59	1	13.1	4.4
Lasionycteris noctivagans	Molossus molossus	0.3333	108	0	-0.1	-0.75	1	12.35	2.9

A. Bats' characteristics of Known model

Lasionycteris noctivagans	Molossus rufus	0.5000	108	0	-0.25	-2.49	1	21.3	20.8
Lasionycteris noctivagans	Myotis albescens	1.0000	52	0	-0.06	0.38	1	8.275	5.25
Lasionycteris noctivagans	Myotis californicus	1.0000	52	1	3.73	2.77	1	7.645	6.51
Lasionycteris noctivagans	Myotis evotis	1.0000	52	0	-0.08	-0.31	1	8.88	4.04
Lasionycteris noctivagans	Myotis nigricans	0.3333	52	0	-0.2	-2.05	1	7.57	6.66
Lasionycteris noctivagans	Myotis occultus	0.3333	52	0	-0.03	1.68	1	9.335	3.13
Lasionycteris noctivagans	Myotis velifer	0.3333	52	1	2.72	2.23	1	10.34	1.12
Lasionycteris noctivagans	Myotis volans	0.0000	52	0	-0.12	-1.01	1	9.77	2.26
Lasionycteris noctivagans	Myotis yumanensis	0.5000	52	0	-0.21	-2.19	1	8.025	5.75
Lasionycteris noctivagans	Nycticeius humeralis	1.0000	52	0	-0.12	-0.98	1	10.005	1.79
Lasionycteris noctivagans	Nyctinomops laticaudatus	0.5000	108	1	7.4	4.06	1	11.95	2.1
Lasionycteris noctivagans	Nyctinomops macrotis	0.5000	108	0	-0.14	-1.41	1	15.85	9.9
Lasionycteris noctivagans	Parastrellus hesperus	1.0000	52	1	3.76	2.78	1	7.23	7.34
Lasionycteris noctivagans	Phyllostomus discolor	0.0000	120	0	-0.14	-1.32	0	23.7	25.6
Lasionycteris noctivagans	Pteronotus davyi	0.0000	120	0	-0.27	-2.65	1	10.19	1.42
Lasionycteris noctivagans	Pteronotus parnellii	0.0000	120	1	2.3	1.97	1	15.2	8.6
Lasionycteris noctivagans	Sturnira hondurensis	0.0000	120	0	-0.26	-2.62	0	15.9	10
Lasionycteris noctivagans	Sturnira lilium	0.0000	120	0	-0.27	-2.71	0	15.55	9.3
Lasionycteris noctivagans	Tadarida brasiliensis	0.1429	108	2	5.02	2.68	1	11.55	1.3
Lasionycteris noctivagans	Trachops cirrhosus	0.0000	120	0	-0.12	-1.03	1	23.9	26
Lasionycteris noctivagans	Vampyrodes caraccioli	0.0000	120	0	-0.08	-0.27	0	23.35	24.9
Lasiurus blossevillii	Lasiurus borealis	1.0000	6	32	22.91	3.47	1	10.65	3.3
Lasiurus blossevillii	Lasiurus cinereus	1.0000	16.2	48	19.55	2.52	1	17.9	17.8
Lasiurus blossevillii	Lasiurus ega	1.0000	22.2	21	12.69	2.48	1	10.55	3.1
Lasiurus blossevillii	Lasiurus intermedius	1.0000	22.2	42	24.35	3.24	1	15.95	13.9
Lasiurus blossevillii	Lasiurus xanthinus	1.0000	22.2	35	18.23	2.72	1	12.5	7
Lasiurus blossevillii	Lonchorhina aurita	0.0000	120	6	6.2	2.3	1	12.15	6.3
Lasiurus blossevillii	Molossus molossus	0.3333	108	9	8.85	2.62	1	11.4	4.8
Lasiurus blossevillii	Molossus rufus	0.5000	108	33	12.74	2.05	1	20.35	22.7
Lasiurus blossevillii	Myotis albescens	1.0000	52	3	5.2	2.66	1	7.325	3.35
Lasiurus blossevillii	Myotis californicus	1.0000	52	44	17.24	2.35	1	6.695	4.61
Lasiurus blossevillii	Myotis evotis	1.0000	52	4	4.65	2.14	1	7.93	2.14
Lasiurus blossevillii	Myotis nigricans	0.3333	52	31	15.78	2.53	1	6.62	4.76
Lasiurus blossevillii	Myotis occultus	0.3333	52	0	-0.28	-2.76	1	8.385	1.23

A. Bats' characteristics of Known model

Lasiurus blossevillii	Myotis velifer	0.3333	52	64	18.71	2.15	1	9.39	0.78
Lasiurus blossevillii	Myotis volans	0.0000	52	10	8.49	2.42	1	8.82	0.36
Lasiurus blossevillii	Myotis yumanensis	0.5000	52	22	9.69	1.93	1	7.075	3.85
Lasiurus blossevillii	Nycticeius humeralis	1.0000	52	9	7.65	2.31	1	9.055	0.11
Lasiurus blossevillii	Nyctinomops laticaudatus	0.5000	108	18	13.91	2.87	1	11	4
Lasiurus blossevillii	Nyctinomops macrotis	0.5000	108	19	13.52	2.74	1	14.9	11.8
Lasiurus blossevillii	Parastrellus hesperus	1.0000	52	34	12.92	2.05	1	6.28	5.44
Lasiurus blossevillii	Phyllostomus discolor	0.0000	120	8	5.3	1.77	0	22.75	27.5
Lasiurus blossevillii	Pteronotus davyi	0.0000	120	32	11	1.83	1	9.24	0.48
Lasiurus blossevillii	Pteronotus parnellii	0.0000	120	77	19.48	2.06	1	14.25	10.5
Lasiurus blossevillii	Sturnira hondurensis	0.0000	120	41	15.05	2.16	0	14.95	11.9
Lasiurus blossevillii	Sturnira lilium	0.0000	120	38	13.02	1.97	0	14.6	11.2
Lasiurus blossevillii	Tadarida brasiliensis	0.1429	108	66	16.41	1.89	1	10.6	3.2
Lasiurus blossevillii	Trachops cirrhosus	0.0000	120	5	3.64	1.57	1	22.95	27.9
Lasiurus blossevillii	Vampyrodes caraccioli	0.0000	120	1	0.64	0.65	0	22.4	26.8
Lasiurus borealis	Lasiurus cinereus	1.0000	16.2	23	14.46	2.5	1	19.55	14.5
Lasiurus borealis	Lasiurus ega	1.0000	22.2	12	11.39	2.67	1	12.2	0.2
Lasiurus borealis	Lasiurus intermedius	1.0000	22.2	17	14.97	2.87	1	17.6	10.6
Lasiurus borealis	Lasiurus xanthinus	1.0000	22.2	12	9.3	2.29	1	14.15	3.7
Lasiurus borealis	Lonchorhina aurita	0.0000	120	1	1.23	1.19	1	13.8	3
Lasiurus borealis	Molossus molossus	0.3333	108	2	2.71	1.75	1	13.05	1.5
Lasiurus borealis	Molossus rufus	0.5000	108	8	4.08	1.36	1	22	19.4
Lasiurus borealis	Myotis albescens	1.0000	52	1	2.57	2.22	1	8.975	6.65
Lasiurus borealis	Myotis californicus	1.0000	52	18	10.69	2.18	1	8.345	7.91
Lasiurus borealis	Myotis evotis	1.0000	52	3	5.69	2.18	1	9.58	5.44
Lasiurus borealis	Myotis nigricans	0.3333	52	10	7.48	2.07	1	8.27	8.06
Lasiurus borealis	Myotis occultus	0.3333	52	0	-0.18	-1.88	1	10.035	4.53
Lasiurus borealis	Myotis velifer	0.3333	52	26	11.5	2	1	11.04	2.52
Lasiurus borealis	Myotis volans	0.0000	52	0	-0.69	-4.57	1	10.47	3.66
Lasiurus borealis	Myotis yumanensis	0.5000	52	8	5.19	1.68	1	8.725	7.15
Lasiurus borealis	Nycticeius humeralis	1.0000	52	8	11.11	3.04	1	10.705	3.19
Lasiurus borealis	Nyctinomops laticaudatus	0.5000	108	9	10.74	2.84	1	12.65	0.7
Lasiurus borealis	Nyctinomops macrotis	0.5000	108	9	9.86	2.67	1	16.55	8.5
Lasiurus borealis	Parastrellus hesperus	1.0000	52	17	10.11	2.13	1	7.93	8.74

A. Bats' characteristics of Known model

Lasiurus borealis	Phyllostomus discolor	0.0000	120	1	0.44	0.44	0	24.4	24.2
Lasiurus borealis	Pteronotus davyi	0.0000	120	12	6.11	1.63	1	10.89	2.82
Lasiurus borealis	Pteronotus parnellii	0.0000	120	26	9.57	1.72	1	15.9	7.2
Lasiurus borealis	Sturnira hondurensis	0.0000	120	15	8.18	1.89	0	16.6	8.6
Lasiurus borealis	Sturnira lilium	0.0000	120	14	7.1	1.73	0	16.25	7.9
Lasiurus borealis	Tadarida brasiliensis	0.1429	108	29	11.07	1.85	1	12.25	0.1
Lasiurus borealis	Trachops cirrhosus	0.0000	120	1	0.74	0.74	1	24.6	24.6
Lasiurus borealis	Vampyrodes caraccioli	0.0000	120	0	-0.48	-3.83	0	24.05	23.5
Lasiurus cinereus	Lasiurus ega	1.0000	22.2	13	6.45	1.72	1	19.45	14.7
Lasiurus cinereus	Lasiurus intermedius	1.0000	22.2	35	18.18	2.78	1	24.85	3.9
Lasiurus cinereus	Lasiurus xanthinus	1.0000	22.2	24	10.71	2.05	1	21.4	10.8
Lasiurus cinereus	Lonchorhina aurita	0.0000	120	1	0.14	0.14	1	21.05	11.5
Lasiurus cinereus	Molossus molossus	0.3333	108	4	2.99	1.46	1	20.3	13
Lasiurus cinereus	Molossus rufus	0.5000	108	13	2.99	0.83	1	29.25	4.9
Lasiurus cinereus	Myotis albescens	1.0000	52	0	-0.58	-4.22	1	16.225	21.15
Lasiurus cinereus	Myotis californicus	1.0000	52	47	16.73	2.27	1	15.595	22.41
Lasiurus cinereus	Myotis evotis	1.0000	52	9	10.4	3.1	1	16.83	19.94
Lasiurus cinereus	Myotis nigricans	0.3333	52	11	3.79	1.13	1	15.52	22.56
Lasiurus cinereus	Myotis occultus	0.3333	52	2	6.45	4.16	1	17.285	19.03
Lasiurus cinereus	Myotis velifer	0.3333	52	71	18.94	2.11	1	18.29	17.02
Lasiurus cinereus	Myotis volans	0.0000	52	17	13.82	3.01	1	17.72	18.16
Lasiurus cinereus	Myotis yumanensis	0.5000	52	32	13.49	2.22	1	15.975	21.65
Lasiurus cinereus	Nycticeius humeralis	1.0000	52	11	8.66	2.4	1	17.955	17.69
Lasiurus cinereus	Nyctinomops laticaudatus	0.5000	108	5	2.54	1.13	1	19.9	13.8
Lasiurus cinereus	Nyctinomops macrotis	0.5000	108	27	18.01	3.1	1	23.8	6
Lasiurus cinereus	Parastrellus hesperus	1.0000	52	48	17.31	2.31	1	15.18	23.24
Lasiurus cinereus	Phyllostomus discolor	0.0000	120	1	-0.61	-0.6	0	31.65	9.7
Lasiurus cinereus	Pteronotus davyi	0.0000	120	17	3.93	0.95	1	18.14	17.32
Lasiurus cinereus	Pteronotus parnellii	0.0000	120	39	6.95	1.11	1	23.15	7.3
Lasiurus cinereus	Sturnira hondurensis	0.0000	120	38	12.29	1.89	0	23.85	5.9
Lasiurus cinereus	Sturnira lilium	0.0000	120	20	4.8	1.07	0	23.5	6.6
Lasiurus cinereus	Tadarida brasiliensis	0.1429	108	104	25.09	2.28	1	19.5	14.6
Lasiurus cinereus	Trachops cirrhosus	0.0000	120	1	-0.31	-0.31	1	31.85	10.1
Lasiurus cinereus	Vampyrodes caraccioli	0.0000	120	0	-0.81	-4.87	0	31.3	9

A. Bats' characteristics of Known model

Lasiurus ega	Lasiurus intermedius	1.0000	9.4	37	29.98	3.68	1	17.5	10.8
Lasiurus ega	Lasiurus xanthinus	1.0000	9.4	28	20.5	3.06	1	14.05	3.9
Lasiurus ega	Lonchorhina aurita	0.0000	120	6	8.99	2.94	1	13.7	3.2
Lasiurus ega	Molossus molossus	0.3333	108	6	8.2	2.74	1	12.95	1.7
Lasiurus ega	Molossus rufus	0.5000	108	26	14.44	2.41	1	21.9	19.6
Lasiurus ega	Myotis albescens	1.0000	52	0	-0.39	-3.41	1	8.875	6.45
Lasiurus ega	Myotis californicus	1.0000	52	11	4.97	1.42	1	8.245	7.71
Lasiurus ega	Myotis evotis	1.0000	52	3	4.97	2.44	1	9.48	5.24
Lasiurus ega	Myotis nigricans	0.3333	52	17	11.82	2.44	1	8.17	7.86
Lasiurus ega	Myotis occultus	0.3333	52	0	-0.2	-2.11	1	9.935	4.33
Lasiurus ega	Myotis velifer	0.3333	52	19	6.59	1.43	1	10.94	2.32
Lasiurus ega	Myotis volans	0.0000	52	4	4.43	1.98	1	10.37	3.46
Lasiurus ega	Myotis yumanensis	0.5000	52	7	3.63	1.31	1	8.625	6.95
Lasiurus ega	Nycticeius humeralis	1.0000	52	9	11.08	2.96	1	10.605	2.99
Lasiurus ega	Nyctinomops laticaudatus	0.5000	108	13	13.98	3.07	1	12.55	0.9
Lasiurus ega	Nyctinomops macrotis	0.5000	108	9	8.61	2.44	1	16.45	8.7
Lasiurus ega	Parastrellus hesperus	1.0000	52	11	5.04	1.44	1	7.83	8.54
Lasiurus ega	Phyllostomus discolor	0.0000	120	11	11.35	2.79	0	24.3	24.4
Lasiurus ega	Pteronotus davyi	0.0000	120	2	13.1	2.23	1	10.79	2.62
Lasiurus ega	Pteronotus parnellii	0.0000	120	44	15.33	2.05	1	15.8	7.4
Lasiurus ega	Sturnira hondurensis	0.0000	120	14	6.36	1.59	0	16.5	8.8
Lasiurus ega	Sturnira lilium	0.0000	120	31	15.42	2.37	0	16.15	8.1
Lasiurus ega	Tadarida brasiliensis	0.1429	108	22	6.52	1.33	1	12.15	0.1
Lasiurus ega	Trachops cirrhosus	0.0000	120	9	10.8	2.9	1	24.5	24.8
Lasiurus ega	Vampyrodes caraccioli	0.0000	120	2	3.23	2.04	0	23.95	23.7
Lasiurus intermedius	Lasiurus xanthinus	1.0000	9.4	27	17.85	2.84	1	19.45	6.9
Lasiurus intermedius	Lonchorhina aurita	0.0000	120	3	3.72	1.95	1	19.1	7.6
Lasiurus intermedius	Molossus molossus	0.3333	108	8	10.11	2.93	1	18.35	9.1
Lasiurus intermedius	Molossus rufus	0.5000	108	34	17.48	2.55	1	27.3	8.8
Lasiurus intermedius	Myotis albescens	1.0000	52	2	4.35	2.6	1	14.275	17.25
Lasiurus intermedius	Myotis californicus	1.0000	52	18	8.14	1.77	1	13.645	18.51
Lasiurus intermedius	Myotis evotis	1.0000	52	1	1.09	1.07	1	14.88	16.04
Lasiurus intermedius	Myotis nigricans	0.3333	52	21	13.43	2.51	1	13.57	18.66
Lasiurus intermedius	Myotis occultus	0.3333	52	0	-0.22	-2.29	1	15.335	15.13

A. Bats' characteristics of Known model

Lasiurus intermedius	Myotis velifer	0.3333	52	35	12.43	1.91	1	16.34	13.12
Lasiurus intermedius	Myotis volans	0.0000	52	3	2.73	1.49	1	15.77	14.26
Lasiurus intermedius	Myotis yumanensis	0.5000	52	5	1.76	0.78	1	14.025	17.75
Lasiurus intermedius	Nycticeius humeralis	1.0000	52	11	12.44	3.04	1	16.005	13.79
Lasiurus intermedius	Nyctinomops laticaudatus	0.5000	108	16	15.81	3.16	1	17.95	9.9
Lasiurus intermedius	Nyctinomops macrotis	0.5000	108	14	12.6	2.79	1	21.85	2.1
Lasiurus intermedius	Parastrellus hesperus	1.0000	52	8	2.65	0.92	1	13.23	19.34
Lasiurus intermedius	Phyllostomus discolor	0.0000	120	11	10.25	2.61	0	29.7	13.6
Lasiurus intermedius	Pteronotus davyi	0.0000	120	39	18.51	2.53	1	16.19	13.42
Lasiurus intermedius	Pteronotus parnellii	0.0000	120	61	19.93	2.24	1	21.2	3.4
Lasiurus intermedius	Sturnira hondurensis	0.0000	120	25	11.37	2.04	0	21.9	2
Lasiurus intermedius	Sturnira lilium	0.0000	120	35	15.85	2.33	0	21.55	2.7
Lasiurus intermedius	Tadarida brasiliensis	0.1429	108	36	10.78	1.67	1	17.55	10.7
Lasiurus intermedius	Trachops cirrhosus	0.0000	120	8	8.58	2.57	1	29.9	14
Lasiurus intermedius	Vampyrodes caraccioli	0.0000	120	2	2.87	1.86	0	29.35	12.9
Lasiurus xanthinus	Lonchorhina aurita	0.0000	120	1	0.61	0.62	1	15.65	0.7
Lasiurus xanthinus	Molossus molossus	0.3333	108	5	5.47	2.19	1	14.9	2.2
Lasiurus xanthinus	Molossus rufus	0.5000	108	21	9.07	1.83	1	23.85	15.7
Lasiurus xanthinus	Myotis albescens	1.0000	52	0	-0.46	-3.75	1	10.825	10.35
Lasiurus xanthinus	Myotis californicus	1.0000	52	27	11.86	2.06	1	10.195	11.61
Lasiurus xanthinus	Myotis evotis	1.0000	52	6	8.72	2.96	1	11.43	9.14
Lasiurus xanthinus	Myotis nigricans	0.3333	52	11	5.65	1.61	1	10.12	11.76
Lasiurus xanthinus	Myotis occultus	0.3333	52	0	-0.24	-2.45	1	11.885	8.23
Lasiurus xanthinus	Myotis velifer	0.3333	52	38	12.3	1.84	1	12.89	6.22
Lasiurus xanthinus	Myotis volans	0.0000	52	9	9.01	2.59	1	12.32	7.36
Lasiurus xanthinus	Myotis yumanensis	0.5000	52	20	10.54	2.12	1	10.575	10.85
Lasiurus xanthinus	Nycticeius humeralis	1.0000	52	8	8.02	2.47	1	12.555	6.89
Lasiurus xanthinus	Nyctinomops laticaudatus	0.5000	108	5	3.8	1.6	1	14.5	3
Lasiurus xanthinus	Nyctinomops macrotis	0.5000	108	14	11.49	2.63	1	18.4	4.8
Lasiurus xanthinus	Parastrellus hesperus	1.0000	52	28	12.49	2.12	1	9.78	12.44
Lasiurus xanthinus	Phyllostomus discolor	0.0000	120	4	2.7	1.31	0	26.25	20.5
Lasiurus xanthinus	Pteronotus davyi	0.0000	120	20	7.6	1.6	1	12.74	6.52
Lasiurus xanthinus	Pteronotus parnellii	0.0000	120	34	8.72	1.43	1	17.75	3.5
Lasiurus xanthinus	Sturnira hondurensis	0.0000	120	13	4.32	1.17	0	18.45	4.9

A. Bats' characteristics of Known model

Lasiurus xanthinus	Sturnira lilium	0.0000	120	20	7.28	1.54	0	18.1	4.2
Lasiurus xanthinus	Tadarida brasiliensis	0.1429	108	51	14.75	1.9	1	14.1	3.8
Lasiurus xanthinus	Trachops cirrhosus	0.0000	120	2	1.25	0.88	1	26.45	20.9
Lasiurus xanthinus	Vampyrodes caraccioli	0.0000	120	1	0.96	0.95	0	25.9	19.8
Lonchorhina aurita	Molossus molossus	0.0000	120	3	7.31	3.07	1	14.55	1.5
Lonchorhina aurita	Molossus rufus	0.0000	120	10	9.79	2.49	1	23.5	16.4
Lonchorhina aurita	Myotis albescens	0.0000	120	2	8.81	3.9	1	10.475	9.65
Lonchorhina aurita	Myotis californicus	0.0000	120	1	0.09	0.1	1	9.845	10.91
Lonchorhina aurita	Myotis evotis	0.0000	120	0	-0.31	-2.99	1	11.08	8.44
Lonchorhina aurita	Myotis nigricans	0.0000	120	5	5.95	2.23	1	9.77	11.06
Lonchorhina aurita	Myotis occultus	0.0000	120	0	-0.12	-1	1	11.535	7.53
Lonchorhina aurita	Myotis velifer	0.0000	120	2	0.35	0.25	1	12.54	5.52
Lonchorhina aurita	Myotis volans	0.0000	120	1	1.81	1.65	1	11.97	6.66
Lonchorhina aurita	Myotis yumanensis	0.0000	120	0	-0.81	-4.86	1	10.225	10.15
Lonchorhina aurita	Nycticeius humeralis	0.0000	120	0	-0.44	-3.66	1	12.205	6.19
Lonchorhina aurita	Nyctinomops laticaudatus	0.0000	120	3	5.44	2.51	1	14.15	2.3
Lonchorhina aurita	Nyctinomops macrotis	0.0000	120	0	-0.54	-4.08	1	18.05	5.5
Lonchorhina aurita	Parastrellus hesperus	0.0000	120	0	-0.95	-5.2	1	9.43	11.74
Lonchorhina aurita	Phyllostomus discolor	0.0000	55.2	11	20.74	3.91	0	25.9	21.2
Lonchorhina aurita	Pteronotus davyi	0.0000	85	9	7.91	2.21	1	12.39	5.82
Lonchorhina aurita	Pteronotus parnellii	0.0000	85	20	12.63	2.33	1	17.4	4.2
Lonchorhina aurita	Sturnira hondurensis	0.0000	55.6	6	5.03	1.82	0	18.1	5.6
Lonchorhina aurita	Sturnira lilium	0.0000	55.6	18	16.32	2.89	0	17.75	4.9
Lonchorhina aurita	Tadarida brasiliensis	0.0000	120	2	0	0	1	13.75	3.1
Lonchorhina aurita	Trachops cirrhosus	0.0000	55.2	11	24.1	4.28	1	26.1	21.6
Lonchorhina aurita	Vampyrodes caraccioli	0.0000	55.6	5	16.03	4.24	0	25.55	20.5
Molossus molossus	Molossus rufus	0.2500	44	21	19.79	3.13	1	22.75	17.9
Molossus molossus	Myotis albescens	0.3333	108	0	-0.24	-2.45	1	9.725	8.15
Molossus molossus	Myotis californicus	0.3333	108	3	1.87	1.04	1	9.095	9.41
Molossus molossus	Myotis evotis	0.3333	108	0	-0.34	-3.15	1	10.33	6.94
Molossus molossus	Myotis nigricans	0.2000	108	12	14.04	3	1	9.02	9.56
Molossus molossus	Myotis occultus	0.2000	108	0	-0.13	-1.16	1	10.785	6.03
Molossus molossus	Myotis velifer	0.2000	108	8	4.57	1.49	1	11.79	4.02
Molossus molossus	Myotis volans	0.0000	108	0	-0.48	-3.84	1	11.22	5.16

A. Bats' characteristics of Known model

Molossus molossus	Myotis yumanensis	0.2500	108	1	0.28	0.29	1	9.475	8.65
Molossus molossus	Nycticeius humeralis	0.3333	108	0	-0.48	-3.82	1	11.455	4.69
Molossus molossus	Nyctinomops laticaudatus	0.2500	45.8	9	15.94	3.57	1	13.4	0.8
Molossus molossus	Nyctinomops macrotis	0.2500	45.8	4	6.22	2.5	1	17.3	7
Molossus molossus	Parastrellus hesperus	0.3333	108	2	0.92	0.65	1	8.68	10.24
Molossus molossus	Phyllostomus discolor	0.0000	120	9	15.51	3.51	0	25.15	22.7
Molossus molossus	Pteronotus davyi	0.0000	120	8	6.23	1.93	1	11.64	4.32
Molossus molossus	Pteronotus parnellii	0.0000	120	21	12.09	2.22	1	16.65	5.7
Molossus molossus	Sturnira hondurensis	0.0000	120	9	7.27	2.08	0	17.35	7.1
Molossus molossus	Sturnira lilium	0.0000	120	15	12.24	2.53	0	17	6.4
Molossus molossus	Tadarida brasiliensis	0.1111	47.2	7	3.05	1.11	1	13	1.6
Molossus molossus	Trachops cirrhosus	0.0000	120	6	11.89	3.37	1	25.35	23.1
Molossus molossus	Vampyrodes caraccioli	0.0000	120	0	-0.33	-3.1	0	24.8	22
Molossus rufus	Myotis albescens	0.5000	108	4	6.47	2.91	1	18.675	26.05
Molossus rufus	Myotis californicus	0.5000	108	8	0.76	0.27	1	18.045	27.31
Molossus rufus	Myotis evotis	0.5000	108	0	-0.83	-4.92	1	19.28	24.84
Molossus rufus	Myotis nigricans	0.2500	108	35	16.34	2.53	1	17.97	27.46
Molossus rufus	Myotis occultus	0.2500	108	0	-0.31	-2.93	1	19.735	23.93
Molossus rufus	Myotis velifer	0.2500	108	20	2.97	0.67	1	20.74	21.92
Molossus rufus	Myotis volans	0.0000	108	0	-1.17	-5.61	1	20.17	23.06
Molossus rufus	Myotis yumanensis	0.3333	108	3	-0.65	-0.38	1	18.425	26.55
Molossus rufus	Nycticeius humeralis	0.5000	108	1	-0.26	-0.26	1	20.405	22.59
Molossus rufus	Nyctinomops laticaudatus	0.3333	45.8	27	19.59	3.37	1	22.35	18.7
Molossus rufus	Nyctinomops macrotis	0.3333	45.8	13	7.93	2.07	1	26.25	10.9
Molossus rufus	Parastrellus hesperus	0.5000	108	5	-0.44	-0.2	1	17.63	28.14
Molossus rufus	Phyllostomus discolor	0.0000	120	21	14.5	2.85	0	34.1	4.8
Molossus rufus	Pteronotus davyi	0.0000	120	56	19.04	2.35	1	20.59	22.22
Molossus rufus	Pteronotus parnellii	0.0000	120	101	23.98	2.22	1	25.6	12.2
Molossus rufus	Sturnira hondurensis	0.0000	120	36	11.51	1.83	0	26.3	10.8
Molossus rufus	Sturnira lilium	0.0000	120	73	24.77	2.64	0	25.95	11.5
Molossus rufus	Tadarida brasiliensis	0.1250	47.2	29	4.31	0.81	1	21.95	19.5
Molossus rufus	Trachops cirrhosus	0.0000	120	13	10.16	2.57	1	34.3	5.2
Molossus rufus	Vampyrodes caraccioli	0.0000	120	7	8.13	2.78	0	33.75	4.1
Myotis albescens	Myotis californicus	1.0000	21.8	1	1.11	1.07	1	5.02	1.26

A. Bats' characteristics of Known model

Myotis albescens	Myotis evotis	1.0000	21.8	0	-0.19	-2.01	1	6.255	1.21
Myotis albescens	Myotis nigricans	0.3333	21.8	4	8.22	2.97	1	4.945	1.41
Myotis albescens	Myotis occultus	0.3333	21.8	0	-0.07	-0.02	1	6.71	2.12
Myotis albescens	Myotis velifer	0.3333	21.8	4	4.43	1.92	1	7.715	4.13
Myotis albescens	Myotis volans	0.0000	21.8	0	-0.27	-2.71	1	7.145	2.99
Myotis albescens	Myotis yumanensis	0.5000	21.8	0	-0.5	-3.89	1	5.4	0.5
Myotis albescens	Nycticeius humeralis	1.0000	52	0	-0.27	-2.68	1	7.38	3.46
Myotis albescens	Nyctinomops laticaudatus	0.5000	108	0	-0.31	-2.97	1	9.325	7.35
Myotis albescens	Nyctinomops macrotis	0.5000	108	0	-0.34	-3.11	1	13.225	15.15
Myotis albescens	Parastrellus hesperus	1.0000	52	0	-0.58	-4.23	1	4.605	2.09
Myotis albescens	Phyllostomus discolor	0.0000	120	3	9.08	3.43	0	21.075	30.85
Myotis albescens	Pteronotus davyi	0.0000	120	3	4.2	2.06	1	7.565	3.83
Myotis albescens	Pteronotus parnellii	0.0000	120	8	8.24	2.35	1	12.575	13.85
Myotis albescens	Sturnira hondurensis	0.0000	120	6	9.16	2.79	0	13.275	15.25
Myotis albescens	Sturnira lilium	0.0000	120	3	4.05	2	0	12.925	14.55
Myotis albescens	Tadarida brasiliensis	0.1429	108	2	1.43	0.97	1	8.925	6.55
Myotis albescens	Trachops cirrhosus	0.0000	120	2	6.96	3.31	1	21.275	31.25
Myotis albescens	Vampyrodes caraccioli	0.0000	120	1	5.11	3.38	0	20.725	30.15
Myotis californicus	Myotis evotis	1.0000	21.8	5	5.26	2.2	1	5.625	2.47
Myotis californicus	Myotis nigricans	0.3333	21.2	14	5.15	1.36	1	4.315	0.15
Myotis californicus	Myotis occultus	0.3333	21.8	0	-0.31	-2.97	1	6.08	3.38
Myotis californicus	Myotis velifer	0.3333	21.8	65	16.55	1.95	1	7.085	5.39
Myotis californicus	Myotis volans	0.0000	21.8	22	17.79	3.43	1	6.515	4.25
Myotis californicus	Myotis yumanensis	0.5000	21.8	32	13.1	2.17	1	4.77	0.76
Myotis californicus	Nycticeius humeralis	1.0000	52	8	5.8	1.95	1	6.75	4.72
Myotis californicus	Nyctinomops laticaudatus	0.5000	108	1	-0.6	-0.6	1	8.695	8.61
Myotis californicus	Nyctinomops macrotis	0.5000	108	19	11.92	2.52	1	12.595	16.41
Myotis californicus	Parastrellus hesperus	1.0000	52	59	21.26	2.55	1	3.975	0.83
Myotis californicus	Phyllostomus discolor	0.0000	120	3	0.82	0.48	0	20.445	32.11
Myotis californicus	Pteronotus davyi	0.0000	120	20	4.86	1.08	1	6.935	5.09
Myotis californicus	Pteronotus parnellii	0.0000	120	36	5.83	0.97	1	11.945	15.11
Myotis californicus	Sturnira hondurensis	0.0000	120	32	9.61	1.64	0	12.645	16.51
Myotis californicus	Sturnira lilium	0.0000	120	20	4.57	1.02	0	12.295	15.81
Myotis californicus	Tadarida brasiliensis	0.1429	108	93	21.4	2.09	1	8.295	7.81

A. Bats' characteristics of Known model

Myotis californicus	Trachops cirrhosus	0.0000	120	2	0.5	0.36	1	20.645	32.51
Myotis californicus	Vampyrodes caraccioli	0.0000	120	1	0.42	0.43	0	20.095	31.41
Myotis evotis	Myotis nigricans	0.3333	21.8	1	0.88	0.87	1	5.55	2.62
Myotis evotis	Myotis occultus	0.3333	21.8	0	-0.1	-0.72	1	7.315	0.91
Myotis evotis	Myotis velifer	0.3333	21.8	3	1.67	0.93	1	8.32	2.92
Myotis evotis	Myotis volans	0.0000	21.8	5	12.56	3.63	1	7.75	1.78
Myotis evotis	Myotis yumanensis	0.5000	21.8	5	6.46	2.36	1	6.005	1.71
Myotis evotis	Nycticeius humeralis	1.0000	52	1	2.24	1.95	1	7.985	2.25
Myotis evotis	Nyctinomops laticaudatus	0.5000	108	0	-0.44	-3.66	1	9.93	6.14
Myotis evotis	Nyctinomops macrotis	0.5000	108	1	1.64	1.51	1	13.83	13.94
Myotis evotis	Parastrellus hesperus	1.0000	52	7	7.66	2.36	1	5.21	3.3
Myotis evotis	Phyllostomus discolor	0.0000	120	0	-0.45	-3.71	0	21.68	29.64
Myotis evotis	Pteronotus davyi	0.0000	120	0	-0.88	-5.05	1	8.17	2.62
Myotis evotis	Pteronotus parnellii	0.0000	120	0	-1.24	-5.74	1	13.18	12.64
Myotis evotis	Sturnira hondurensis	0.0000	120	0	-0.87	-5.02	0	13.88	14.04
Myotis evotis	Sturnira lilium	0.0000	120	0	-0.91	-5.1	0	13.53	13.34
Myotis evotis	Tadarida brasiliensis	0.1429	108	9	6.09	1.8	1	9.53	5.34
Myotis evotis	Trachops cirrhosus	0.0000	120	0	-0.39	-3.42	1	21.88	30.04
Myotis evotis	Vampyrodes caraccioli	0.0000	120	0	-0.27	-2.66	0	21.33	28.94
Myotis nigricans	Myotis occultus	0.5000	21.8	1	3.95	3.24	1	6.005	3.53
Myotis nigricans	Myotis velifer	0.5000	21.8	27	7.88	1.45	1	7.01	5.54
Myotis nigricans	Myotis volans	0.3333	21.8	1	0.16	0.17	1	6.44	4.4
Myotis nigricans	Myotis yumanensis	0.2500	21.8	5	1.34	0.6	1	4.695	0.91
Myotis nigricans	Nycticeius humeralis	0.3333	52	3	2.4	1.34	1	6.675	4.87
Myotis nigricans	Nyctinomops laticaudatus	0.2500	108	13	11.45	2.71	1	8.62	8.76
Myotis nigricans	Nyctinomops macrotis	0.2500	108	9	6.91	2.08	1	12.52	16.56
Myotis nigricans	Parastrellus hesperus	0.3333	52	4	0.06	0.03	1	3.9	0.68
Myotis nigricans	Phyllostomus discolor	0.0000	120	17	14.86	3	0	20.37	32.26
Myotis nigricans	Pteronotus davyi	0.0000	120	31	12.8	2.08	1	6.86	5.24
Myotis nigricans	Pteronotus parnellii	0.0000	120	75	22.59	2.31	1	11.87	15.26
Myotis nigricans	Sturnira hondurensis	0.0000	120	43	18.88	2.5	0	12.57	16.66
Myotis nigricans	Sturnira lilium	0.0000	120	53	22.63	2.66	0	12.22	15.96
Myotis nigricans	Tadarida brasiliensis	0.1111	108	23	4.95	1.02	1	8.22	7.96
Myotis nigricans	Trachops cirrhosus	0.0000	120	10	9.89	2.68	1	20.57	32.66

A. Bats' characteristics of Known model

Myotis nigricans	Vampyrodes caraccioli	0.0000	120	8	12.04	3.44	0	20.02	31.56
Myotis occultus	Myotis velifer	0.5000	21.8	1	2.08	1.83	1	8.775	2.01
Myotis occultus	Myotis volans	0.3333	21.8	0	-0.14	-1.41	1	8.205	0.87
Myotis occultus	Myotis yumanensis	0.2500	21.8	0	-0.26	-2.6	1	6.46	2.62
Myotis occultus	Nycticeius humeralis	0.3333	52	0	-0.14	-1.39	1	8.44	1.34
Myotis occultus	Nyctinomops laticaudatus	0.2500	108	0	-0.16	-1.67	1	10.385	5.23
Myotis occultus	Nyctinomops macrotis	0.2500	108	0	-0.17	-1.82	1	14.285	13.03
Myotis occultus	Parastrellus hesperus	0.3333	52	0	-0.31	-2.93	1	5.665	4.21
Myotis occultus	Phyllostomus discolor	0.0000	120	0	-0.17	-1.72	0	22.135	28.73
Myotis occultus	Pteronotus davyi	0.0000	120	0	-0.33	-3.05	1	8.625	1.71
Myotis occultus	Pteronotus parnellii	0.0000	120	0	-0.46	-3.74	1	13.635	11.73
Myotis occultus	Sturnira hondurensis	0.0000	120	1	2.8	2.28	0	14.335	13.13
Myotis occultus	Sturnira lilium	0.0000	120	0	-0.33	-3.11	0	13.985	12.43
Myotis occultus	Tadarida brasiliensis	0.1111	108	2	3.95	2.27	1	9.985	4.43
Myotis occultus	Trachops cirrhosus	0.0000	120	0	-0.14	-1.43	1	22.335	29.13
Myotis occultus	Vampyrodes caraccioli	0.0000	120	0	-0.1	-0.67	0	21.785	28.03
Myotis velifer	Myotis volans	0.3333	21.8	15	8.43	2.21	1	9.21	1.14
Myotis velifer	Myotis yumanensis	0.2500	21.8	49	15.21	2.2	1	7.465	4.63
Myotis velifer	Nycticeius humeralis	0.3333	52	24	14.64	3.1	1	9.445	0.67
Myotis velifer	Nyctinomops laticaudatus	0.2500	108	11	4.65	1.43	1	11.39	3.22
Myotis velifer	Nyctinomops macrotis	0.2500	108	27	12.78	2.5	1	15.29	11.02
Myotis velifer	Parastrellus hesperus	0.3333	52	52	12.86	1.81	1	6.67	6.22
Myotis velifer	Phyllostomus discolor	0.0000	120	5	1.01	0.47	0	23.14	26.72
Myotis velifer	Pteronotus davyi	0.0000	120	34	6.37	1.12	1	9.63	0.3
Myotis velifer	Pteronotus parnellii	0.0000	120	72	9.88	1.2	1	14.64	9.72
Myotis velifer	Sturnira hondurensis	0.0000	120	53	12.19	1.71	0	15.34	11.12
Myotis velifer	Sturnira lilium	0.0000	120	36	6.56	1.13	0	14.99	10.42
Myotis velifer	Tadarida brasiliensis	0.1111	108	163	29.13	2.31	1	10.99	2.42
Myotis velifer	Trachops cirrhosus	0.0000	120	45	-0.94	-0.91	1	23.34	27.12
Myotis velifer	Vampyrodes caraccioli	0.0000	120	21	-1.09	-5.48	0	22.79	26.02
Myotis volans	Myotis yumanensis	0.0000	21.8	11	10.16	2.49	1	6.895	3.49
Myotis volans	Nycticeius humeralis	0.0000	52	0	-0.54	-4.08	1	8.875	0.47
Myotis volans	Nyctinomops laticaudatus	0.0000	108	0	-0.62	-4.36	1	10.82	4.36
Myotis volans	Nyctinomops macrotis	0.0000	108	3	3.82	1.94	1	14.72	12.16

A. Bats' characteristics of Known model

Myotis volans	Parastrellus hesperus	0.0000	52	12	9.13	2.23	1	6.1	5.08
Myotis volans	Phyllostomus discolor	0.0000	120	0	-0.64	-4.41	0	22.57	27.86
Myotis volans	Pteronotus davyi	0.0000	120	1	-0.44	-0.44	1	9.06	0.84
Myotis volans	Pteronotus parnellii	0.0000	120	4	0.52	0.26	1	14.07	10.86
Myotis volans	Sturnira hondurensis	0.0000	120	4	2.03	0.99	0	14.77	12.26
Myotis volans	Sturnira lilium	0.0000	120	2	0.28	0.2	0	14.42	11.56
Myotis volans	Tadarida brasiliensis	0.0000	108	27	13.81	2.24	1	10.42	3.56
Myotis volans	Trachops cirrhosus	0.0000	120	0	-0.55	-4.12	1	22.77	28.26
Myotis volans	Vampyrodes caraccioli	0.0000	120	0	-0.38	-3.36	0	22.22	27.16
Myotis yumanensis	Nycticeius humeralis	0.5000	52	2	1.08	0.76	1	7.13	3.96
Myotis yumanensis	Nyctinomops laticaudatus	0.3333	108	0	-1.14	-5.56	1	9.075	7.85
Myotis yumanensis	Nyctinomops macrotis	0.3333	108	11	7.96	2.17	1	12.975	15.65
Myotis yumanensis	Parastrellus hesperus	0.5000	52	34	14.12	2.19	1	4.355	1.59
Myotis yumanensis	Phyllostomus discolor	0.0000	120	0	-1.17	-5.61	0	20.825	31.35
Myotis yumanensis	Pteronotus davyi	0.0000	120	11	2.66	0.8	1	7.315	4.33
Myotis yumanensis	Pteronotus parnellii	0.0000	120	23	4.11	0.85	1	12.325	14.35
Myotis yumanensis	Sturnira hondurensis	0.0000	120	11	2.76	0.83	0	13.025	15.75
Myotis yumanensis	Sturnira lilium	0.0000	120	17	5.08	1.2	0	12.675	15.05
Myotis yumanensis	Tadarida brasiliensis	0.1250	108	62	16.72	1.96	1	8.675	7.05
Myotis yumanensis	Trachops cirrhosus	0.0000	120	0	-1.01	-5.32	1	21.025	31.75
Myotis yumanensis	Vampyrodes caraccioli	0.0000	120	0	-0.69	-4.56	0	20.475	30.65
Nycticeius humeralis	Nyctinomops laticaudatus	0.5000	108	5	7.54	2.67	1	11.055	3.89
Nycticeius humeralis	Nyctinomops macrotis	0.5000	108	5	6.91	2.51	1	14.955	11.69
Nycticeius humeralis	Parastrellus hesperus	1.0000	52	4	2.31	1.11	1	6.335	5.55
Nycticeius humeralis	Phyllostomus discolor	0.0000	120	0	-0.63	-4.39	0	22.805	27.39
Nycticeius humeralis	Pteronotus davyi	0.0000	120	4	2.03	0.98	1	9.295	0.37
Nycticeius humeralis	Pteronotus parnellii	0.0000	120	6	1.72	0.7	1	14.305	10.39
Nycticeius humeralis	Sturnira hondurensis	0.0000	120	1	-0.39	-0.39	0	15.005	11.79
Nycticeius humeralis	Sturnira lilium	0.0000	120	1	-0.48	-0.47	0	14.655	11.09
Nycticeius humeralis	Tadarida brasiliensis	0.1429	108	22	11.1	2.05	1	10.655	3.09
Nycticeius humeralis	Trachops cirrhosus	0.0000	120	0	-0.55	-4.1	1	23.005	27.79
Nycticeius humeralis	Vampyrodes caraccioli	0.0000	120	0	-0.37	-3.34	0	22.455	26.69
Nyctinomops laticaudatus	Nyctinomops macrotis	0.3333	20.8	5	5.82	2.22	1	16.9	7.8
Nyctinomops laticaudatus	Parastrellus hesperus	0.5000	108	1	-0.58	-0.57	1	8.28	9.44

A. Bats' characteristics of Known model

Nyctinomops laticaudatus	Phyllostomus discolor	0.0000	120	5	6.18	2.33	0	24.75	23.5
Nyctinomops laticaudatus	Pteronotus davyi	0.0000	120	15	9.21	2.07	1	11.24	3.52
Nyctinomops laticaudatus	Pteronotus parnellii	0.0000	120	36	16.08	2.27	1	16.25	6.5
Nyctinomops laticaudatus	Sturnira hondurensis	0.0000	120	5	2.19	0.95	0	16.95	7.9
Nyctinomops laticaudatus	Sturnira lilium	0.0000	120	18	10.95	2.21	0	16.6	7.2
Nyctinomops laticaudatus	Tadarida brasiliensis	0.1250	47.2	14	5.11	1.29	1	12.6	0.8
Nyctinomops laticaudatus	Trachops cirrhosus	0.0000	120	5	7.35	2.64	1	24.95	23.9
Nyctinomops laticaudatus	Vampyrodes caraccioli	0.0000	120	0	-0.43	-3.62	0	24.4	22.8
Nyctinomops macrotis	Parastrellus hesperus	0.5000	108	20	12.6	2.37	1	12.18	17.24
Nyctinomops macrotis	Phyllostomus discolor	0.0000	120	2	1.78	1.21	0	28.65	15.7
Nyctinomops macrotis	Pteronotus davyi	0.0000	120	19	10.99	2.18	1	15.14	11.32
Nyctinomops macrotis	Pteronotus parnellii	0.0000	120	28	10.92	1.85	1	20.15	1.3
Nyctinomops macrotis	Sturnira hondurensis	0.5000	120	12	6.5	1.71	0	20.85	0.1
Nyctinomops macrotis	Sturnira lilium	0.1667	120	17	9.32	2	0	20.5	0.6
Nyctinomops macrotis	Tadarida brasiliensis	0.1250	47.2	44	18.6	2.36	1	16.5	8.6
Nyctinomops macrotis	Trachops cirrhosus	0.3333	120	1	0.8	0.8	1	28.85	16.1
Nyctinomops macrotis	Vampyrodes caraccioli	0.0000	120	0	-0.47	-3.77	0	28.3	15
Parastrellus hesperus	Phyllostomus discolor	0.0000	120	0	-1.39	-5.95	0	20.03	32.94
Parastrellus hesperus	Pteronotus davyi	0.0000	120	15	3.02	0.79	1	6.52	5.92
Parastrellus hesperus	Pteronotus parnellii	0.0000	120	20	1.6	0.36	1	11.53	15.94
Parastrellus hesperus	Sturnira hondurensis	0.0000	120	10	1.2	0.39	0	12.23	17.34
Parastrellus hesperus	Sturnira lilium	0.0000	120	17	3.53	0.86	0	11.88	16.64
Parastrellus hesperus	Tadarida brasiliensis	0.1428	108	87	19.97	2.02	1	7.88	8.64
Parastrellus hesperus	Trachops cirrhosus	0.0000	120	0	-1.2	-5.66	1	20.23	33.34
Parastrellus hesperus	Vampyrodes caraccioli	0.0000	120	0	-0.82	-4.9	0	19.68	32.24
Phyllostomus discolor	Pteronotus davyi	0.0000	85	23	14.43	2.48	0	22.99	27.02
Phyllostomus discolor	Pteronotus parnellii	0.0000	85	42	18.51	2.39	0	28	17
Phyllostomus discolor	Sturnira hondurensis	0.0000	55.6	22	13.97	2.46	0	28.7	15.6
Phyllostomus discolor	Sturnira lilium	0.0000	55.6	31	19.34	2.76	1	28.35	16.3
Phyllostomus discolor	Tadarida brasiliensis	0.0000	120	4	-0.07	-0.03	0	24.35	24.3
Phyllostomus discolor	Trachops cirrhosus	0.0000	45.6	23	35.13	4.72	0	36.7	0.4
Phyllostomus discolor	Vampyrodes caraccioli	0.0000	55.6	7	15.5	3.98	0	36.15	0.7
Pteronotus davyi	Pteronotus parnellii	0.1428	36.2	152	34.63	2.62	1	14.49	10.02
Pteronotus davyi	Sturnira hondurensis	0.0000	85	39	11.35	1.76	0	15.19	11.42

A. Bats' characteristics of Known model

Pteronotus davyi	Sturnira lilium	0.0000	85	84	26.39	2.68	0	14.84	10.72
Pteronotus davyi	Tadarida brasiliensis	0.0000	120	39	5.98	0.97	1	10.84	2.72
Pteronotus davyi	Trachops cirrhosus	0.0000	85	18	13.27	2.9	1	23.19	27.42
Pteronotus davyi	Vampyrodes caraccioli	0.0000	85	6	6.23	2.39	0	22.64	26.32
Pteronotus parnellii	Sturnira hondurensis	0.0000	85	100	22.19	2.39	0	20.2	1.4
Pteronotus parnellii	Sturnira lilium	0.0000	85	137	30.29	2.86	0	19.85	0.7
Pteronotus parnellii	Tadarida brasiliensis	0.0000	120	88	10.52	1.18	1	15.85	7.3
Pteronotus parnellii	Trachops cirrhosus	0.0000	85	34	18	3.71	1	28.2	17.4
Pteronotus parnellii	Vampyrodes caraccioli	0.0000	85	12	9	2.87	0	27.65	16.3
Sturnira hondurensis	Sturnira lilium	0.2000	15	66	20.44	2.36	0	20.55	0.7
Sturnira hondurensis	Tadarida brasiliensis	0.0000	120	56	10.59	1.4	0	16.55	8.7
Sturnira hondurensis	Trachops cirrhosus	0.5000	55.6	11	7.75	2.21	0	28.9	16
Sturnira hondurensis	Vampyrodes caraccioli	0.0000	34.2	8	8.73	2.85	1	28.35	14.9
Sturnira lilium	Tadarida brasiliensis	0.0000	120	39	5.6	0.91	0	16.2	8
Sturnira lilium	Trachops cirrhosus	0.1666	55.6	24	17.58	3.39	0	28.55	16.7
Sturnira lilium	Vampyrodes caraccioli	0.0000	34.2	15	16.39	4.17	0	28	15.6
Tadarida brasiliensis	Trachops cirrhosus	0.0000	120	2	-0.65	-0.46	1	24.55	24.7
Tadarida brasiliensis	Vampyrodes caraccioli	0.0000	120	0	-1.25	-5.74	0	24	23.6
Trachops cirrhosus	Vampyrodes caraccioli	0.0000	55.6	7	18	4.27	0	36.35	1.1

A. Bats' community of Potential model

	Alphacoronavirus	Alphapolyomavirus	Avulavirus	Betacoronavirus	Betapolyomavirus	Flavivirus	Hantavirus	Lyssavirus	Morbillivirus	Pegivirus	Rotavirus	Rubulavirus
Anoura.geoffroyi	1	0	0	0	0	0	0	0	0	0	0	0
Antrozous.pallidus	0	0	0	0	0	0	0	1	0	0	0	0
Artibeus.jamaicensis	1	0	0	0	0	1	0	1	0	0	0	1
Artibeus.lituratus	1	0	1	1	0	1	0	1	0	0	0	0
Carollia.perspicillata	1	1	0	1	0	1	0	0	1	1	1	0
Carollia.sowelli	1	0	0	0	0	0	1	0	0	0	0	0
Choeroniscus.godmani	0	0	0	0	0	0	0	0	0	1	0	0
Corynorhinus.townsendii	0	0	0	0	0	0	0	1	0	0	0	0
Dermanura.phaeotis	0	0	1	1	0	0	0	0	0	0	0	0
Dermanura.tolteca	0	0	0	0	0	0	0	0	0	1	0	0
Dermanura.watsoni	0	0	0	0	0	0	0	0	0	1	0	0
Desmodus.rotundus	1	0	0	0	1	0	0	1	1	1	0	1
Diphylla.ecaudata	0	0	0	0	0	0	1	1	0	0	0	0
Eptesicus.brasiliensis	0	0	0	0	0	0	0	1	0	0	0	0
Eptesicus.furinalis	0	0	0	0	0	0	0	1	0	0	0	0
Eptesicus.fuscus	1	0	0	0	0	0	0	1	0	0	0	0
Euderma.maculatum	0	0	0	0	0	0	0	1	0	0	0	0
Eumops.auripendulus	0	0	0	0	0	0	0	1	0	0	0	0
Eumops.ferox	0	0	0	1	0	0	0	1	0	0	0	0
Eumops.nanus	0	0	0	0	0	0	0	1	0	0	0	0
Eumops.perotis	0	0	0	0	0	0	0	1	0	0	0	0
Glossophaga.commissarisi	0	0	0	0	0	0	0	0	0	1	0	0
Glossophaga.soricina	1	0	0	0	0	1	0	1	1	0	1	0
Lasionycteris.noctivagans	0	0	0	0	0	0	0	1	0	0	0	0
Lasiurus.blossevillii	0	0	0	0	0	0	0	1	0	0	0	0
Lasiurus.borealis	0	0	0	0	0	0	0	1	0	0	0	0
Lasiurus.cinereus	0	0	0	0	0	0	0	1	0	0	0	0
Lasiurus.ega	0	0	0	0	0	0	0	1	0	0	0	0
Lasiurus.intermedius	0	0	0	0	0	0	0	1	0	0	0	0

A. Bats' community of Potential model

Lasiurus.xanthinus	0	0	0	0	0	0	0	1	0	0	0	0
Lonchorhina.aurita	1	0	0	0	0	0	0	0	0	0	0	0
Molossus.molossus	0	1	0	0	0	0	0	1	0	0	1	0
Molossus.rufus	1	0	0	0	0	0	0	1	0	0	0	0
Myotis.albescens	0	0	0	0	0	0	0	1	0	0	0	0
Myotis.californicus	0	0	0	0	0	0	0	1	0	0	0	0
Myotis.evotis	0	0	0	0	0	0	0	1	0	0	0	0
Myotis.nigricans	1	0	0	0	0	1	0	1	0	0	0	0
Myotis.occultus	1	0	0	0	1	0	0	1	0	0	0	0
Myotis.velifer	1	0	0	0	0	0	0	1	0	0	0	0
Myotis.volans	1	0	0	0	0	0	0	0	0	0	0	0
Myotis.yumanensis	0	0	0	0	0	0	0	1	0	0	0	0
Nycticeius.humeralis	0	0	0	0	0	0	0	1	0	0	0	0
Nyctinomops.laticaudatus	0	0	0	1	0	0	0	1	0	0	0	0
Nyctinomops.macrotis	0	0	0	0	0	0	0	1	0	1	0	0
Parastrellus.hesperus	0	0	0	0	0	0	0	1	0	0	0	0
Phyllostomus.discolor	1	0	0	0	0	0	0	0	0	0	0	0
Pteronotus.davyi	0	0	0	1	1	0	0	0	0	0	0	0
Pteronotus.parnellii	0	0	0	1	1	1	0	0	1	0	0	1
Sturnira.hondurensis	0	0	0	0	0	0	0	0	0	1	0	0
Sturnira.lilium	1	1	0	0	0	0	0	0	0	1	0	1
Tadarida.brasiliensis	1	0	0	0	0	1	0	1	0	0	0	0
Trachops.cirrhosus	0	0	0	0	0	0	1	0	0	1	0	0
